# Association of low birth weight with cardiometabolic diseases in Swedish twins: a population-based cohort study

**DOI:** 10.1136/bmjopen-2020-048030

**Published:** 2021-06-28

**Authors:** Xuerui Li, Rongrong Yang, Wenzhe Yang, Hui Xu, Ruixue Song, Xiuying Qi, Weili Xu

**Affiliations:** 1 Department of Epidemiology and Biostatistics, School of Public Health, Tianjin Medical University, Tianjin, China; 2 Tianjin Key Laboratory of Environment, Nutrition and Public Health, Tianjin, China; 3 Center for International Collaborative Research on Environment, Nutrition and Public Health, Tianjin, China; 4 Public Health Science and Engineering College, Tianjin University of Traditional Chinese Medicine, Tianjin, China; 5 Big Data and Engineering Research Center, Beijing Children's Hospital, Capital Medical University, National Center for Children's Health, Beijing, China; 6 Aging Research Center, Department of Neurobiology, Health Care Sciences and Society, Karolinska Institutet and Stockholm University, Stockholm, Sweden

**Keywords:** epidemiology, public health, adult cardiology, general diabetes

## Abstract

**Objectives:**

To examine the association between low birth weight (LBW) and cardiometabolic diseases (CMDs, including heart disease, stroke and type 2 diabetes mellitus) in adulthood, and to explore whether genetic, early-life environmental and healthy lifestyle factors play a role in this association.

**Design:**

A population-based twin study.

**Setting:**

Twins from the Swedish Twin Registry who were born in 1958 or earlier participated in the Screening Across the Lifespan Twin (SALT) study for a full-scale screening during 1998–2002 and were followed up until 2014.

**Participants:**

19 779 twin individuals in Sweden with birthweight data available (mean age: 55.45 years).

**Primary and secondary outcome measures:**

CMDs were assessed based on self-reported medical records, medication use and records from the National Patient Registry. A lifestyle index encompassing smoking status, alcohol consumption, exercise levels and Body Mass Index was derived from the SALT survey and categorised as unfavourable, intermediate or favourable. Data were analysed using generalised estimating equation (GEE) models and conditional logistic regression models.

**Results:**

Of all participants, 3998 (20.2%) had LBW and 5335 (27.0%) had incident CMDs (mean age at onset: 63.64±13.26 years). In GEE models, the OR of any CMD was 1.39 (95% CI 1.27 to 1.52) for LBW. In conditional logistic regression models, the LBW–CMD association became non-significant (OR=1.21, 95% CI 0.94 to 1.56). The difference in ORs from the two models was statistically significant (p<0.001). In the joint effect analysis, the multiadjusted OR of CMDs was 3.47 (95% CI 2.72 to 4.43) for participants with LBW plus an unfavourable lifestyle and 1.25 (95% CI 0.96 to 1.62) for those with LBW plus a favourable lifestyle.

**Conclusion:**

LBW is associated with an increased risk of adult CMDs, and genetic and early-life environmental factors may account for this association. However, a favourable lifestyle profile may modify this risk.

**Strengths and limitations of this study**

- This study provides an extraordinary opportunity to explore the association between low birth weight (LBW) and cardiometabolic diseases (CMDs) by using a twin study design to control for some unmeasured confounders.
- The investigation into factors that might compensate for the risk effect of LBW on CMDs is unique.
- Birth weight was based on self-reports, and non-differential misclassification among different birthweight groups could not be ruled out, possibly leading to an underestimation of the observed associations.
- Some prenatal factors (such as gestational age, maternal smoking during pregnancy or premature birth) could not be controlled for, as information on these factors was not available.
- Potential variations of lifestyle factors during the follow-up also could not be assessed.

## Introduction

With population ageing has come an increase in the prevalence of chronic diseases, especially heart diseases (ie, coronary heart diseases and heart failure), stroke and type 2 diabetes mellitus (T2DM).^1^ According to the WHO, heart diseases and stroke, so-called cardiovascular disease (CVD), are the leading cause of disease burden and death worldwide.^2 3^ About 17.6 million deaths were attributed to CVD globally in 2016.^2^ Meanwhile, there were 451 million adults living with diabetes worldwide in 2017 (90% of whom had T2DM), and this number is projected to increase to 693 million by 2045.^4 5^ All of these co-occurring chronic diseases have been defined as cardiometabolic diseases (CMDs).^6 7^

Recently, beyond the effects of some traditional risk factors including age, smoking, drinking and Body Mass Index (BMI) on individual CMDs, the role of early-life experiences in the future development of chronic diseases have drawn special attention.^8^ Birth weight, an early-life indicator,^9^ is frequently used to explore the effects of early-life experiences on the risk of individual CMDs in adulthood. Several cohort studies have shown that low birth weight (LBW) is associated with an increased risk of coronary heart disease,^10^ stroke^11^ and T2DM,^12 13^ but with some inconsistent findings.^14 15^ Moreover, many studies have examined the relationship between birth weight and metabolic syndrome with inconsistent results,^16–18^ but no studies have investigated the association of LBW with the risk of CMDs.

CMDs are complex genetic and lifestyle-related disorders,^19–21^ and birth weight may also be affected by genetic factors and intrauterine environment.^22^ However, the role of the genetic and early-life environmental factors (another term for shared environmental factors), such as intrauterine environment and prenatal nutritional status, in the association between birth weight and CMDs remains unclear. Twin studies make it possible to minimise potential confounding effects of unmeasured genetic predisposition and shared early-life environment when comparisons are made between twins.^23 24^ Apart from genetic factors, some modifiable lifestyle factors such as not smoking, moderate alcohol consumption, engagement in physical activities and maintaining a healthy weight have been reported to be linked to a lower risk of CVD or T2DM.^25 26^ However, previous population-based cohort studies have only shown that healthy lifestyle (such as active physical activity, not smoking, moderate alcohol consumption and BMI<25) may reduce the risk effect of LBW on the development of diabetes.^27 28^ Questions remain regarding whether and to what extent healthy lifestyle may mitigate the risk of LBW on CMDs more widely.

In the present study, we aimed to (1) verify the relationship between LBW and risk of CMDs using population-based Sweden twin data and (2) explore whether genetic, early-life environmental and healthy lifestyle factors play a role in this association.

## Methods

### Study population

This prospective, nested case–control study included twins from the nationwide Swedish Twin Registry (STR), which started in the 1960s.^29^ From 1998 to 2002, all living twins born in 1958 or earlier were recruited to participate in the Screening Across the Lifespan Twin (SALT) study, a full-scale screening through a computer-assisted telephone interview. Of the 19 940 twin individuals in the SALT study with birthweight data available, we excluded 53 individuals with birth weights that were outliers (extreme values; ie, birth weight of ≤300 g or ≥4520 g) to minimise possible misclassification and 108 individuals with type 1 diabetes. Finally, 19 779 individuals were included in the current study (online supplemental figure S1).

10.1136/bmjopen-2020-048030.supp1 Supplementary data

### Data collection

Data on age, sex, educational attainment, marital status and zygosity status were collected through the SALT survey.^29^ Zygosity status was categorised as monozygotic, dizygotic or undetermined zygosity on the basis of self-reported information about childhood resemblance, which was validated against biological markers with 95%–99% accuracy.^29^ Education was dichotomised into <8 vs ≥8 years according to the number of years of formal schooling attained. Marital status was classified into married/cohabitating vs single (including divorced or widows/widowers).

Information on medical conditions including heart disease, stroke, T2DM and hypertension was derived from the National Patient Registry (NPR), which covers all inpatient diagnoses in Sweden from the 1960s and outpatient (specialist clinic) diagnoses from 2001 to 2014.^30^ Each medical record in the NPR included up to eight discharge diagnoses according to the International Classification of Disease (ICD) codes. The ICD, 7th Revision, was used through 1968, the 8th Revision from 1969 to 1986, the 9th Revision from 1987 to 1996 and the 10th revision from 1997 to the end of 2014.

### Assessment of birth weight

Data on birth weight was collected based on self-reports from SALT or STR. Generally, LBW was defined as birth weight of <2500 g in singletons.^31^ However, twins may experience a more unfavourable intrauterine environment, causing them to have a lower birth weight (on average 800 g) than singletons.^32^ Thus, birth weight in the present study was categorised as <2 kg (LBW), 2–3 kg (moderate birth weight (MBW)) or >3 kg (high birth weight (HBW)),^32^ considering its distribution.

### Ascertainment of CMD

In the current analysis, CMDs included heart disease (coronary heart disease and heart failure), stroke (ischaemic stroke and haemorrhagic stroke) and T2DM, all of which were diagnosed based on self-reported medical records, medication use and NPR data. The detailed ICD codes for each disease are shown in the online supplemental table S1.

CMD status was categorised as CMD-free and any CMD (ie, presence any of heart disease, stroke and/or T2DM). The any CMD group was further classified as only one CMD (heart disease, stroke or T2DM), any two CMDs (any two of the following: heart disease, stroke and T2DM), and three or more CMDs (heart disease, stroke and T2DM).

### Assessment of lifestyle-related factors

Information on lifestyle factors (including smoking status, alcohol consumption, physical exercise and BMI) was obtained from the SALT survey. In detail, smoking status was dichotomised as non-smoking versus former/current smoker. Alcohol consumption was categorised as no/mild drinking versus heavy drinking based on the survey question asking whether participants have ever drunk excessively over a period. Data on physical exercise were collected by a question on average exercise with seven response options: (1) ‘almost never’, (2) ‘much less than average’, (3) ‘less than average’, (4) ‘average’, (5) ‘more than average’, (6) ‘much more than average’ and (7) ‘maximum’^33^ and was dichotomised as ‘inactive’, including the first four groups (1–4) and ‘active’, including the last three groups (5–7). BMI in adulthood (mean age 55.45±9.05) was calculated as weight (kg) divided by squared height (m^2^) and classified as underweight (<18.5), normal weight (18.5–24.9), overweight (25.0–29.9) and obesity (≥30) according to the WHO classification. Obesity was merged with overweight (hereafter overweight; ie, BMI≥25), and underweight was merged with normal weight as non-overweight (BMI<25).

In the current study, on the basis of the data availability, the following four factors were considered as healthy lifestyle factors: (1) non-smoking, (2) no/mild alcohol consumption, (3) active physical exercise and (4) non-overweight in adulthood.^34^ The four factors were combined into a lifestyle index with a score ranging from 0 to 4, with one point representing each factor. Participants were categorised according to their score of lifestyle index: (1) unfavourable (score: 0–1): participants who had no healthy lifestyle factors or only one, (2) intermediate (score: 2–3): those who had two or three healthy lifestyle factors, and (3) favourable (score: 4) those who had all the healthy lifestyle factors.

### Statistical analyses

The characteristics of participants in different groups were compared using χ^2^ tests for categorical variables and one-way analysis of variance/Kruskal-Wallis H test for continuous variables. Missing values on education level (n=92), smoking status (n=77), alcohol consumption (n=117), marital status (n=2), physical exercise (n=1179) and BMI (n=290) were imputed using Rubin’s rule for pooling estimates to obtain valid statistical inferences.^24^

In our study, two analytical strategies were applied. First, generalised estimating equation (GEE) models were used for unmatched case–control analysis. GEE models are conceptually equivalent to logistic regression for the analysis of classic case–control design but control for the clustering of twins within a pair. Second, conditional logistic regression models were used for cotwin matched case–control analysis using a pair of twins that was discordant for the outcome. Cotwin matched design (especially in monozygotic twins) appeared more informative since cases and controls were comparable with respect to genetic background and early-life environmental factors such as intrauterine environment, prenatal and postnatal nutritional status, and childhood socioeconomic status.^35 36^ In both GEE and conditional logistic regression, the ORs and 95% CIs were estimated for the association between birth weight (reference: MBW) and CMDs. Logistic regression was used to test the difference in ORs from GEE and conditional logistic regression models by examining the difference in the proportions of birth weight between unmatched controls and cotwin matched controls.^36^ If an OR for the observed association becomes strengthened or attenuated (or even disappears) in cotwin control analyses compared with that in the unmatched case–control analysis, and the difference in ORs from the two models is significant, then genetic and/or early-life environmental factors are likely to play a role in the association.^24 35 37^ If the ORs are similar between the two models without a statistically significant difference, then the effect of genetic and/or early-life environmental factors in the association can be neglected.^23 36^ We hypothesised that LBW would be a significant risk factor for CMDs in a classical case-control analysis, but that the association between LBW and CMDs would be attenuated in the cotwin-matched analysis after controlling for genetic, maternal and environmental factors shared by twins. Logistic regression was used to test the difference in ORs from the GEE model and conditional logistic regression.

Considering information on lifestyle factors was obtained from the SALT questionnaire during 1998–2002, we excluded 1748 participants who developed CMDs before the SALT recruitment, and thus 18 031 participants remained for the joint effect analysis. The combined effect of the LBW (no vs yes) and lifestyle index (unfavourable/intermediate/favourable) on the risk of CMDs was assessed by creating dummy variables based on the joint exposures to both factors. The presence of an additive interaction was examined by estimating relative excess risk due to interaction, the attributable proportion (AP) and the Synergy Index (S).

All the models were basic adjusted for age, sex and education, and further adjusted for smoking, alcohol consumption, marital status, physical exercise, BMI and hypertension. P values less than 0.05 were considered statistically significant. All statistical analyses were performed using SAS statistical software V.9.4 and IBM SPSS Statistics V.20.0.

### Patient and public involvement

Patients and the public were not involved in the design, or conduct, or reporting of this study.

## Results

### Characteristics of the study population

Among all participants (n=19 779), 3998 (20.2%) had LBW. The average age at recruitment was 55.45 (±9.05) years. Compared with MBW individuals, those with LBW were more likely to be older, male, monozygotic twins, single, have lower education, have higher BMI, be physically inactive and have hypertension. Participants who had HBW were more likely to be male, dizygotic twins, smokers, heavy drinkers and have higher BMI (table 1).

**Table 1:** Characteristics of the study population (N=19 779) by birth weight

Characteristics	<2.0 kg n=3998	2.0–3.0 kg n=11 510	>3.0 kg n=4271	P value
Age (years), mean (SD)	57.37 (9.6)	55.07 (8.8)	54.70 (8.9)	<0.001
Male sex, n (%)	1307 (32.7)	3504 (30.4)	2042 (47.8)	<0.001
Education, n (%)				<0.001
<8 years	1251 (31.3)	2850 (24.8)	1009 (23.6)	
≥8 years	2747 (68.7)	8660 (75.2)	3262 (76.4)	
Marital status, n (%)				<0.001
Married/cohabited	2911 (72.8)	8749 (76.0)	3298 (77.2)	
Single	1087 (27.2)	2761 (24.0)	973 (22.8)	
Zygosity, n (%)				<0.001
Monozygosity	1027 (25.7)	2647 (23.0)	685 (16.0)	
Dizygosity	2384 (59.6)	7436 (64.6)	3021 (70.7)	
Undetermined	587 (14.7)	1427 (12.4)	565 (13.2)	
BMI, mean (SD)	25.02 (3.8)	24.67 (3.5)	25.13 (3.5)	<0.001
BMI, n (%)				<0.001
<18.5 (underweight)	71 (1.8)	167 (1.4)	46 (1.1)	
18.5–24.9 (normal weight)	2108 (52.7)	6600 (57.3)	2218 (52.0)	
25.0–29.9 (overweight)	1439 (36.0)	3874 (33.7)	1623 (38.0)	
≥30 (obese)	380 (9.5)	869 (7.6)	384 (9.0)	
Smoking status, n (%)				<0.001
Never smoked	2049 (51.2)	5825 (50.6)	1932 (45.2)	
Former/current smoker	1949 (48.8)	5685 (49.4)	2339 (54.8)	
Alcohol consumption, n (%)				<0.001
No/mild drinking	3735 (93.4)	10 746 (93.4)	3884 (90.9)	
Heavy drinking	263 (6.6)	764 (6.6)	387 (9.1)	
Active physical exercise, n (%)				0.008
No	2092 (52.3)	5736 (49.8)	2101 (49.2)	
Yes	1905 (48.2)	5774 (50.2)	2170 (50.8)	
Hypertension, n (%)	1299 (33.5)	2954 (25.7)	1023 (24.0)	<0.001

Data are presented as means±SD or number (%).

BMI, body mass index.

#### Association between birth weight and CMDs in unmatched case–control analysis

In the multiadjusted GEE model, compared with participants with MBW, those with LBW had a significantly higher risk of coronary heart disease, heart failure, ischaemic stroke and T2DM, which were further combined as CMDs (n=5335), as shown in table 2. LBW was associated with an increased risk of any CMD (OR 1.39, 95% CI 1.27 to 1.52). However, HBW was not significantly associated with any CMD (OR 1.05, 95% CI 0.96 to 1.16). Therefore, MBW and HBW were combined into non-LBW group as reference in the following analysis.

**Table 2:** ORs and 95% CIs of birth weight in relation to different subtypes of heart diseases, stroke and diabetes in adulthood: results from generalised estimating equation

Single/combined CMDs	Cases (n)	OR (95% CI)*	OR (95% CI)†
Subtypes of heart disease			
CHD			
<2	622	1.33 (1.19 to 1.49)	1.27 (1.14 to 1.43)
2–3	1166	Reference	Reference
>3	497	1.07 (0.95 to 1.20)	1.08 (0.95 to 1.22)
HF			
<2	214	1.36 (1.13 to 1.63)	1.27 (1.05 to 1.53)
2–3	356	Reference	Reference
>3	143	1.13 (0.93 to 1.39)	1.12 (0.91 to 1.38)
Subtypes of stroke			
IS			
<2	432	1.20 (1.06 to 1.36)	1.14 (1.01 to 1.30)
2–3	874	Reference	Reference
>3	352	1.10 (0.96 to 1.26)	1.12 (0.98 to 1.29)
HS			
<2	74	1.14 (0.86 to 1.50)	1.09 (0.82 to 1.44)
2–3	162	Reference	Reference
>3	59	0.97 (0.72 to 1.32)	0.99 (0.73 to 1.34)
T2DM			
<2	668	1.45 (1.30 to 1.61)	1.39 (1.24 to 1.55)
2–3	1219	Reference	Reference
>3	424	0.88 (0.78 to 0.99)	0.82 (0.72 to 0.93)
Any CMD (CHD, HF, IS and T2DM)			
<2	1423	1.44 (1.32 to 1.57)	1.39 (1.27 to 1.52)
2–3	2797	Reference	Reference
>3	1115	1.06 (0.97 to 1.16)	1.05 (0.96 to 1.16)

*Adjusted for age, sex and education.

†Adjusted for age, sex, education, Body Mass Index, smoking, alcohol consumption, marital status, physical exercise and hypertension.

CHD, coronary heart disease; CMD, cardiometabolic disease; HF, heart failure; HS, haemorrhagic stroke; IS, ischaemic stroke; T2DM, type 2 diabetes mellitus.

Compared with non-LBW, the OR for the association between LBW and any CMD was 1.37 (95% CI 1.25 to 1.50). The multiadjusted ORs of LBW were 1.28 (95% CI 1.17 to 1.41) for only one CMD, 1.48 (95% CI 1.28 to 1.72) for any two CMDs and 1.82 (95% CI 1.37 to 2.42) for three or more CMDs (reference: CMD-free), indicating the LBW–CMD risk became higher when multiple CMDs were co-occurring (p for trend<0.001) (online supplemental table S2). Further, the OR of the birth weight–CMD association was 0.84 (95% CI 0.80 to 0.89) when birth weight was used as a continuous variable, suggesting a dose-dependent relationship between greater birth weight and lower CMD risk (online supplemental table S3).

### Association between LBW and CMDs in cotwin matched case–control analysis

In the cotwin matched case–control analysis consisting of 845 dizygotic pairs and 290 monozygotic pairs, the association between LBW and any CMD was attenuated compared with the GEE model and became non-significant (OR 1.21, 95% CI 0.94 to 1.56). The ORs for the associations were 1.34 (95% CI 0.96 to 1.89) in dizygotic pairs and 1.07 (95% CI 0.66 to 1.73) in monozygotic pairs (table 3).

**Table 3:** ORs and 95% CIs for the association between LBW and adult CMDs in cotwin control analysis using CMDs discordant twin pairs: results from conditional logistic regression

Cotwin control	Cotwin with CMDs					
	All zygosity twins* (n=1293 pairs)		Dizygotic only (n=845 pairs)		Monozygotic only (n=290 pairs)	
	Non-LBW	LBW	Non-LBW	LBW	Non-LBW	LBW
Non-LBW	804	177	549	106	162	46
LBW	153	159	90	100	45	37
Basic-adjusted OR (95% CI)†	1.20 (0.96 to 1.49)		1.25 (0.94 to 1.67)		1.03 (0.68 to 1.56)	
Multiadjusted OR (95% CI)‡	1.21 (0.94 to 1.56)		1.34 (0.96 to 1.89)		1.07 (0.66 to 1.73)	

*Contain 158 pairs of undetermined zygosity twins.

†Adjusted for sex and education.

‡Adjusted for sex, education, body mass index, smoking, alcohol consumption, marital status, physical exercise and hypertension.

CMDs, cardiometabolic diseases; LBW, low birth weight.

The difference in ORs from the GEE model versus conditional logistic model was statistically significant (OR 1.39, 95% CI 1.21 to 1.59, p<0.001), which suggested that genetic and early-life environment factors might play an important role in LBW–CMD association.

### Association between lifestyle-related factors and CMDs

In basic-adjusted and multiadjusted GEE models, not smoking, no/moderate alcohol drinking, active physical exercise and being non-overweight were individually related to a decreased risk of any CMD. When combined as a lifestyle index (unfavourable, intermediate and favourable), compared with an unfavourable lifestyle profile, an intermediate and a favourable lifestyle profile were significantly associated with a lower risk of any CMD, ORs were 0.62 (95% CI 0.55 to 0.69) and 0.40 (95% CI 0.35 to 0.47), respectively (table 4).

**Table 4:** ORs and 95% CIs of smoking, alcohol consumption, physical exercise and BMI related to cardiometabolic diseases from generalised estimating equation models

Lifestyle factors	Cases (n)*	OR (95% CI) †	OR (95% CI) ‡
Smoking			
Yes	1886	Reference	Reference
No	1751	0.81 (0.74 to 0.87)	0.80 (0.74 to 0.88)
Alcohol consumption			
Heavy drinking	312	Reference	Reference
No/mild drinking	3325	0.72 (0.62 to 0.83)	0.83 (0.71 to 0.97)
Active physical exercise			
No	1977	Reference	Reference
Yes	1660	0.74 (0.69 to 0.80)	0.85 (0.78 to 0.92)
BMI			
≥25 (overweight)	2109	Reference	Reference
<25 (non-overweight)	1528	0.50 (0.46 to 0.54)	0.59 (0.54 to 0.64)
Lifestyle index (scored 0–4)			
Unfavourable (0–1)	816	Reference	Reference
Intermediate (2-3)	2405	0.57 (0.51 to 0.63)	0.62 (0.55 to 0.69)
Favourable (4)	416	0.34 (0.30 to 0.40)	0.40 (0.35 to 0.47)
P for trend		<0.001	<0.001

*1748 cases before Screening Across the Lifespan Twin study survey were excluded.

†Adjusted for age, sex and education.

‡Adjusted for age, sex, education, marital status, hypertension and birth weight, as well as BMI, smoking, alcohol consumption and active physical exercise, if applicable.

BMI, Body Mass Index.

### Joint effect of LBW and healthy lifestyle factors on CMD risk

**Figure 1:**
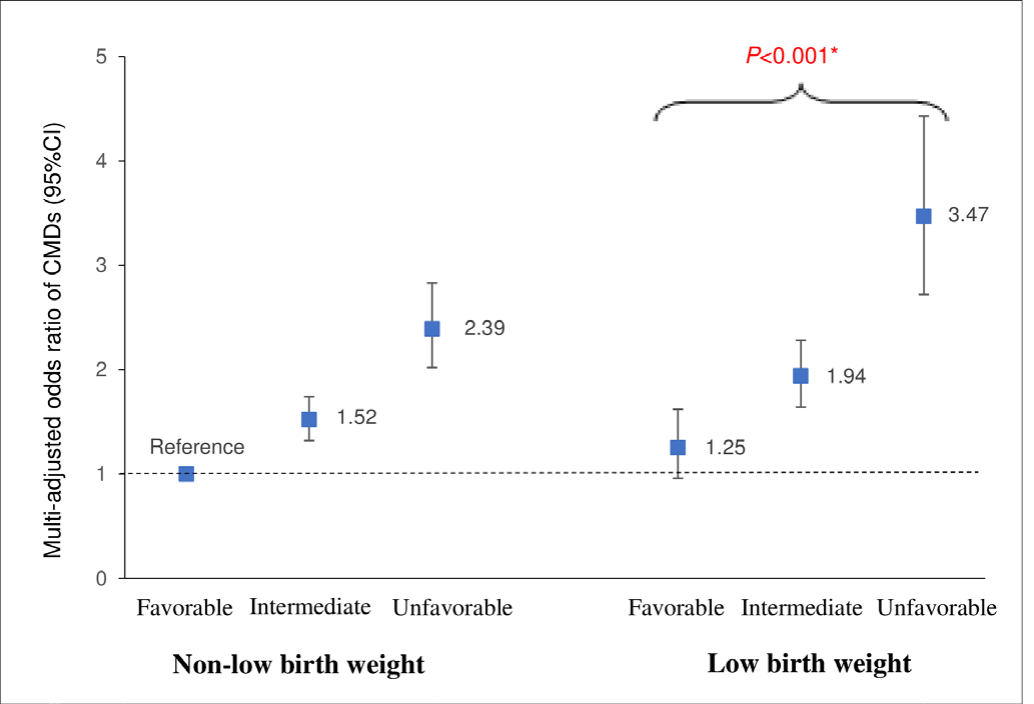
Joint effect of low birth weight (LBW) and lifestyle (smoking status, alcohol consumption, active physical exercise and Body Mass Index) on CMDs. Multiadjusted ORs (95% CI) of CMDs in relation to joint exposure of LBW and lifestyle from generalised estimating equation models (adjusted for age, sex, education, marital status and hypertension). *P value of <0.001 refers to the difference in the risk of CMDs between participants with LBW who have a favourable lifestyle versus those with LBW who have an unfavourable lifestyle. CMDs, cardiometabolic diseases.

In the joint effect analysis, the multiadjusted ORs of any CMD were 1.25 (95% CI 0.96 to 1.62) for participants with LBW plus a favourable lifestyle profile, 1.94 (95% CI 1.64 to 2.28) for those with LBW plus an intermediate lifestyle profile, and 3.47 (95% CI 2.72 to 4.43) for those with LBW plus an unfavourable lifestyle profile (reference: those with non-LBW plus a favourable lifestyle profile) (figure 1 and online supplemental table S4).

The additive interaction between the unfavourable lifestyle profile and LBW on CMDs was statistically significant (AP 0.199, 95% CI 0.016 to 0.381, p=0.03; S 1.506, 95% CI 1.001 to 2.267, p<0.001), indicating that if people with LBW have a favourable or intermediate lifestyle, the risk of LBW on CMDs can be reduced by 20% (online supplemental table S5).

### Supplementary analysis

The results were not much altered compared with those from the initial analysis when we repeated the following analyses after: (1) stratifying by sex to address possible sex differences in the CMDs^38^ (online supplemental table S6), (2) additionally adjusting for survival status considering the association between LBW and mortality^39^ (online supplemental table S7), (3) excluding participants who developed CMDs before SALT recruitment (n=1748) (online supplemental table S8), (4) excluding participants with missing values for covariates (n=1430) (online supplemental table S9), and (5) stratifying by twin birthweight concordance and discordance (online supplemental table S10).

## Discussion

In this large-scale, prospective, population-based nested case–control study of Swedish twins, we found that (1) LBW was associated with an increased risk of CMDs including coronary heart disease, heart failure, ischaemic stroke and T2DM in adulthood, and the risk became higher when multiple CMDs were co-occurring; (2) genetic background and early-life environmental factors appear to account for the LBW–CMD association; (3) a favourable lifestyle profile may modify the risk effect of LBW on CMDs.

Over the past two decades, the relationship between birth weight and T2DM^12 13 40^ has been well documented. However, reports have been inconsistent regarding the association between birth weight and coronary heart disease. Three cohort studies have reported a relationship between LBW and the risk of coronary heart disease.^10 11 41^ By contrast, Banci *et al* found that higher birth weight was associated with a higher risk of coronary heart disease.^14^ Another study showed there was no relationship between birth weight and coronary heart disease.^15^ In addition, evidence on the relationship between LBW and heart failure or ischaemic stroke is sparse. To our knowledge, no studies have investigated the association of LBW with the risk of CMDs. In the present study, we found that LBW was associated with about 10%–40% increased risk of coronary heart disease, heart failure, ischaemic stroke (not haemorrhagic stroke) and T2DM. Further, we examined the relationship between birth weight and the risk of combined CMDs and found that individuals with LBW had an almost 40% higher risk of any CMD compared with those with non-LBW.

The potential contribution of genetic susceptibility and early-life environmental factors to the LBW–CMD association is still unclear. Previous twin cohort studies have shown that LBW is associated with an increased risk of CVD and T2DM when twins were considered as independent individuals. This association only held in outcome-discordant dizygotic twins but not in monozygotic twin pairs, suggesting that genetic mechanisms played a role in this association.^13 32 42^ In the present study, we found that the LBW–CMD association became non-significant in both dizygotic and monozygotic twin pairs by using cotwin matched analyses. These results illustrated that early-life environmental factors could play an important role in the association between LBW and subsequent CMDs, along with genetic background.

Modifiable lifestyle factors (such as smoking, drinking, physical exercise and BMI) deserve to be studied in the context of the LBW–CMD association. To date, only a few studies have investigated the joint effect of LBW with lifestyle factors on T2DM.^27 28 43^ One of the studies included 149 794 participants from three large prospective cohorts and showed that LBW and unhealthy adulthood lifestyles encompassing smoking, non-moderate alcohol consumption, lower exercise intensity and BMI≥25 were jointly related to an increased risk of T2DM.^28^ Another cohort study indicated that the risk of diabetes associated with LBW could be eliminated in those with a high physical activity level,^27^ and individuals predisposed to T2DM due to LBW could be protected from glucose intolerance by regular exercise.^43^ However, no study has illustrated the joint effect of LBW and healthy lifestyle on subsequent CMDs. In the present study, we found that people with LBW and an intermediate or a favourable lifestyle profile (including not smoking, no/mild alcohol consumption, active physical exercise and being non-overweight) had a significantly lower risk of CMDs than those who had LBW and unfavourable lifestyle profile. To our knowledge, this is the first study to provide evidence that a healthy lifestyle might compensate for the risk effect of LBW on CMDs.

Several mechanisms may explain the relationship between LBW and the risk of CMDs. Twins have a unique and highly distinctive pattern of fetal growth. Although there is a higher rate of preterm birth among twins^44^ who may have lower birth weight compared with single births, a preterm fetus with LBW may have appropriate fetal growth. Actual growth restriction could occur when twins fail to adapt to an intrauterine environment. Foetal malnutrition or inappropriate growth in gestation may redirect scant energy supplies from muscle to vital tissues, causing permanent alterations in physiology, metabolism and structure.^45 46^ Nevertheless, LBW alone could not fully capture the true growth level of the fetus, and monitoring the entire period of twin pregnancy is necessary to clarify the mechanism between LBW and CMDs in twins. Additionally, some genes (such as insulin class I allele or variants of mitochondrial DNA) have been associated with both birth weight loss and insulin resistance.^47 48^ All of these alterations could result in an increased risk of CVD and T2DM in adulthood. Moreover, a haplotype of the glucocorticoid receptor gene may modify the association between size at birth and glucose tolerance.^49^ However, maintaining a healthy lifestyle in adulthood may mitigate the risk of CMDs by improving insulin sensitivity and body composition, as well as controlling glycaemic, blood pressure and lipid profile.^50^

### Strengths and limitations

Notable strengths of our study involve the large nationwide population-based twin cohort, which provided an extraordinary opportunity to explore the association between LBW and the risk of CMDs in adulthood by controlling for some unmeasured confounders, such as genetic background and early-life environmental factors. Furthermore, our investigation of potential compensatory factors against the LBW–CMD association is unique. Nevertheless, some limitations should be pointed out. First, hypertension was defined only based on self-reported data from the NPR, and subjects with undiagnosed hypertension might have been misclassified as hypertension-free. Thus, hypertension was not categorised as a CMD in the current study. Second, the assessment of birth weight was based on self-report so potential information bias could not be ruled out. However, such bias is more likely to be non-differential misclassification resulting in underestimation for the given associations. Third, data on gestational age and other prenatal factors (such as maternal smoking during pregnancy, premature birth or parental socioeconomic status) were not available and could not be fully controlled for. In addition, potential variations in lifestyle factors during follow-up could not be assessed. Fourth, diet could be partially taken into account, as it is closely associated with other lifestyle factors such as smoking, alcohol consumption, physical exercise and BMI.^51^ However, data on diet were not available in the SALT study. Finally, LBW in this study was defined as <2.0 kg in twins. Caution is needed when generalising our findings to other populations.

## Conclusion

This study provides evidence that LBW is associated with increased risk of CMDs including coronary heart disease, heart failure, ischaemic stroke and T2DM. The risk of CMDs related to LBW tends to increase with the number of co-occurring CMDs. Further, genetic and early-life environmental factors play an important role in the LBW–CMD association. However, a favourable lifestyle involving not smoking, no/mild alcohol consumption, active physical exercise and a BMI of <25 may compensate for the risk effect of LBW on CMDs. Our findings highlight the need for monitoring and controlling LBW for the prevention of CMDs, and the importance of maintaining a favourable lifestyle profile in people with LBW in adulthood to reduce the risk of CMDs.

## Supplementary Material

Reviewer comments

Author's manuscript

## Data Availability

Data are available upon reasonable request.
