# Supplementary material for: Association of low birth weight with cardiometabolic diseases in Swedish twins: a population-based cohort study
Source: BMJ Open. 2021 Jun 28;11(6):e048030. doi: 10.1136/bmjopen-2020-048030 (PMC8240562; doi:10.1136/bmjopen-2020-048030)
Supplement: Supplementary data [file bmjopen-2020-048030supp001.pdf]

Supplemental Materials

Including: Tables-10; Figure-1

Table S1. International Classification of Disease (ICD) code of cardiometabolic diseases

Table S2. The relationship between low birth weight and numbers of cardiometabolic diseases (CMDs): results from Generalized Estimating Equation

Table S3. The dose-dependent relationship between low birth weight and cardiometabolic disease: results from Generalized Estimating Equation

Table S4. Odds ratios (ORs) and 95% confidence intervals (CIs) of cardiometabolic diseases in relation to the joint exposure of lifestyle (smoking status, alcohol consumption, active physical exercise, and body mass index) and low birth weight (LBW) from Generalized Estimation Equation models

Table S5. Additive interaction between lifestyle (smoking status, alcohol consumption, active physical exercise, and body mass index) and low birth weight (LBW) for the risk of cardiometabolic diseases

Table S6. Odds ratios (ORs) and 95% confidence intervals (CIs) of birth weight in relation to CMDs by sex: results from Generalized Estimating Equation

Table S7. Odds ratios (ORs) and 95% confidence intervals (CIs) of birth weight in relation to CMDs in adulthood further adjusted for survival status: results from Generalized Estimating Equation models

Table S8. Odds ratios (ORs) and 95% confidence intervals (CIs) of birth weight in relation to cardiometabolic diseases in adulthood by excluding cardiometabolic diseases onset before screening: results from Generalized Estimating Equation (n=18301)

Table S9. Odds ratios (ORs) and 95% confidence intervals (CIs) of birth weight in relation to cardiometabolic diseases in adulthood by excluding data with missing values for covariate: results from Generalized Estimating Equation (n=18349)

Table S10. Odds ratios (ORs) and 95% confidence intervals (CIs) of low birth weight (LBW) in relation to cardiometabolic diseases in adulthood stratified by consistency of birth weight: results from Generalized Estimating Equation

Figure S1. Flow chart of the study population

Table S1. International Classification of Disease (ICD) code of cardiometabolic diseases

Cardiometabolic diseases	ICD-7	ICD-8	ICD-9	ICD-10
Coronary heart disease	420	410-414	410-414	I20-I25
Heart failure	434	427	428	I50
Ischemic stroke	332-334	432-438	433-437	I63-I68, G47
Hemorrhagic stroke	330-331	430-431	430-432	I60-I62
Type 2 diabetes mellitus	260	250	250	E11-E14

Li X, *et al. BMJ Open* 2021; 11:e048030. doi: 10.1136/bmjopen-2020-048030

Table S2. The relationship between low birth weight and numbers of cardiometabolic diseases (CMDs): results from Generalized Estimating Equation

CMDs status	No. of participants	Low birth weight		
		No. of cases	Basic-adjusted OR (95% CI) *	Multi-adjusted OR (95% CI) †
No	14444	2575	Reference	Reference
Any one	5335	1423	1.43 (1.31-1.55)	1.37 (1.25-1.50)
Only one	3932	989	1.32 (1.21-1.45)	1.28 (1.17-1.41)
Any two	1174	355	1.56 (1.36-1.80)	1.48 (1.28-1.72)
Any three or more	229	79	1.94 (1.47-2.56)	1.82 (1.37-2.42)
P for trend			<0.001	<0.001

* Adjusted for age, sex, and education.

† Adjusted for age, sex, education, body mass index, smoking, alcohol consumption, marital status, physical exercise, and hypertension.

Li X, *et al. BMJ Open* 2021; 11:e048030. doi: 10.1136/bmjopen-2020-048030

Table S3. The dose-dependent relationship between low birth weight and cardiometabolic disease: results from Generalized Estimating Equation

Birth weight	No. of Case	Basic-adjusted OR (95% CI) *	Multi-adjusted OR (95% CI) †
Continuous		0.83 (0.79-0.88)	0.84 (0.80-0.89)
Categorical			
<1.7	622	1.54 (1.36-1.74)	1.45 (1.28-1.66)
1.7-2.0kg	801	1.35 (1.22-1.49)	1.32 (1.18-1.47)
≥2.0kg	3912	Reference	Reference
P for trend		<0.001	<0.001

* Adjusted for age, sex, and education.

† Adjusted for age, sex, education, body mass index, smoking, alcohol consumption, marital status, physical exercise, and hypertension.

Li X, *et al. BMJ Open* 2021; 11:e048030. doi: 10.1136/bmjopen-2020-048030

Table S4. Odds ratios (ORs) and 95% confidence intervals (CIs) of cardiometabolic diseases in relation to the joint exposure of lifestyle (smoking status, alcohol consumption, active physical exercise, and body mass index) and low birth weight (LBW) from Generalized Estimation Equation models

Joint exposure		No. of subjects *	Cardiometabolic diseases		
Lifestyle index	LBW		Cases	Basic-adjusted OR (95% CI) †	Multi-adjusted OR (95% CI) ‡
Favorable	No	2533	314	Reference	Reference
Intermediate	No	9751	1795	1.65 (1.44-1.87)	1.52 (1.32-1.74)
Unfavorable	No	2274	620	2.90 (2.47-3.40)	2.39 (2.02-2.83)
Favorable	Yes	570	102	1.32 (1.03-1.70)	1.25 (0.96-1.62)
Intermediate	Yes	2362	610	2.18 (1.86-2.54)	1.94 (1.64-2.28)
Unfavorable	Yes	541	196	3.89 (3.08-4.90)	3.47 (2.72-4.43)

* 1748 cases before Screening Across the Lifespan Twin study survey were exclude.

† Adjusted for age, sex, education.

‡ Adjusted for age, sex, education, marital status, and hypertension.

Li X, *et al. BMJ Open* 2021; 11:e048030. doi: 10.1136/bmjopen-2020-048030

Table S5. Additive interaction between lifestyle (smoking status, alcohol consumption, active physical exercise, and body mass index) and low birth weight (LBW) for the risk of cardiometabolic diseases

Joint exposure		No. of subjects *	Cardiometabolic diseases		
Lifestyle index	LBW		Cases	Basic-adjusted OR (95% CI) †	Multi-adjusted OR (95% CI) ‡
Favorable/Intermediate	No	12284	2109	Reference	Reference
Unfavorable	No	2274	620	1.91 (1.70-2.14)	1.68 (1.49-1.90)
Favorable/Intermediate	Yes	2932	712	1.33 (1.20-1.47)	1.28 (1.14-1.42)
Unfavorable	Yes	541	196	2.56 (2.09-3.15)	2.44 (1.97-3.03)

* 1748 cases before Screening Across the Lifespan Twin study survey were exclude.

† Adjusted for age, sex, education.

‡ Adjusted for age, sex, education, marital status, and hypertension.

Measures of additive interaction for cardiometabolic diseases:

Relative excess risk due to interaction: 0.485, 95%CI: -0.044–1.014, *P*=0.07;

Attributable proportion due to interaction: 0.199, 95%CI: 0.016–0.381, *P*=0.03;

Synergy index: 1.506, 95% CI: 1.001–2.267, *P*<0.001.

Li X, *et al. BMJ Open* 2021; 11:e048030. doi: 10.1136/bmjopen-2020-048030

Table S6. Odds ratios (ORs) and 95% confidence intervals (CIs) of birth weight in relation to CMDs by sex: results from Generalized Estimating Equation

Birth weight (kg)	No. of Cases	OR (95% CI) *	OR (95% CI) †
Male			
<2.0	564	1.39 (1.20-1.61)	1.44 (1.23-1.69)
2.0-3.0	1050	Reference	Reference
>3.0	642	1.06 (0.93-1.21)	1.07 (0.93-1.23)
Female			
<2.0	859	1.47 (1.32-1.63)	1.36 (1.21-1.52)
2.0-3.0	1747	Reference	Reference
>3.0	473	1.06 (0.94-1.19)	1.04 (0.91-1.19)

* Adjusted for age, sex, and education.

† Adjusted for age, sex, education, body mass index, smoking, alcohol consumption, marital status, physical exercise, and hypertension.

Li X, *et al. BMJ Open* 2021; 11:e048030. doi: 10.1136/bmjopen-2020-048030

Table S7. Odds ratios (ORs) and 95% confidence intervals (CIs) of birth weight in relation to CMDs in adulthood further adjusted for survival status: results from Generalized Estimating Equation models

Birth weight (kg)	No. of Cases	OR (95% CI) *
<2.0	1423	1.38 (1.26-1.52)
2.0-3.0	2797	Reference
>3.0	1115	1.05 (0.95-1.16)

* Adjusted for age, sex, education, body mass index, smoking, alcohol consumption, marital status, physical exercise, hypertension, and death.

Li X, *et al. BMJ Open* 2021; 11:e048030. doi: 10.1136/bmjopen-2020-048030

Table S8. Odds ratios (ORs) and 95% confidence intervals (CIs) of birth weight in relation to cardiometabolic diseases in adulthood by excluding cardiometabolic diseases onset before screening: results from Generalized Estimating Equation (n=18301)

Birth weight (kg)	No. of Cases	OR (95% CI) *	OR (95% CI) †
<2.0	908	1.34 (1.22-1.48)	1.30 (1.17-1.45)
2.0-3.0	1969	Reference	Reference
>3.0	760	1.03 (0.93-1.13)	1.02 (0.92-1.14)

* Adjusted for age, sex, and education.

† Adjusted for age, sex, education, body mass index, smoking, alcohol consumption, marital status, physical exercise, and hypertension.

Li X, *et al. BMJ Open* 2021; 11:e048030. doi: 10.1136/bmjopen-2020-048030

Table S9. Odds ratios (ORs) and 95% confidence intervals (CIs) of birth weight in relation to cardiometabolic diseases in adulthood by excluding data with missing values for covariate: results from Generalized Estimating Equation (n=18349)

Birth weight (kg)	No. of Cases	OR (95% CI) *	OR (95% CI) †
<2.0	1184	1.49 (1.36-1.63)	1.43 (1.30-1.58)
2.0-3.0	2359	Reference	Reference
>3.0	937	1.05 (0.96-1.15)	1.03 (0.93-1.14)

* Adjusted for age, sex, and education.

† Adjusted for age, sex, education, body mass index, smoking, alcohol consumption, marital status, physical exercise, and hypertension.

Li X, *et al. BMJ Open* 2021; 11:e048030. doi: 10.1136/bmjopen-2020-048030

Table S10. Odds ratios (ORs) and 95% confidence intervals (CIs) of low birth weight (LBW) in relation to cardiometabolic diseases in adulthood stratified by consistency of birth weight: results from Generalized Estimating Equation

Birth weight (kg)	No. of Cases	OR (95% CI) *	OR (95% CI) †
Concordance			
LBW	347	1.53 (1.29-1.82)	1.47 (1.23-1.76)
Non-LBW	1370	Reference	Reference
Discordance			
LBW	334	1.16 (0.97-1.39)	1.13 (0.93-1.39)
Non-LBW	310	Reference	Reference

* Adjusted for age, sex, and education.

† Adjusted for age, sex, education, body mass index, smoking, alcohol consumption, marital status, physical exercise, and hypertension.

Li X, *et al. BMJ Open* 2021; 11:e048030. doi: 10.1136/bmjopen-2020-048030

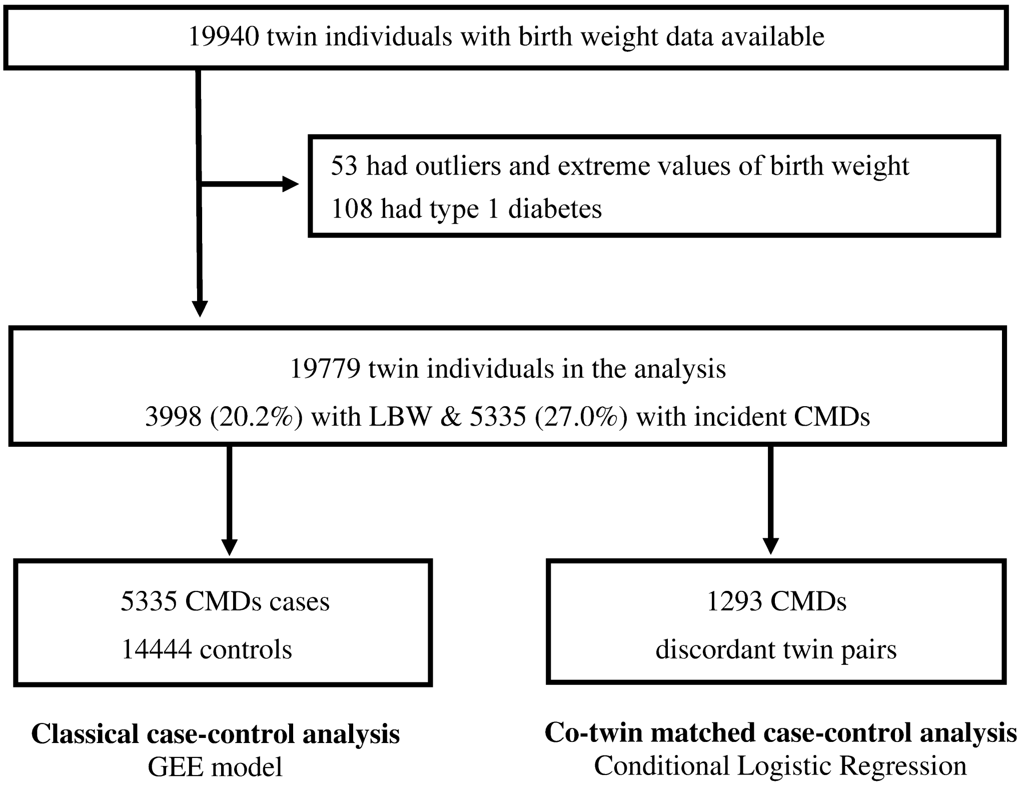

Figure S1. Flow chart of the study population

Abbreviations: LBW, low birth weight; CMDs, cardiometabolic diseases; GEE, generalized estimating equation.
