# Supplementary material for: Association of low birth weight with cardiometabolic diseases in Swedish twins: a population-based cohort study
Source: BMJ Open. 2021 Jun 28;11(6):e048030. doi: 10.1136/bmjopen-2020-048030 (PMC8240562; doi:10.1136/bmjopen-2020-048030)
Supplement: Author's manuscript [file bmjopen-2020-048030.draft_revisions.pdf]

BMJ Open

BMJ Open is committed to open peer review. As part of this commitment we make the peer review history of every article we publish publicly available.

When an article is published we post the peer reviewers' comments and the authors' responses online. We also post the versions of the paper that were used during peer review. These are the versions that the peer review comments apply to.

The versions of the paper that follow are the versions that were submitted during the peer review process. They are not the versions of record or the final published versions. They should not be cited or distributed as the published version of this manuscript.

BMJ Open is an open access journal and the full, final, typeset and author-corrected version of record of the manuscript is available on our site with no access controls, subscription charges or pay-per-view fees (http://bmjopen.bmj.com).

If you have any questions on BMJ Open's open peer review process please email info.bmjopen@bmj.com

BMJ Open

Association of low birth weight with cardiometabolic diseases in the Swedish twins: the role of healthy lifestyle

Journal:	*BMJ Open*
Manuscript ID	bmjopen-2020-048030
Article Type:	Original research
Date Submitted by the Author:	23-Dec-2020
Complete List of Authors:	Li, Xuerui; Tianjin Medical University, Department of Epidemiology and Biostatistics, School of Public Health<br>Yang, Rongrong; Tianjin Medical University, Department of Epidemiology and Biostatistics, School of Public Health<br>Yang, Wenzhe; Tianjin Medical University, Department of Epidemiology and Biostatistics, School of Public Health<br>Xu, Hui; Tianjin Medical University, Department of Epidemiology and Biostatistics, School of Public Health<br>Song, Ruixue; Tianjin Medical University, Department of Epidemiology and Biostatistics, School of Public Health<br>Qi, Xiuying; Tianjin Medical University, Department of Epidemiology and Biostatistics, School of Public Health<br>Xu, Weili; Karolinska Institutet, Aging Research Center, Department of Neurobiology, Health Care Sciences and Society; Tianjin Medical University, Department of Epidemiology and Biostatistics, School of Public Health
Keywords:	EPIDEMIOLOGY, PUBLIC HEALTH, Adult cardiology < CARDIOLOGY, General diabetes < DIABETES & ENDOCRINOLOGY

SCHOLARONE™
Manuscripts

I, the Submitting Author has the right to grant and does grant on behalf of all authors of the Work (as defined in the below author licence), an exclusive licence and/or a non-exclusive licence for contributions from authors who are: i) UK Crown employees; ii) where BMJ has agreed a CC-BY licence shall apply, and/or iii) in accordance with the terms applicable for US Federal Government officers or employees acting as part of their official duties; on a worldwide, perpetual, irrevocable, royalty-free basis to BMJ Publishing Group Ltd ("BMJ") its licensees and where the relevant Journal is co-owned by BMJ to the co-owners of the Journal, to publish the Work in this journal and any other BMJ products and to exploit all rights, as set out in our licence.

The Submitting Author accepts and understands that any supply made under these terms is made by BMJ to the Submitting Author unless you are acting as an employee on behalf of your employer or a postgraduate student of an affiliated institution which is paying any applicable article publishing charge ("APC") for Open Access articles. Where the Submitting Author wishes to make the Work available on an Open Access basis (and intends to pay the relevant APC), the terms of reuse of such Open Access shall be governed by a Creative Commons licence – details of these licences and which Creative Commons licence will apply to this Work are set out in our licence referred to above.

Other than as permitted in any relevant BMJ Author's Self Archiving Policies, I confirm this Work has not been accepted for publication elsewhere, is not being considered for publication elsewhere and does not duplicate material already published. I confirm all authors consent to publication of this Work and authorise the granting of this licence.

For peer review only

Association of low birth weight with cardiometabolic diseases in the Swedish twins: the role of healthy lifestyle

Xuerui Li[1,2,3], MSc; Rongrong Yang[1,2,3], PhD; Wenzhe Yang[1,2,3], Bs; Hui Xu[1,2,3], PhD; Ruixue Song[1,2,3], MSc; Xiuying Qi[1,2,3]*, PhD; Weili Xu[1,2,3,4]*, PhD

Affiliations of authors:

[1] Department of Epidemiology and Biostatistics, School of Public Health, Tianjin Medical University, Tianjin, China

[2] Tianjin Key Laboratory of Environment, Nutrition and Public Health, Tianjin, China

[3] Center for International Collaborative Research on Environment, Nutrition and Public Health, Tianjin, China

[4] Aging Research Center, Department of Neurobiology, Health Care Sciences and Society, Karolinska Institutet, Stockholm, Sweden

***Corresponding authors**

Weili Xu & Xiuying Qi, Professors

Dept. of Epidemiology & Biostatistics, School of Public Health

Tianjin Medical University, Tianjin, China

Qixiangtai Road 22, Heping district, 300070, Tianjin, PR, China

Fax number: +86 022-83336727; Phone: +86 022-83336727

Email: xuweili@tmu.edu.cn; qixiuying@tmu.edu.cn

Weili Xu, Associate Professor & Senior Researcher

Aging Research Center, Karolinska Institutet

Tomtebodavägen 18A Floor 10, SE-171 65 Solna, Stockholm, Sweden

Phone: +46 8 524 858 26; Email: weili.xu@ki.se

Word count: Title-97 (no space); Abstract-299; Text only-3374; References-46; Tables-4; Figure-1; Supplementary Tables-8; Supplementary Figure-1.

For peer review only - http://bmjopen.bmj.com/site/about/guidelines.xhtml

Abstract

Objective: To examine the association between LBW and CMDs (including heart disease, stroke and T2DM) in adulthood, and to explore whether genetic, early-life environmental and healthy lifestyle factors play a role in this association.

Design: A prospective population-based nested case-control study of Swedish twins.

Setting: Twins from the Swedish Twin Registry who were born in 1958 or earlier participated in the Screening Across the Lifespan Twin study (SALT) for a full-scale screening during 1998-2002 and were followed up till 2014.

Participants: Of the 19940 twin individuals with birth weight available, after excluding 53 individuals who had outliers and 108 who had type 1 diabetes, 19779 individuals remained for the current analyses.

Primary and secondary outcome measures: CMDs were assessed based on self-reported medical record, medication use, and the National Patient Registry. Lifestyle index encompassing smoking, alcohol consumption, physical exercise, and body mass index was assessed from SALT survey and categorized as unfavorable, intermediate, or favorable. Data was analyzed using generalized estimating equation (GEE) models and conditional logistic regression models.

Results: Of all participants, 3998 (20.2%) had LBW and 5335 (27.0%) had incident CMDs (mean age at onset: 63.64±13.26). In GEE models, the odds ratio, 95% confidence interval (OR, 95% CI) of LBW was 1.39 (1.27-1.52) for any CMD. In conditional logistic regression models, the LBW-CMDs association became non-significant (OR 1.21, 95% CI 0.94-1.56). The differences in ORs from the two models were statistically significant (P<0.001). In joint

For peer review only - http://bmjopen.bmj.com/site/about/guidelines.xhtml

For peer review only

effect analysis, the multi-adjusted OR (95% CI) of CMDs was 3.47 (2.72-4.43) for participants with LBW plus an unfavorable lifestyle and 1.25 (0.96-1.62) for those with LBW plus a favorable Lifestyle.

Conclusions: LBW is associated with an increased risk of adult CMDs, and genetic and early-life environmental factors may account for this association. However, a favorable lifestyle profile may modify this risk.

Key words: Population-based twin study; Birth weight; Cardiometabolic disease; the Swedish twins; Lifestyle

Strengths and limitations of this study:

This study provides an extraordinary opportunity to explore the LBW-CMD association by controlling for some unmeasured confounders, such as genetic background and early-life environmental factors.

This study on compensatory factors for the risk effect of LBW on CMDs is unique.

Birth weight were based on self-reports and non-differential misclassification among different birth weights groups could not be ruled out, possibly leading to an underestimation of the observed associations.

Some prenatal factors (such as maternal smoking during pregnancy or premature birth) could not be controlled for, as information on these factors were not available.

Potential variations of lifestyle factors during follow-up could not be assessed.

For peer review only - http://bmjopen.bmj.com/site/about/guidelines.xhtml

Introduction

With population ageing, chronic diseases are becoming more common, especially heart diseases (i.e. coronary heart diseases and heart failure), stroke and type 2 diabetes mellitus (T2DM).[1] According to the World Health Organization (WHO), heart diseases and stroked, so called cardiovascular disease (CVD), is the leading cause of disease burden and death across the world.[2,3] About 17.6 million deaths were attributed to CVD globally in 2016.[2] Meanwhile, the global prevalence of diabetes has risen from 4.7% to 8.5% from 1980 till 2014 in adult population.[4] All of these co-occurring chronic diseases above have been defined as cardiometabolic diseases (CMDs).[5,6]

Recently, beyond the effects of some traditional risk factors including age, smoking, drinking, and body mass index (BMI) on individual CMDs, the role of early-life experiences in future development of chronic diseases have drawn special attention.[7] Birth weight, an early life indicator and a proxy for fetal growth trajectory,[8] is frequently used to explore the effects of early-life experiences on the risk of individual CMDs in adulthood. Several cohort studies have shown that low birth weight (LBW) was associated with an increased risk of coronary heart disease[9] stroke[10] or T2DM,[11,12] but with some inconsistent findings.[13,14] So far, no studies have investigated the association of LBW with the risk of combined CMDs.

CMDs is a complex genetic and lifestyle-related disorder,[15-17] and birth weight may also be affected by genetic factors and intrauterine environments.[18] However, the role of the genetic and early-life environmental factors (i.e. intrauterine environment and prenatal nutritional status) in the association between birth weight and CMDs remains unclear. Twin studies could make it possible to minimize potential confounding effects of unmeasured genetic predisposition and shared early-life environment when comparisons are made between twins.[19,20] Apart from genetic factors, some modifiable lifestyle factors such as non-smoking, moderate alcohol consumption, physical activities, and maintaining a healthy weight have

For peer review only - http://bmjopen.bmj.com/site/about/guidelines.xhtml

been reported linking to a lower risk of CVD or T2DM.[21,22] However, previous population-based cohort studies have only shown that healthy lifestyle (such as active physical activity, no smoking, moderate alcohol consumption, and BMI<25) may reduce the risk effects of LBW on the development of diabetes,[23,24] but not involved with CMDs. Questions remain regarding whether and to what extent healthy lifestyle may mitigate the risk of LBW on CMDs.

In the present study, we sought to 1) examine the associations between LBW and risk of CMDs in adulthood, 2) explore whether the genetic and early-life environment factors could explain the LBW-CMDs association, and 3) assess whether healthy lifestyle could compensate for the risk of LBW on CMDs using data from the population-based Swedish twin cohort.

For peer review only

Methods

Study population

This prospective, nested case-control study included twins from the nationwide Swedish Twin Registry (STR), which started in the 1960s.[25] During 1998-2002, all living twins born in 1958 or earlier were recruit to participate in the Screening Across the Lifespan Twin study (SALT), a full-scale screening through a computer-assisted telephone interview. Of the 19940 twin individuals with birth weight available, we excluded 53 individuals who had outliers (extreme values) of birth weight (i.e. birth weight ≤300 g or ≥4520g) and 108 who had type 1 diabetes. Finally, 19779 individuals were included in the current study (Supplemental Figure S1).

Data collection

Data on age, sex, educational attainment, marital status, and zygosity status were collected through the SALT survey.[25] Zygosity status was categorized as monozygotic, dizygotic, and undetermined zygosity. Education was defined according to the number of years of formal

For peer review only - http://bmjopen.bmj.com/site/about/guidelines.xhtml

schooling attained and dichotomized into <8 vs. ≥8 years. Marital status was classified into married/cohabitating vs. single (including divorced or widows/widowers).

Information on medical history including heart disease, stroke, T2DM and hypertension was derived from the National Patient Registry (NPR), which covers all inpatient diagnoses in Sweden from the 1960s and outpatient (specialist clinic) diagnoses from 2001 till 2014.[26] Each medical record in the NPR included up to eight discharge diagnoses according to the International Classification of Disease (ICD) codes. The seventh revision (ICD-7) was used through 1968, the eighth revision (ICD-8) from 1969 to 1986, the ninth revision (ICD-9) from 1987 till 1996, and the tenth revision (ICD-10) from 1997 through the end of 2014.

Informed consent was required from all participants. Data collection procedures were approved by the Regional Ethics Committee at Karolinska Institutet, Stockholm, Sweden and the Institutional Review Board of the University of Southern California, USA.

Assessment of birth weight

Data on birth weight were collected based on self-reports from SALT or STR. Generally, LBW was defined as birth weight <2500g in singletons.[27] However, twins may experience a more unfavorable intrauterine environment, causing them to have a lower birth weight (on average 800g) than singletons.[28] Thus, birth weight in the present study was categorized as <2.0 kg (LBW), 2.0-3.0 kg (moderate birth weight [MBW]), or >3.0kg (high birth weight [HBW])[28] considering its distribution.

Ascertainment of CMD

In the current analysis, CMDs included heart disease, stroke, and T2DM, all of which were diagnosed based on self-reported medical record, medication use, and NPR data. Heart diseases included coronary heart disease (ICD-7 codes 420, ICD-8 and -9 codes 410-414, ICD-10 codes I20-I25) and heart failure (ICD-7 codes 434, ICD-8 codes 427, ICD-9 codes 428, ICD-10 codes I50). Stroke encompassed ischemic stroke (ICD-7 codes 332-334, ICD-8

For peer review only - http://bmjopen.bmj.com/site/about/guidelines.xhtml

codes 432-438, ICD-9 codes 433-437, ICD-10 codes I63-I68, G47) and hemorrhagic stroke (ICD-7 codes 330-331, ICD-8 codes 430-431, ICD-9 codes 430-432, ICD-10 codes I60-I62). T2DM diagnosis in NPR was ascertained based on codes of ICD-7 260, ICD-8 and -9 250, and ICD-10 E11-E14.

CMDs status was categorized as CMD-free and any CMD (suffering from any one of the following diseases: heart disease, stroke, and T2DM). Any CMD was further classified as: only one CMD (heart disease, or stroke, or T2DM), any two CMDs (any two of the following: heart disease, stroke, or T2DM), and three or more CMDs (heart disease, stroke, and T2DM).

Assessment of lifestyle-related factors

Information on lifestyle factors (smoking, alcohol consumption, physical exercise and BMI) was obtained from the SALT survey. In detail, smoking status was dichotomized as non-smoking vs. former/current smoker. Alcohol consumption was grouped into no/mild drinking vs. heavy drinking based on the question about whether participants have ever drunk excessively over a period. Data on physical exercise was collected by a question on average exercise with seven response options: I) “almost never,” II) “much less than average,” III) “less than average,” IV) “average,” V) “more than average,” VI) “much more than average,” and VII) “maximum”,[29] and was dichotomized as “inactive” including the first four groups (I-IV) and “active” including last three groups (V-VII). BMI in adulthood (mean age 55.45±9.05) was calculated as weight (kg) divided by squared height (m^2), and classified as underweight (<18.5), normal weight (18.5-24.9), overweight (25-29.9), and obesity (≥30) according to the WHO classification. Obesity was merged with overweight (hereafter overweight; that is, BMI ≥25), and underweight was merged with normal weight as non-overweight (BMI <25).

In the current study, on the basis of the data availability, the following four factors were considered as healthy lifestyle factors: 1) non-smoking; 2) no/mild alcohol consumption; 3)

For peer review only - http://bmjopen.bmj.com/site/about/guidelines.xhtml

active physical exercise; and 4) non-overweight in adult age.[30] The four factors were combined into a lifestyle index with a score ranging from 0-4, with 1 point representing each factor. Participants were categorized according to their score of lifestyle index: 1) unfavorable (0-1): participants who had no healthy lifestyle factors or only one; 2) intermediate (2-3): those who had two or three healthy lifestyle factors; 3) favorable (4): those who had all the healthy lifestyle factors.

Statistical analyses

The characteristics of participants in different groups were compared using Chi-square tests for categorical variables and one-way analysis of variance/Kruskal-Wallis H test for continuous variables. Missing values on education (n=92), smoking (n=77), alcohol consumption (n=117), marital status (n=2), physical exercise (n=1179) and BMI (n=290) were imputed using Rubin's rule for pooling estimates to obtain valid statistical inferences.[20]

Generalized estimating equation (GEE) models were used for unmatched case-control analyses to control for the clustering of twins within a pair. Conditional logistic regression models were used for the co-twin matched case-control study, in twin pairs who were discordant for the outcome. Using twin pairs (especially monozygotic twins) with discordant outcome has been found to be more informative than using unrelated case-control samples, since discordant twins are matched for genetic background and early-life environmental factors such as fetal environment and prenatal nutritional status.[31,32] In both GEE and conditional logistic regression, the odds ratios (ORs) and 95% confidence intervals (CIs) were estimated for the association between birth weight (reference: MBW) and CMDs. Logistic regression was used to test the difference in ORs from GEE and conditional logistic regression models by examining the difference in the proportions of birth weight between unmatched controls and co-twin matched controls.[32] If an OR for the observed association becomes strengthened or attenuated (or even disappears) in co-twin control analyses

For peer review only - http://bmjopen.bmj.com/site/about/guidelines.xhtml

compared with that in the unmatched case-control analysis, and the difference in ORs from the two models is significant, genetic and/or early-life environmental factors are likely to play a role in the association.[20,31,33] Otherwise the effect could be neglected if the OR is similar in two models without statistically significant difference.[19,32]

Considering information on lifestyle factors was obtained during 1998-2002, we excluded 1748 participants with CMDs before SALT recruitment, thus 18031 individuals were remained to perform the joint effect analysis. The combined effect of the LBW (no vs. yes) and lifestyle index (unfavourable/intermediate/favourable) on the risk of CMDs was assessed by creating dummy variables based on the joint exposures to both factors. The presence of additive interaction was examined by estimating relative excess risk due to interaction (RERI), the attributable proportion (AP), and the synergy index (S).

All the models were basic adjusted for age, sex and education, and further adjusted for smoking, alcohol consumption, marital status, physical exercise, BMI, and hypertension. The level of statistical significance was set at a *P*-value less than 0.05. All statistical analyses were performed using SAS statistical software version 9.4 (SAS institute, Cary, NC) and IBM SPSS Statistics 20.0 (IBM Corp, New York, NY).

Patient and public involvement

Patients and the public were not involved in the design, or conduct, or reporting of this study.

Results

Characteristics of the study population

Among all participants (n=19779), 3998 (20.2%) had LBW. The average age at recruitment was 55.45 (±9.05) years. Compared with MBW individuals, those with LBW were more likely to be older, male, monozygotic twins, single, have lower education, have higher BMI, be physically inactive, and have hypertension. Participants who had HBW were more likely to

For peer review only - http://bmjopen.bmj.com/site/about/guidelines.xhtml

be male, dizygotic twins, smokers, heavy drinkers, and have higher BMI (Table 1).

(Insert Table 1 here)

Association between birth weight and CMDs in unmatched case-control analysis

In the multi-adjusted GEE model, compared to participants with MBW, those with LBW had significantly higher risk of coronary heart disease, heart failure, ischemic stroke, and T2DM, which were further combined as CMDs (n=5335), as showed in Table 2. LBW was associated with an increased risk of any CMD (OR 1.39, 95% CI 1.27-1.52). However, HBW was not significantly associated with CMDs (OR 1.05, 95% CI: 0.96-1.16). Therefore, MBW and HBW were combined into non-LBW group as reference in the following analysis.

(Insert Table 2 here)

Compared to non-LBW, the OR for the association between LBW and any CMD was 1.37 (95% CI 1.25-1.50). The multi-adjusted ORs (95% CIs) of LBW were 1.28 (1.17-1.41) for only one CMD, 1.48 (1.28-1.72) for any two CMDs, and 1.82 (1.37-2.42) for three or more CMDs (reference: CMD-free), indicating the LBW-CMDs risk became higher when multiple CMDs were co-occurring (P for trend <0.001) (Supplemental Table S1). Further, the OR of the birth weight-CMDs association was 0.84 (95% CI 0.80-0.89) when birth weight was used as a continuous variable, suggesting the does-dependent relationship between greater birth weight and lower CMDs risk (Supplemental Table S2).

Association between LBW and CMDs in co-twin matched case-control analysis

In the co-twin matched case-control analysis consisting of 845 dizygotic pairs and 290 monozygotic pairs, the association between LBW and any CMD was attenuated and became non-significant (OR: 1.21, 95% CI 0.94-1.56). The ORs (95% CI) for the association were 1.34 (0.96-1.89) in dizygotic pairs and 1.07 (0.66-1.73) in monozygotic pairs (Table 3).

The differences in ORs from the GEE model vs. conditional logistic model were statistically significant (OR 1.39, 95% CI 1.21-1.59, P<0.001) which suggesting that genetic

For peer review only - http://bmjopen.bmj.com/site/about/guidelines.xhtml

and early-life environment factors may play an important role in LBW-CMDs association.

(Insert Table 3 here)

Association between lifestyle-related factors and CMDs

In basic- and multi-adjusted GEE models, non-smoking, no/moderate alcohol drinking, active physical exercise, and non-overweight were individually related to a decreased risk of any CMD. When combining as a lifestyle index (unfavorable, intermediate and favorable), compared to an unfavorable lifestyle profile, an intermediate and a favorable lifestyle profile were significantly associated with a lower risk of any CMD, ORs (95% CIs) were 0.62 (0.55-0.69) and 0.40 (0.35-0.47), respectively. (Table 4).

(Insert Table 4 here)

Joint effect of LBW and healthy lifestyle factors on CMD risk

In joint effect analysis, the multi-adjusted ORs (95% CIs) of CMDs were 1.25 (0.96-1.62) for participants with LBW plus a favorable lifestyle profile, 1.94 (1.64-2.28) for those with LBW plus an intermediate lifestyle profile, and 3.47 (2.72-4.43) for those with LBW plus an unfavorable lifestyle profile (reference: those non-LBW plus a favorable lifestyle profile) (Figure 1 and Supplemental Table S3).

The additive interaction between the unfavorable lifestyle profile and LBW on CMDs was statistically significant (AP 0.199, 95% CI 0.016-0.381, P=0.03; S 1.506, 1.001-2.267, P<0.001), indicating that if people with LBW have a favorable or intermediate lifestyle, the risk of LBW on CMDs can be reduced by 20% (Supplemental Table S4).

(Insert Figure 1 here)

Supplementary analysis

The results were not much altered compared to those from initial analysis when we repeated following analyses by: 1) further performing stratified analysis by sex to address possible sex differences in the CMDs[34] (Supplemental Table S5), 2) additional adjustment for survival

For peer review only - http://bmjopen.bmj.com/site/about/guidelines.xhtml

status considering the association between LBW and mortality[35] (Supplemental Table S6), 3) excluding participants with CMDs before SALT recruitment (n=1748) (Supplemental Table S7), and 4) excluding data with missing values for covariates (n=1430) (Supplemental Table S8).

Discussion

In this large-scale, prospective, population-based nested case-control study of Swedish twins, we found that: 1) LBW was associated with an increased risk of CMDs including coronary heart disease, heart failure, ischemic stroke, and T2DM in adulthood, and the risk was became higher when multiple CMDs were co-occurring; 2) Genetic background and early life environmental factors appear to account for the LBW-CMDs association; and 3) A favorable lifestyle profile may modify the risk effect of LBW on CMDs.

In the past two decades, the relationship between birth weight and T2DM[11,12,36] has been well documented. However, the findings of the association between birth weight and coronary heart disease have been inconsistent. Three cohort studies have illustrated the relationship between LBW and the risk of coronary heart disease.[9,10,37] By contrast, Banci et al found higher birth weight was associated with a higher risk of coronary heart disease.[13] Another study showed there was no relationship between them.[14] In addition, evidence on the relationship between LBW and heart failure or ischemic stroke is sparse. To our knowledge, no studies have investigated the association of LBW with the risk of CMDs. In the present study, we found that LBW was associated with about 10-40% increased risk of coronary heart disease, heart failure, ischemic stroke (not hemorrhagic stroke), and T2DM. Further, we examined the relationship between birth weight and the risk of combined CMDs and found that the risk of any CMD related to LBW was almost 40% higher than those with non-LBW.

Potential contribution of genetic susceptibility and early-life environmental factors to the LBW-CMDs association is still unclear. Previous twin cohort studies showed that LBW was

For peer review only - http://bmjopen.bmj.com/site/about/guidelines.xhtml

associated with an increased risk of CVD or T2DM when twins were considered as independent individuals. This association only held in outcome-discordant dizygotic twins but not in monozygotic twin pairs, suggesting that genetic mechanisms played a role in this association.[12,28,38] In present study, we found that the LBW-CMDs association became non-significant in both dizygotic and monozygotic twin pairs by using co-twin matched analyses. These results illustrated that early-life environmental factors could also play an important role in the association between LBW and subsequent CMDs, in addition to genetic background.

Modifiable lifestyle factors (such as smoking, drinking, physical exercise and BMI) deserve to be studied in LBW-CMDs association. Thus far, only few studies focused on the joint effect of LBW with lifestyle factors on T2DM.[23,24,39] One of the studies included 149794 participants from three large prospective cohorts showed that LBW and unhealthy adulthood lifestyles encompassing smoking, non-moderate alcohol consumption, lower exercise intensity and BMI ≥25 were jointly related to an increased risk of T2DM.[24] Another cohort study indicated that the risk of LBW on diabetes could be eliminated in those with high physical activity level,[23] and individuals predisposed to T2DM due to LBW can be protected from glucose intolerance by regular exercise.[39] However, no study has illustrated the joint effect of LBW and healthy lifestyle on subsequent CMDs. In the present study, we found that people with LBW and an intermediate or a favorable lifestyle profile (including non-smoking, no/mild alcohol consumption, active physical exercise, and non-overweight) had a significantly lower risk of CMDs than those who had LBW and unfavorable lifestyle profile. To our knowledge, this is the first study to provide evidence that a healthy lifestyle might compensate for the risk effect of LBW on CMDs.

Several mechanisms may explain the relationship between LBW and the risk of CMDs. The "fetal origins hypothesis" has suggested that fetal malnutrition in middle to late gestation may generate a compensatory "survival" mechanism to redirect scant energy supplies from

For peer review only - http://bmjopen.bmj.com/site/about/guidelines.xhtml

muscle to vital tissues, causing permanent alterations in physiology, metabolism, and structure.[40,41] Additionally, some genes (such as insulin class I allele or variant of mitochondrial DNA) were found to lead to both birth weight loss and insulin resistance.[42,43] All of these alterations could result in an increased risk of CVD and T2DM in adulthood. Moreover, a haplotype of the glucocorticoid receptor gene may modify the association between size at birth and glucose tolerance, consequently T2MD occurrence.[44] However, maintaining a healthy lifestyle in adulthood may mitigate the risk of CMDs by improving insulin sensitivity and body composition, as well as controlling glycemic, blood pressure, and lipid profile.[45]

Strengths and Limitations

Notable strengths of our study involve the large nationwide population-based twin cohort, which provided an extraordinary opportunity to explore the association between LBW and the risk of CMDs in adulthood by controlling for some unmeasured confounders, such as genetic background and early-life environmental factors. Furthermore, this study on compensatory factors for the risk effect of LBW on CMDs is unique. Nevertheless, some limitations need to be pointed out. First, hypertension was defined only based on self-report from NPR, subjects with undiagnosed hypertension might have been misclassified as hypertension-free. Thus, CMDs only included heart disease, stroke, and T2DM in current study. Second, the data on birth weight were based on self-reports and non-differential misclassification among different birth weights groups could not be ruled out, possibly leading to an underestimation of the observed associations. Third, some prenatal factors (such as maternal smoking during pregnancy or premature birth) could not be controlled for, as information on these factors were not available. In addition, potential variations of lifestyle factors during follow-up could not be assessed. Finally, diet could be partially taken into account, as it is closely associated with other lifestyle factors such as smoking, alcohol consumption, physical exercise, and

For peer review only - http://bmjopen.bmj.com/site/about/guidelines.xhtml

BMI.[46] However, data on diet was not available.

Conclusion

This study provides evidence that LBW is associated with increased risk of CMDs including coronary heart disease, heart failure, ischemic stroke, and T2DM. The risk of CMDs related to LBW tends to increase with the number of co-occurring CMDs. Further, genetic and early-life environmental factors play an important role in the LBW-CMDs association. However, a favorable lifestyle involving non-smoking, no/mild alcohol consumption, active physical exercise, and BMI<25 may compensate the risk effect of LBW on CMDs. Our findings highlight the need for monitoring and controlling LBW for the prevention of CMDs, and the importance of maintaining a favorable lifestyle profile in people with LBW in adulthood to reduce risk of CMDs.

For peer review only

For peer review only - http://bmjopen.bmj.com/site/about/guidelines.xhtml

Acknowledgements

We would like to thank the Swedish Twin Registry for access to data and are grateful to all the twins who took part in the study, as well as the members of the survey teams. We are grateful to Prof. Nancy L Pedersen for her great contribution to the design of the twin data collection. The Swedish Twin Registry is managed by Karolinska Institutet and receives funding through the Swedish Research Council under the grant No. 2017-00641.

Contributors

WX conceptualized and designed the study. XL conducted the literature search, analyzed the data, and wrote the first draft. XL, RY, HX, RS, XQ, and WX contributed to the discussion and interpretation of the results. WX and XQ were involved in study supervision. All authors contributed to critical revision of the manuscript for important intellectual content and gave their final approval of the version to be published. WX obtained funding for the study. XL and WX had full access to all the data in the study and take responsibility for the integrity of the data and the accuracy of the data analysis.

Funding

This work was supported by grants from the Swedish Research Council (No. 2017-00981), the National Natural Science Foundation of China (No. 81771519), the Konung Gustaf V:s och Drottning Victorias Frimurare Foundation (No. 2016-2020), Demensfonden, Strokefonden, Cornells Stiftelse and Alzheimerfonden (2018-2019). This project is part of CoSTREAM (www.costream.eu) and received funding from the European Union's Horizon 2020 research and innovation program under grant agreement No 667375. The funders had no role in the design and conduct of the study; collection, management, analysis, and interpretation of the data; preparation, review, or approval of the manuscript; and decision to submit the manuscript for publication.

Competing interests

For peer review only - http://bmjopen.bmj.com/site/about/guidelines.xhtml

There are no competing interests for any author.

Patient consent for publication

Not applicable.

Ethics approval

The approval from the Ethics Committee of the Karolinska Institutet was obtained (Dnr: 97: 051)

Data availability statement

Data are available upon reasonable request.

For peer review only - http://bmjopen.bmj.com/site/about/guidelines.xhtml

Reference

1. Kivimäki M, Kuosma E, Ferrie JE*, et al.* Overweight, obesity, and risk of cardiometabolic multimorbidity: pooled analysis of individual-level data for 120813 adults from 16 cohort studies from the USA and Europe. *The Lancet Public Health.* 2017;2:e277-e285.
2. Benjamin EJ, Muntner P, Alonso A*, et al.* Heart Disease and Stroke Statistics-2019 Update: A Report From the American Heart Association. *Circulation.* 2019;139:e56-e528.
3. Disease GBD, Injury I, Prevalence C. Global, regional, and national incidence, prevalence, and years lived with disability for 328 diseases and injuries for 195 countries, 1990-2016: a systematic analysis for the Global Burden of Disease Study 2016. *Lancet.* 2017;390:1211-1259.
4. WHO. global report diabetes. *http://wwwwhoint/diabetes/global-report/en/.*
5. Wang Z, Marseglia A, Shang Y*, et al.* Leisure activity and social integration mitigate the risk of dementia related to cardiometabolic diseases: A population-based longitudinal study. *Alzheimers Dement.* 2020;16:316-325.
6. Keenan T, Zhao W, Rasheed A*, et al.* Causal Assessment of Serum Urate Levels in Cardiometabolic Diseases Through a Mendelian Randomization Study. *J Am Coll Cardiol.* 2016;67:407-416.
7. Smith CJ, Ryckman KK, Barnabei VM*, et al.* The impact of birth weight on cardiovascular disease risk in the Women's Health Initiative. *Nutr Metab Cardiovasc Dis.* 2016;26:239-245.
8. Lawani SO, Demerath EW, Lopez FL*, et al.* Birth weight and the risk of atrial fibrillation in whites and African Americans: the Atherosclerosis Risk In Communities (ARIC) study. *BMC Cardiovasc Disord.* 2014;14:69.

For peer review only

For peer review only - http://bmjopen.bmj.com/site/about/guidelines.xhtml

9. Rich-Edwards JW, Kleinman K, Michels KB, *et al.* Longitudinal study of birth weight and adult body mass index in predicting risk of coronary heart disease and stroke in women. *Bmj.* 2005;330:1115.

10. Lawlor DA, Ronalds G, Clark H, *et al.* Birth weight is inversely associated with incident coronary heart disease and stroke among individuals born in the 1950s: findings from the Aberdeen Children of the 1950s prospective cohort study. *Circulation.* 2005;112:1414-1418.

11. Whincup PH, Kaye SJ, Owen CG, *et al.* Birth Weight and Risk of Type 2 Diabetes. *Jama.* 2008;300:2886.

12. Johansson S, Iliadou A, Bergvall N, *et al.* The association between low birth weight and type 2 diabetes: contribution of genetic factors. *Epidemiology.* 2008;19:659-665.

13. Banci M, Saccucci P, Dofcaci A, *et al.* Birth weight and coronary artery disease. The effect of gender and diabetes. *Int J Biol Sci.* 2009;5:244-248.

14. Morley R, McCalman J, Carlin JB. Birthweight and coronary heart disease in a cohort born 1857-1900 in Melbourne, Australia. *International journal of epidemiology.* 2006;35:880-885.

15. Marenberg ME, Risch N, Berkman LF, *et al.* Genetic susceptibility to death from coronary heart disease in a study of twins. *N Engl J Med.* 1994;330:1041-1046.

16. Almgren P, Lehtovirta M, Isomaa B, *et al.* Heritability and familiality of type 2 diabetes and related quantitative traits in the Botnia Study. *Diabetologia.* 2011;54:2811-2819.

17. Bak S, Gaist D, Sindrup SH, *et al.* Genetic liability in stroke: a long-term follow-up study of Danish twins. *Stroke.* 2002;33:769-774.

18. Lunde A, Melve KK, Gjessing HK, *et al.* Genetic and environmental influences on birth weight, birth length, head circumference, and gestational age by use of

For peer review only - http://bmjopen.bmj.com/site/about/guidelines.xhtml

population-based parent-offspring data. *Am J Epidemiol.* 2007;165:734-741.

19. Xu W, Qiu C, Gatz M*, et al.* Mid- and late-life diabetes in relation to the risk of dementia: a population-based twin study. *Diabetes.* 2009;58:71-77.

20. Yang R, Pedersen NL, Bao C*, et al.* Type 2 diabetes in midlife and risk of cerebrovascular disease in late life: a prospective nested case-control study in a nationwide Swedish twin cohort. *Diabetologia.* 2019;62:1403-1411.

21. Lv J, Yu C, Guo Y*, et al.* Adherence to Healthy Lifestyle and Cardiovascular Diseases in the Chinese Population. *J Am Coll Cardiol.* 2017;69:1116-1125.

22. Lv J, Yu C, Guo Y*, et al.* Adherence to a healthy lifestyle and the risk of type 2 diabetes in Chinese adults. *Int J Epidemiol.* 2017;46:1410-1420.

23. Hu CY, Mu YM, Wan Q*, et al.* Association between birth weight and diabetes: Role of body mass index and lifestyle in later life. *Journal of Diabetes.* 2020;12:10-20.

24. Li Y, Ley SH, Tobias DK*, et al.* Birth weight and later life adherence to unhealthy lifestyles in predicting type 2 diabetes: prospective cohort study. *BMJ.* 2015;351:h3672.

25. Lichtenstein P, De Faire U, Floderus B*, et al.* The Swedish Twin Registry: a unique resource for clinical, epidemiological and genetic studies. *J Intern Med.* 2002;252:184-205.

26. Patientregistret - Socialstyrelsen n.d. https://www.socialstyrelsen.se/statistik-och-data/register/alla-register/patientregistret/ Accessed Octorber 20, 2020.

27. Xiao X, Zhang ZX, Cohen HJ*, et al.* Evidence of a Relationship Between Infant Birth Weight and Later Diabetes and Impaired Glucose Regulation in a Chinese Population. *Diabetes Care.* 2007;31:483-487.

28. Hubinette A, Cnattingius S, Johansson ALV*, et al.* Birth weight and risk of angina pectoris: Analysis in Swedish twins. *European Journal of Epidemiology.*

For peer review only - http://bmjopen.bmj.com/site/about/guidelines.xhtml

2002;18:539-544.

29. Carlsson S, Ahlbom A, Lichtenstein P, *et al.* Shared genetic influence of BMI, physical activity and type 2 diabetes: a twin study. *Diabetologia.* 2013;56:1031-1035.

30. Nyberg ST, Singh-Manoux A, Pentti J, *et al.* Association of Healthy Lifestyle With Years Lived Without Major Chronic Diseases. *Jama Internal Medicine.* 2020;180:760-768.

31. Kato K, Sullivan PF, Evengard B, *et al.* Premorbid predictors of chronic fatigue. *Archives of General Psychiatry.* 2006;63:1267-1272.

32. Xu WL, Atti AR, Gatz M, *et al.* Midlife overweight and obesity increase late-life dementia risk: a population-based twin study. *Neurology.* 2011;76:1568-1574.

33. Bao C, Yang R, Pedersen NL, *et al.* Overweight in midlife and risk of cancer in late life: A nationwide Swedish twin study. *Int J Cancer.* 2019;144:2128-2134.

34. Kuznetsova T. Sex Differences in Epidemiology of Cardiac and Vascular Disease. *Adv Exp Med Biol.* 2018;1065:61-70.

35. Hoy WE, Nicol JL. The Barker hypothesis confirmed: association of low birth weight with all-cause natural deaths in young adult life in a remote Australian Aboriginal community. *J Dev Orig Health Dis.* 2019;10:55-62.

36. Kaijser M, Bonamy AK, Akre O, *et al.* Perinatal risk factors for diabetes in later life. *Diabetes.* 2009;58:523-526.

37. Andersen LG, Angquist L, Eriksson JG, *et al.* Birth weight, childhood body mass index and risk of coronary heart disease in adults: combined historical cohort studies. *PLoS One.* 2010;5:e14126.

38. Oberg S, Cnattingius S, Sandin S, *et al.* Birth weight predicts risk of cardiovascular disease within dizygotic but not monozygotic twin pairs: a large population-based co-twin-control study. *Circulation.* 2011;123:2792-2798.

For peer review only - http://bmjopen.bmj.com/site/about/guidelines.xhtml

39. Eriksson JG, Yliharsila H, Forsen T, *et al.* Exercise protects against glucose intolerance in individuals with a small body size at birth. *Prev Med.* 2004;39:164-167.

40. Barker DJ. Fetal origins of coronary heart disease. *BMJ.* 1995;311:171-174.

41. Poulsen P, Vaag A. Glucose and insulin metabolism in twins: influence of zygosity and birth weight. *Twin Res.* 2001;4:350-355.

42. Dunger DB, Ong KK, Huxtable SJ, *et al.* Association of the INS VNTR with size at birth. ALSPAC Study Team. Avon Longitudinal Study of Pregnancy and Childhood. *Nat Genet.* 1998;19:98-100.

43. Casteels K, Ong K, Phillips D, *et al.* Mitochondrial 16189 variant, thinness at birth, and type-2 diabetes. *Lancet.* 1999;353:1499-1500.

44. Rautanen A, Eriksson JG, Kere J, *et al.* Associations of body size at birth with late-life cortisol concentrations and glucose tolerance are modified by haplotypes of the glucocorticoid receptor gene. *J Clin Endocrinol Metab.* 2006;91:4544-4551.

45. Marwick TH, Hordern MD, Miller T, *et al.* Exercise training for type 2 diabetes mellitus: impact on cardiovascular risk: a scientific statement from the American Heart Association. *Circulation.* 2009;119:3244-3262.

46. Fransen HP, Boer JMA, Beulens JWJ, *et al.* Associations between lifestyle factors and an unhealthy diet. *Eur J Public Health.* 2017;27:274-278.

For peer review only

For peer review only - http://bmjopen.bmj.com/site/about/guidelines.xhtml

Table 1. Characteristics of the study population (n=19779) by birth weight

Characteristics	<2.0 kg n = 3998	2.0-3.0 kg n = 11510	>3.0 kg n = 4271	*P*-value
Age (years), mean (SD)	57.37 (9.6)	55.07 (8.8)	54.70 (8.9)	<0.001
Male sex, n (%)	1307 (32.7)	3504 (30.4)	2042 (47.8)	<0.001
Education, n (%)				
<8 years	1251 (31.3)	2850 (24.8)	1009 (23.6)	<0.001
≥8 years	2747 (68.7)	8660 (75.2)	3262 (76.4)	
Marital status, n (%)				
Married/cohabited	2911 (72.8)	8749 (76.0)	3298 (77.2)	<0.001
Single	1087 (27.2)	2761 (24.0)	973 (22.8)	
Zygosity, n (%)				
Monozygosity	1027 (25.7)	2647 (23.0)	685 (16.0)	<0.001
Dizygosity	2384 (59.6)	7436 (64.6)	3021 (70.7)	
Undetermined	587 (14.7)	1427 (12.4)	565 (13.2)	
BMI, mean (SD)	25.02 (3.8)	24.67 (3.5)	25.13 (3.5)	<0.001
BMI, n (%)				
<18.5 (Underweight)	71 (1.8)	167 (1.4)	46 (1.1)	<0.001
18.5-24.9 (Normal weight)	2108 (52.7)	6600 (57.3)	2218 (52.0)	
25.0-29.9 (Overweight)	1439 (36.0)	3874 (33.7)	1623 (38.0)	
≥30 (Obese)	380 (9.5)	869 (7.6)	384 (9.0)	
Smoking status, n (%)				
Never smoked	2049 (51.2)	5825 (50.6)	1932 (45.2)	<0.001
Former/current smoker	1949 (48.8)	5685 (49.4)	2339 (54.8)	
Alcohol consumption, n (%)				
No/mild drinking	3735 (93.4)	10746 (93.4)	3884 (90.9)	<0.001
Heavy drinking	263 (6.6)	764 (6.6)	387 (9.1)	
Active physical exercise, n (%)				
No	2092 (52.3)	5736(49.8)	2101 (49.2)	0.008
Yes	1905 (48.2)	5774 (50.2)	2170 (50.8)	
Hypertension, n (%)	1299 (33.5)	2954 (25.7)	1023 (24.0)	<0.001

Data were presented as means ± standard deviations or number (%).

Abbreviations: BMI, body mass index; SD, standard deviation.

For peer review only

For peer review only - http://bmjopen.bmj.com/site/about/guidelines.xhtml

Table 2. Odds ratios (ORs) and 95% confidence intervals (CIs) of birth weight in relation to different subtypes of heart diseases, stroke, and diabetes in adulthood: results from Generalized Estimating Equation

Single/combined CMDs	No. of Cases	OR (95% CI) *	OR (95% CI) †
Subtypes of Heart disease			
CHD			
<2.0	622	1.33 (1.19-1.49)	1.27 (1.14-1.43)
2.0-3.0	1166	Reference	Reference
>3.0	497	1.07 (0.95-1.20)	1.08 (0.95-1.22)
HF			
<2.0	214	1.36 (1.13-1.63)	1.27 (1.05-1.53)
2.0-3.0	356	Reference	Reference
>3.0	143	1.13 (0.93-1.39)	1.12 (0.91-1.38)
Subtypes of Stroke			
IS			
<2.0	432	1.20 (1.06-1.36)	1.14 (1.01-1.30)
2.0-3.0	874	Reference	Reference
>3.0	352	1.10 (0.96-1.26)	1.12 (0.98-1.29)
HS			
<2.0	74	1.14 (0.86-1.50)	1.09 (0.82-1.44)
2.0-3.0	162	Reference	Reference
>3.0	59	0.97 (0.72-1.32)	0.99 (0.73-1.34)
T2DM			
<2.0	668	1.45 (1.30-1.61)	1.39 (1.24-1.55)
2.0-3.0	1219	Reference	Reference
>3.0	424	0.88 (0.78-0.99)	0.82 (0.72-0.93)
Any CMDs (CHD, HF, IS, T2DM)			
<2.0	1423	1.44 (1.32-1.57)	1.39 (1.27-1.52)
2.0-3.0	2797	Reference	Reference
>3.0	1115	1.06 (0.97-1.16)	1.05 (0.96-1.16)

Abbreviations: CHD, coronary heart disease; CMDs, cardiometabolic diseases; HF, heart failure; HS, hemorrhagic stroke; IS, Ischemic stroke; T2DM, type 2 diabetes mellitus.

* Adjusted for age, sex, and education.

† Adjusted for age, sex, education, body mass index, smoking, alcohol consumption, marital status, physical exercise, and hypertension.

For peer review only - http://bmjopen.bmj.com/site/about/guidelines.xhtml

Table 3. Odds ratios (ORs) and 95% confidence intervals (CIs) for the association between LBW and adult CMDs in co-twin control analysis using CMDs discordant twin pairs: results from conditional logistic regression

Co-twin control	Co-twin with CMDs					
	**All zygosity twins** * **(n=1293 pairs)**		**Dizygotic only (n=845 pairs)**		**Monozygotic only (n=290 pairs)**	
	Non-LBW	LBW	Non-LBW	LBW	Non-LBW	LBW
Non-LBW	804	177	549	106	162	46
LBW	153	159	90	100	45	37
Basic-adjusted OR (95% CI) †	1.20 (0.96-1.49)		1.25 (0.94-1.67)		1.03 (0.68-1.56)	
Multi-adjusted OR (95% CI) ‡	1.21 (0.94-1.56)		1.34 (0.96-1.89)		1.07 (0.66-1.73)	

Abbreviations: CMDs, cardiometabolic diseases; LBW, low birth weight.

* Contain 158 pairs of undetermined zygosity twins

† Adjusted for sex and education.

‡ Adjusted for sex, education, body mass index, smoking, alcohol consumption, marital status, physical exercise, and hypertension.

For peer review only

For peer review only - http://bmjopen.bmj.com/site/about/guidelines.xhtml

Table 4. Odds ratios (ORs) and 95% confidence intervals (CIs) of smoking, alcohol consumption, physical exercise, and body mass index (BMI) related to cardiometabolic diseases from Generalized Estimating Equation models

Lifestyle factors	No. of Cases *	OR (95% CI) †	OR (95% CI) ‡
**Smoking**			
Yes	1886	Reference	Reference
No	1751	0.81 (0.74-0.87)	0.80 (0.74-0.88)
**Alcohol consumption**			
Heavy drinking	312	Reference	Reference
No/mild drinking	3325	0.72 (0.62-0.83)	0.83 (0.71-0.97)
**Active physical exercise**			
No	1977	Reference	Reference
Yes	1660	0.74 (0.69-0.80)	0.85 (0.78-0.92)
**BMI**			
≥25 (Overweight)	2109	Reference	Reference
<25 (Non-overweight)	1528	0.50 (0.46-0.54)	0.59 (0.54-0.64)
**Lifestyle index (scored 0-4)**			
Unfavorable (0-1)	816	Reference	Reference
Intermediate (2-3)	2405	0.57 (0.51-0.63)	0.62 (0.55-0.69)
Favorable (4)	416	0.34 (0.30-0.40)	0.40 (0.35-0.47)
P for trend		<0.001	<0.001

* 1748 cases before Screening Across the Lifespan Twin study survey were exclude.

† Adjusted for age, sex, and education.

‡ Adjusted for age, sex, education, marital status, hypertension, and birth weight, as well as body mass index, smoking, alcohol consumption, and active physical exercise, if applicable.

For peer review only

For peer review only - http://bmjopen.bmj.com/site/about/guidelines.xhtml

Figure 1. Joint effect of low birth weight (LBW) and lifestyle (smoking status, alcohol consumption, active physical exercise, and body mass index) on cardiometabolic diseases (CMDs).

Multi-adjusted odds ratios (95% confidence interval) of CMDs in relation to joint exposure of LBW and lifestyle from Generalized Estimating Equation models (adjusted for age, sex, education, marital status, and hypertension).

[*] *P*-value<0.001 refers to the difference in the risk of CMDs between participants with LBW who have a favorable lifestyle vs. those with LBW who have an unfavorable lifestyle.

For peer review only

For peer review only - http://bmjopen.bmj.com/site/about/guidelines.xhtml

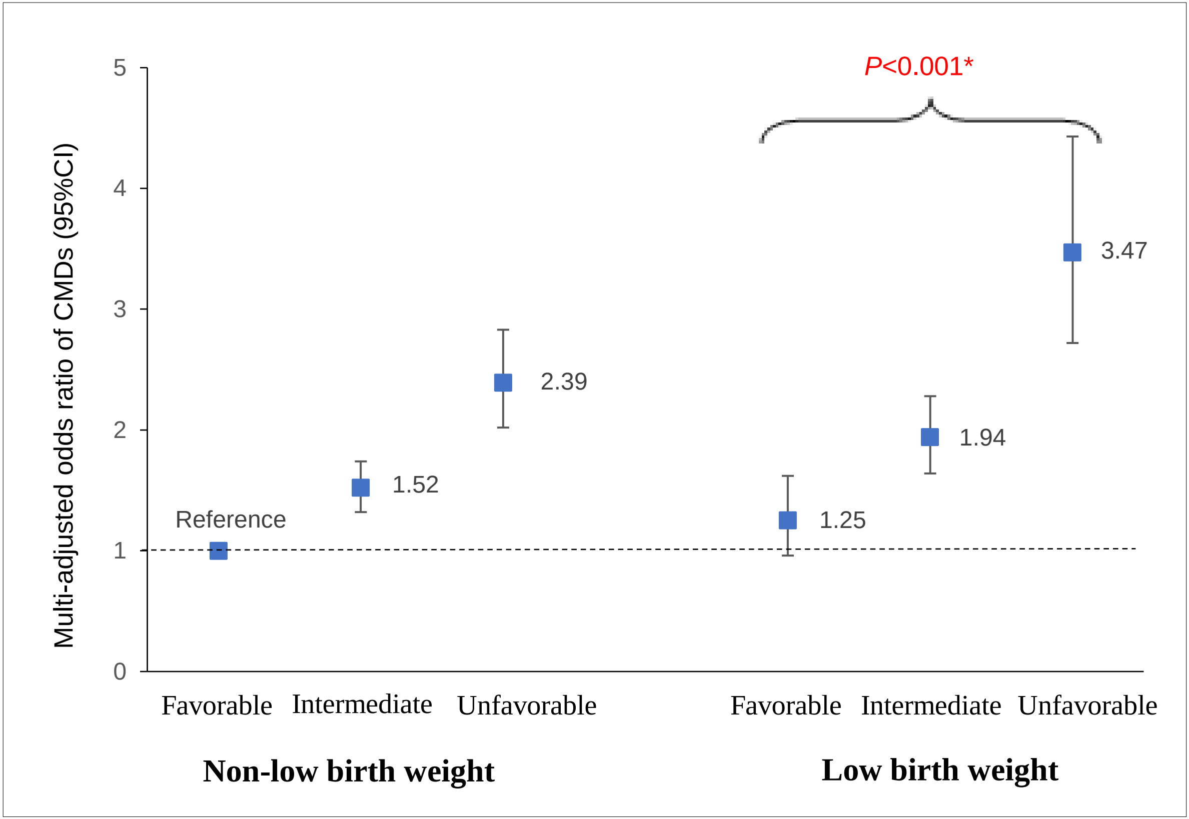

P<0.001*
Multi-adjusted odds ratio of CMDs (95%CI)
5
4
3
2
1
0
Reference
1.52
2.39
1.25
1.94
3.47
Favorable
Intermediate
Unfavorable
Favorable
Intermediate
Unfavorable
Non-low birth weight
Low birth weight

Supplemental Materials

Including: Tables-8; Figure-1

Table S1. The relationship between low birth weight and numbers of cardiometabolic diseases (CMDs): results from Generalized Estimating Equation

Table S2. The dose-dependent relationship between low birth weight and cardiometabolic disease: results from Generalized Estimating Equation

Table S3. Odds ratios (ORs) and 95% confidence intervals (CIs) of cardiometabolic diseases in relation to the joint exposure of lifestyle (smoking status, alcohol consumption, active physical exercise, and body mass index) and low birth weight (LBW) from Generalized Estimation Equation models

Table S4. Additive interaction between lifestyle (smoking status, alcohol consumption, active physical exercise, and body mass index) and low birth weight (LBW) for the risk of cardiometabolic diseases

Table S5 Odds ratios (ORs) and 95% confidence intervals (CIs) of birth weight in relation to CMDs by sex: results from Generalized Estimating Equation

Table S6. Odds ratios (ORs) and 95% confidence intervals (CIs) of birth weight in relation to CMDs in adulthood further adjusted for survival status: results from Generalized Estimating Equation models

Table S7. Odds ratios (ORs) and 95% confidence intervals (CIs) of birth weight in relation to cardiometabolic diseases in adulthood by excluding cardiometabolic diseases onset before screening: results from Generalized Estimating Equation (n=18301)

Table S8. Odds ratios (ORs) and 95% confidence intervals (CIs) of birth weight in relation to cardiometabolic diseases in adulthood by excluding data with missing values for covariate: results from Generalized Estimating Equation (n=18349)

Figure S1. Flow chart of the study population

For peer review only - http://bmjopen.bmj.com/site/about/guidelines.xhtml

Table S1. The relationship between low birth weight and numbers of cardiometabolic diseases (CMDs): results from Generalized Estimating Equation

CMDs status	No. of participants	Low birth weight		
		No. of cases	Basic-adjusted OR (95% CI) *	Multi-adjusted OR (95% CI) †
No	14444	2575	Reference	Reference
Any one	5335	1423	1.43 (1.31-1.55)	1.37 (1.25-1.50)
Only one	3932	989	1.32 (1.21-1.45)	1.28 (1.17-1.41)
Any two	1174	355	1.56 (1.36-1.80)	1.48 (1.28-1.72)
Any three or more	229	79	1.94 (1.47-2.56)	1.82 (1.37-2.42)
P for trend			<0.001	<0.001

* Adjusted for age, sex, and education.

† Adjusted for age, sex, education, body mass index, smoking, alcohol consumption, marital status, physical exercise, and hypertension.

For peer review only

For peer review only - http://bmjopen.bmj.com/site/about/guidelines.xhtml

Table S2. The dose-dependent relationship between low birth weight and cardiometabolic disease: results from Generalized Estimating Equation

Birth weight	No. of Case	Basic-adjusted OR (95% CI) *	Multi-adjusted OR (95% CI) †
Continuous		0.83 (0.79-0.88)	0.84 (0.80-0.89)
Categorical			
<1.7	622	1.54 (1.36-1.74)	1.45 (1.28-1.66)
1.7-2.0kg	801	1.35 (1.22-1.49)	1.32 (1.18-1.47)
≥2.0kg	3912	Reference	Reference
P for trend		<0.001	<0.001

* Adjusted for age, sex, and education.

† Adjusted for age, sex, education, body mass index, smoking, alcohol consumption, marital status, physical exercise, and hypertension.

For peer review only

For peer review only - http://bmjopen.bmj.com/site/about/guidelines.xhtml

Table S3. Odds ratios (ORs) and 95% confidence intervals (CIs) of cardiometabolic diseases in relation to the joint exposure of lifestyle (smoking status, alcohol consumption, active physical exercise, and body mass index) and low birth weight (LBW) from Generalized Estimation Equation models

Joint exposure		No. of subjects *	Cardiometabolic diseases		
Lifestyle index	LBW		Cases	Basic-adjusted OR (95% CI) †	Multi-adjusted OR (95% CI) ‡
Favorable	No	2533	314	Reference	Reference
Intermediate	No	9751	1795	1.65 (1.44-1.87)	1.52 (1.32-1.74)
Unfavorable	No	2274	620	2.90 (2.47-3.40)	2.39 (2.02-2.83)
Favorable	Yes	570	102	1.32 (1.03-1.70)	1.25 (0.96-1.62)
Intermediate	Yes	2362	610	2.18 (1.86-2.54)	1.94 (1.64-2.28)
Unfavorable	Yes	541	196	3.89 (3.08-4.90)	3.47 (2.72-4.43)

* 1748 cases before Screening Across the Lifespan Twin study survey were exclude.

† Adjusted for age, sex, education.

‡ Adjusted for age, sex, education, marital status, and hypertension.

For peer review only - http://bmjopen.bmj.com/site/about/guidelines.xhtml

Table S4. Additive interaction between lifestyle (smoking status, alcohol consumption, active physical exercise, and body mass index) and low birth weight (LBW) for the risk of cardiometabolic diseases

Joint exposure		No. of subjects *	Cardiometabolic diseases		
Lifestyle index	LBW		Cases	Basic-adjusted OR (95% CI) †	Multi-adjusted OR (95% CI) ‡
Favorable/Intermediate	No	12284	2109	Reference	Reference
Unfavorable	No	2274	620	1.91 (1.70-2.14)	1.68 (1.49-1.90)
Favorable/Intermediate	Yes	2932	712	1.33 (1.20-1.47)	1.28 (1.14-1.42)
Unfavorable	Yes	541	196	2.56 (2.09-3.15)	2.44 (1.97-3.03)

* 1748 cases before Screening Across the Lifespan Twin study survey were exclude.

† Adjusted for age, sex, education.

‡ Adjusted for age, sex, education, marital status, and hypertension.

Measures of additive interaction for cardiometabolic diseases:

Relative excess risk due to interaction: 0.485, 95%CI: -0.044–1.014, P=0.07;

Attributable proportion due to interaction: 0.199, 95%CI: 0.016–0.381, P=0.03;

Synergy index: 1.506, 95% CI: 1.001–2.267, P<0.001.

For peer review only

Table S5. Odds ratios (ORs) and 95% confidence intervals (CIs) of birth weight in relation to CMDs by sex: results from Generalized Estimating Equation

Birth weight (kg)	No. of Cases	OR (95% CI) *	OR (95% CI) †
Male			
<2.0	564	1.39 (1.20-1.61)	1.44 (1.23-1.69)
2.0-3.0	1050	Reference	Reference
>3.0	642	1.06 (0.93-1.21)	1.07 (0.93-1.23)
Female			
<2.0	859	1.47 (1.32-1.63)	1.36 (1.21-1.52)
2.0-3.0	1747	Reference	Reference
>3.0	473	1.06 (0.94-1.19)	1.04 (0.91-1.19)

* Adjusted for age, sex, and education.

† Adjusted for age, sex, education, body mass index, smoking, alcohol consumption, marital status, physical exercise, and hypertension.

For peer review only

For peer review only - http://bmjopen.bmj.com/site/about/guidelines.xhtml

Table S6. Odds ratios (ORs) and 95% confidence intervals (CIs) of birth weight in relation to CMDs in adulthood further adjusted for survival status: results from Generalized Estimating Equation models

Birth weight (kg)	No. of Cases	OR (95% CI) *
<2.0	1423	1.38 (1.26-1.52)
2.0-3.0	2797	Reference
>3.0	1115	1.05 (0.95-1.16)

* Adjusted for age, sex, education, body mass index, smoking, alcohol consumption, marital status, physical exercise, hypertension, and death.

For peer review only

Table S7. Odds ratios (ORs) and 95% confidence intervals (CIs) of birth weight in relation to cardiometabolic diseases in adulthood by excluding cardiometabolic diseases onset before screening: results from Generalized Estimating Equation (n=18301)

Birth weight (kg)	No. of Cases	OR (95% CI) *	OR (95% CI) †
<2.0	908	1.34 (1.22-1.48)	1.30 (1.17-1.45)
2.0-3.0	1969	Reference	Reference
>3.0	760	1.03 (0.93-1.13)	1.02 (0.92-1.14)

* Adjusted for age, sex, and education.

† Adjusted for age, sex, education, body mass index, smoking, alcohol consumption, marital status, physical exercise, and hypertension.

For peer review only

For peer review only - http://bmjopen.bmj.com/site/about/guidelines.xhtml

Table S8. Odds ratios (ORs) and 95% confidence intervals (CIs) of birth weight in relation to cardiometabolic diseases in adulthood by excluding data with missing values for covariate: results from Generalized Estimating Equation (n=18349)

Birth weight (kg)	No. of Cases	OR (95% CI) *	OR (95% CI) †
<2.0	1184	1.49 (1.36-1.63)	1.43 (1.30-1.58)
2.0-3.0	2359	Reference	Reference
>3.0	937	1.05 (0.96-1.15)	1.03 (0.93-1.14)

* Adjusted for age, sex, and education.

† Adjusted for age, sex, education, body mass index, smoking, alcohol consumption, marital status, physical exercise, and hypertension.

For peer review only

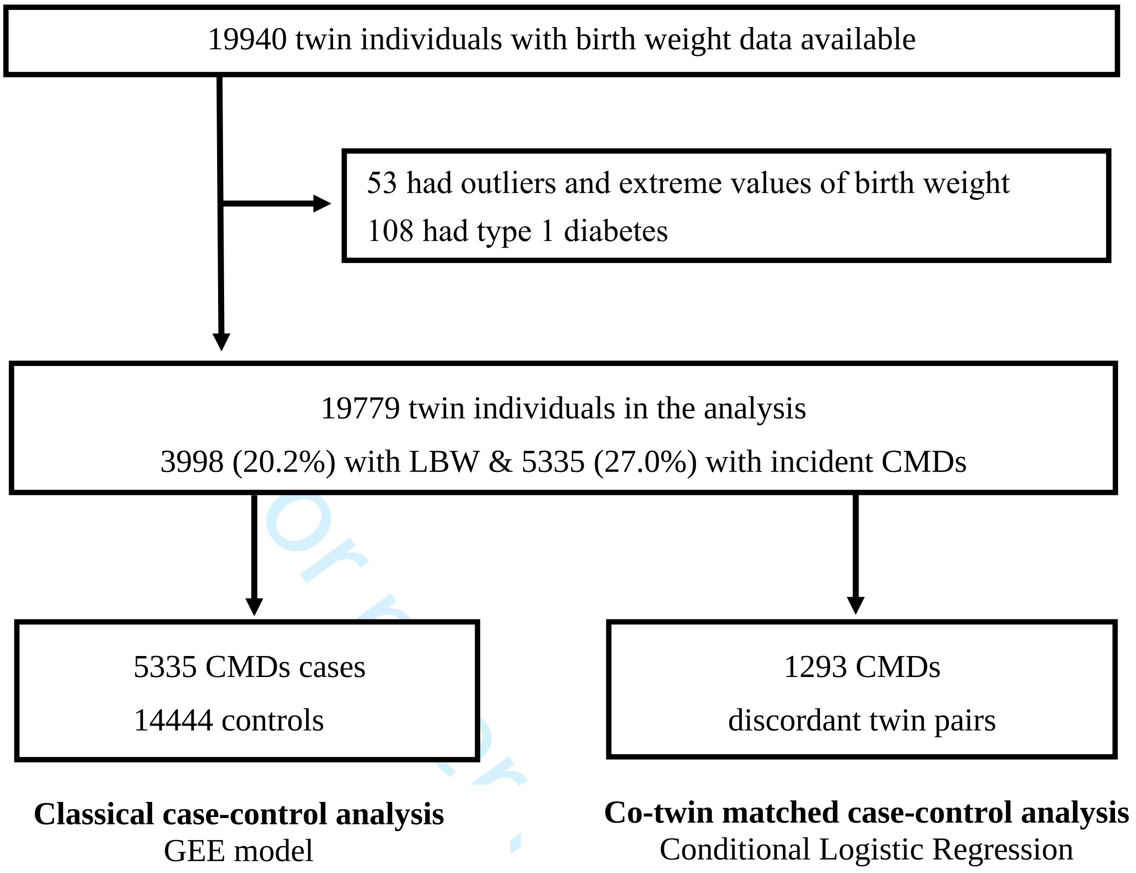

Figure S1. Flow chart of the study population

Abbreviations: LBW, low birth weight; CMDs, cardiometabolic diseases; GEE, generalized estimating equation.

For peer review only - http://bmjopen.bmj.com/site/about/guidelines.xhtml

STROBE Statement—Checklist of items that should be included in reports of ***case-control studies***

	Item No	Recommendation	Page No
**Title and abstract**	1	(*a*) Indicate the study's design with a commonly used term in the title or the abstract	2
		(*b*) Provide in the abstract an informative and balanced summary of what was done and what was found	2-3
**Introduction**			
Background/rationale	2	Explain the scientific background and rationale for the investigation being reported	4-5
Objectives	3	State specific objectives, including any prespecified hypotheses	5
**Methods**			
Study design	4	Present key elements of study design early in the paper	5
Setting	5	Describe the setting, locations, and relevant dates, including periods of recruitment, exposure, follow-up, and data collection	5-8
Participants	6	(*a*) Give the eligibility criteria, and the sources and methods of case ascertainment and control selection. Give the rationale for the choice of cases and controls	6-7
		(*b*) For matched studies, give matching criteria and the number of controls per case	8-9
Variables	7	Clearly define all outcomes, exposures, predictors, potential confounders, and effect modifiers. Give diagnostic criteria, if applicable	6-9
Data sources/ measurement	8*	For each variable of interest, give sources of data and details of methods of assessment (measurement). Describe comparability of assessment methods if there is more than one group	6-8
Bias	9	Describe any efforts to address potential sources of bias	8-9
Study size	10	Explain how the study size was arrived at	5
Quantitative variables	11	Explain how quantitative variables were handled in the analyses. If applicable, describe which groupings were chosen and why	6
Statistical methods	12	(*a*) Describe all statistical methods, including those used to control for confounding	8-9
		(*b*) Describe any methods used to examine subgroups and interactions	8-9
		(*c*) Explain how missing data were addressed	8
		(*d*) If applicable, explain how matching of cases and controls was addressed	8
		(*e*) Describe any sensitivity analyses	11-12
**Results**			
Participants	13*	(a) Report numbers of individuals at each stage of study—eg numbers potentially eligible, examined for eligibility, confirmed eligible, included in the study, completing follow-up, and analysed	9-10
		(b) Give reasons for non-participation at each stage	-
		(c) Consider use of a flow diagram	Supplemental Figure S1
Descriptive data	14*	(a) Give characteristics of study participants (eg demographic, clinical, social) and information on exposures and potential confounders	9-10
		(b) Indicate number of participants with missing data for each variable	-

For peer review only - http://bmjopen.bmj.com/site/about/guidelines.xhtml

		of interest	
Outcome data	15*	Report numbers in each exposure category, or summary measures of exposure	9
Main results	16	(*a*) Give unadjusted estimates and, if applicable, confounder-adjusted estimates and their precision (eg, 95% confidence interval). Make clear which confounders were adjusted for and why they were included	10-11
		(*b*) Report category boundaries when continuous variables were categorized	10
		(*c*) If relevant, consider translating estimates of relative risk into absolute risk for a meaningful time period	-
Other analyses	17	Report other analyses done—eg analyses of subgroups and interactions, and sensitivity analyses	11-12
**Discussion**			
Key results	18	Summarise key results with reference to study objectives	12
Limitations	19	Discuss limitations of the study, taking into account sources of potential bias or imprecision. Discuss both direction and magnitude of any potential bias	14-15
Interpretation	20	Give a cautious overall interpretation of results considering objectives, limitations, multiplicity of analyses, results from similar studies, and other relevant evidence	12-15
Generalisability	21	Discuss the generalisability (external validity) of the study results	14
**Other information**			
Funding	22	Give the source of funding and the role of the funders for the present study and, if applicable, for the original study on which the present article is based	16

*Give information separately for cases and controls.

Note: An Explanation and Elaboration article discusses each checklist item and gives methodological background and published examples of transparent reporting. The STROBE checklist is best used in conjunction with this article (freely available on the Web sites of PLoS Medicine at http://www.plosmedicine.org/, Annals of Internal Medicine at http://www.annals.org/, and Epidemiology at http://www.epidem.com/). Information on the STROBE Initiative is available at http://www.strobe-statement.org.

For peer review only

For peer review only - http://bmjopen.bmj.com/site/about/guidelines.xhtml

BMJ Open

Association of low birth weight with cardiometabolic diseases in the Swedish twins: A population-based cohort study

Journal:	*BMJ Open*
Manuscript ID	bmjopen-2020-048030.R1
Article Type:	Original research
Date Submitted by the Author:	22-Apr-2021
Complete List of Authors:	Li, Xuerui; Tianjin Medical University, Department of Epidemiology and Biostatistics, School of Public Health<br>Yang, Rongrong; Tianjin University of Traditional Chinese Medicine, Public Health Science and Engineering College<br>Yang, Wenzhe; Tianjin Medical University, Department of Epidemiology and Biostatistics, School of Public Health<br>Xu, Hui; Beijing Children's Hospital Capital Medical University, Big Data and Engineering Research Center<br>Song, Ruixue; Tianjin Medical University, Department of Epidemiology and Biostatistics, School of Public Health<br>Qi, Xiuying; Tianjin Medical University, Department of Epidemiology and Biostatistics, School of Public Health<br>Xu, Weili; Karolinska Institutet, Aging Research Center, Department of Neurobiology, Health Care Sciences and Society; Tianjin Medical University, Department of Epidemiology and Biostatistics, School of Public Health
**Primary Subject Heading**:	Epidemiology
Secondary Subject Heading:	Cardiovascular medicine, Diabetes and endocrinology, Public health
Keywords:	EPIDEMIOLOGY, PUBLIC HEALTH, Adult cardiology < CARDIOLOGY, General diabetes < DIABETES & ENDOCRINOLOGY

SCHOLARONE™
Manuscripts

BMJ

I, the Submitting Author has the right to grant and does grant on behalf of all authors of the Work (as defined in the below author licence), an exclusive licence and/or a non-exclusive licence for contributions from authors who are: i) UK Crown employees; ii) where BMJ has agreed a CC-BY licence shall apply, and/or iii) in accordance with the terms applicable for US Federal Government officers or employees acting as part of their official duties; on a worldwide, perpetual, irrevocable, royalty-free basis to BMJ Publishing Group Ltd ("BMJ") its licensees and where the relevant Journal is co-owned by BMJ to the co-owners of the Journal, to publish the Work in this journal and any other BMJ products and to exploit all rights, as set out in our licence.

The Submitting Author accepts and understands that any supply made under these terms is made by BMJ to the Submitting Author unless you are acting as an employee on behalf of your employer or a postgraduate student of an affiliated institution which is paying any applicable article publishing charge ("APC") for Open Access articles. Where the Submitting Author wishes to make the Work available on an Open Access basis (and intends to pay the relevant APC), the terms of reuse of such Open Access shall be governed by a Creative Commons licence – details of these licences and which Creative Commons licence will apply to this Work are set out in our licence referred to above.

Other than as permitted in any relevant BMJ Author's Self Archiving Policies, I confirm this Work has not been accepted for publication elsewhere, is not being considered for publication elsewhere and does not duplicate material already published. I confirm all authors consent to publication of this Work and authorise the granting of this licence.

For peer review only

Association of low birth weight with cardiometabolic diseases in the Swedish twins: A population-based cohort study

Xuerui Li[1,2,3], MSc; Rongrong Yang[4], PhD; Wenzhe Yang[1,2,3], Bs; Hui Xu[5], PhD; Ruixue Song[1,2,3], MSc; Xiuying Qi[1,2,3]*, PhD; Weili Xu[1,2,3,6]*, PhD

Affiliations of authors:

[1] Department of Epidemiology and Biostatistics, School of Public Health, Tianjin Medical University, Tianjin, China

[2] Tianjin Key Laboratory of Environment, Nutrition and Public Health, Tianjin, China

[3] Center for International Collaborative Research on Environment, Nutrition and Public Health, Tianjin, China

[4] Public Health Science and Engineering College, Tianjin University of Traditional Chinese Medicine, Tianjin, China

[5] Big Data and Engineering Research Center, Beijing Children's Hospital, Capital Medical University, National Center for Children's Health, Beijing, China

[6] Aging Research Center, Department of Neurobiology, Health Care Sciences and Society, Karolinska Institutet and Stockholm University, Stockholm, Sweden

***Corresponding authors**

Weili Xu & Xiuying Qi, Professors

Dept. of Epidemiology & Biostatistics, School of Public Health

Tianjin Medical University, Tianjin, China

Qixiangtai Road 22, Heping District, 300070, Tianjin, PR, China

Fax number: +86 022-83336727; Phone: +86 022-83336727

Email: xuweili@tmu.edu.cn; qixiuying@tmu.edu.cn

Weili Xu, Associate Professor & Senior Researcher

Aging Research Center, Karolinska Institutet

Tomtebodavägen 18A Floor 10, SE-171 65 Solna, Stockholm, Sweden

Phone: +46 8 524 858 26; Email: weili.xu@ki.se

Word count: Title-100 (no space); Abstract-292; Text only-3564; References-51; Tables-4; Figure-1; Supplementary Tables-10; Supplementary Figure-1.

For peer review only

For peer review only - http://bmjopen.bmj.com/site/about/guidelines.xhtml

Abstract

Objective: To examine the association between low birth weight (LBW) and cardiometabolic diseases (CMDs, including heart disease, stroke, and type 2 diabetes mellitus) in adulthood, and to explore whether genetic, early-life environmental, and healthy lifestyle factors play a role in this association.

Design: A population-based twin study.

Setting: Twins from the Swedish Twin Registry who were born in 1958 or earlier participated in the Screening Across the Lifespan Twin (SALT) study for a full-scale screening during 1998-2002 and were followed until 2014.

Participants: 19,779 twin individuals in Sweden with birth weight data available (mean age: 55.45 years).

Primary and secondary outcome measures: CMDs were assessed based on self-reported medical records, medication use, and records from the National Patient Registry. A lifestyle index encompassing smoking status, alcohol consumption, exercise levels, and body mass index was derived from the SALT survey and categorized as unfavorable, intermediate, or favorable. Data were analyzed using generalized estimating equation (GEE) models and conditional logistic regression models.

Results: Of all participants, 3998 (20.2%) had LBW and 5335 (27.0%) had incident CMDs (mean age at onset: 63.64±13.26 years). In GEE models, the odds ratio (OR, 95% confidence interval [CI]) of any CMD was 1.39 (1.27-1.52) for LBW. In conditional logistic regression models, the LBW-CMDs association became non-significant (OR [95% CI] = 1.21 [0.94-1.56]). The difference in ORs from the two models was statistically significant (P<0.001). In

For peer review only - http://bmjopen.bmj.com/site/about/guidelines.xhtml

the joint effect analysis, the multi-adjusted OR (95% CI) of CMDs was 3.47 (2.72-4.43) for participants with LBW plus an unfavorable lifestyle and 1.25 (0.96-1.62) for those with LBW plus a favorable Lifestyle.

Conclusions: LBW is associated with an increased risk of adult CMDs, and genetic and early-life environmental factors may account for this association. However, a favorable lifestyle profile may modify this risk.

Key words: Population-based twin study; Birth weight; Cardiometabolic disease; Swedish twins; Lifestyle

Strengths and limitations of this study:

- This study provides an extraordinary opportunity to explore the association between low birth weight and cardiometabolic diseases by using a twin study design to control for some unmeasured confounders.
- The investigation into factors that might compensate for the risk effect of low birth weight on cardiometabolic diseases is unique.
- Birth weight was based on self-reports and non-differential misclassification among different birth weight groups could not be ruled out, possibly leading to an underestimation of the observed associations.
- Some prenatal factors (such as gestational age, maternal smoking during pregnancy, or premature birth) could not be controlled for, as information on these factors was not available.
- Potential variations of lifestyle factors during the follow-up also could not be assessed.

For peer review only - http://bmjopen.bmj.com/site/about/guidelines.xhtml

For peer review only

Introduction

With population aging has come an increase in the prevalence of chronic diseases, especially heart diseases (i.e. coronary heart diseases and heart failure), stroke, and type 2 diabetes mellitus (T2DM).[1] According to the World Health Organization (WHO), heart diseases and stroke, so called cardiovascular disease (CVD), is the leading cause of disease burden and death worldwide.[2,3] About 17.6 million deaths were attributed to CVD globally in 2016.[2] Meanwhile, there were 451 million adults living with diabetes worldwide in 2017 (90% of whom had T2DM), and this number is projected to increase to 693 million by 2045.[4,5] All of these co-occurring chronic diseases have been defined as cardiometabolic diseases (CMDs).[6,7]

Recently, beyond the effects of some traditional risk factors including age, smoking, drinking, and body mass index (BMI) on individual CMDs, the role of early-life experiences in the future development of chronic diseases have drawn special attention.[8] Birth weight, an early life indicator,[9] is frequently used to explore the effects of early-life experiences on the risk of individual CMDs in adulthood. Several cohort studies have shown that low birth weight (LBW) is associated with an increased risk of coronary heart disease,[10] stroke,[11] and T2DM,[12,13] but with some inconsistent findings.[14,15] Moreover, many studies have examined the relationship between birth weight and metabolic syndrome with inconsistent results,[16-18] but no studies have investigated the association of LBW with the risk of CMDs.

CMDs are complex genetic and lifestyle-related disorders,[19-21] and birth weight may also be affected by genetic factors and intrauterine environment.[22] However, the role of the genetic and early-life environmental factors (another term for shared environmental factors), such as intrauterine environment and prenatal nutritional status, in the association between birth weight and CMDs remains unclear. Twin studies make it possible to minimize potential confounding effects of unmeasured genetic predisposition and shared early-life environment when comparisons are made between twins.[23,24] Apart from genetic factors, some modifiable

For peer review only - http://bmjopen.bmj.com/site/about/guidelines.xhtml

lifestyle factors such as not smoking, moderate alcohol consumption, engagement in physical activities, and maintaining a healthy weight have been reported to be linked to a lower risk of CVD or T2DM.[25,26] However, previous population-based cohort studies have only shown that healthy lifestyle (such as active physical activity, not smoking, moderate alcohol consumption, and BMI <25) may reduce the risk effect of LBW on the development of diabetes.[27,28] Questions remain regarding whether and to what extent healthy lifestyle may mitigate the risk of LBW on CMDs more widely.

In the present study, we aimed to 1) verify the relationship between LBW and risk of CMDs using population-based Sweden twin data and 2) explore whether genetic, early-life environmental, and healthy lifestyle factors play a role in this association.

Methods

Study population

This prospective, nested case-control study included twins from the nationwide Swedish Twin Registry (STR), which started in the 1960s.[29] From 1998 to 2002, all living twins born in 1958 or earlier were recruited to participate in the Screening Across the Lifespan Twin (SALT) study, a full-scale screening through a computer-assisted telephone interview. Of the 19,940 twin individuals in the SALT study with birth weight data available, we excluded 53 individuals with birth weights that were outliers (extreme values; i.e., birth weight ≤300 g or ≥4520 g) to minimize possible misclassification and 108 individuals with type 1 diabetes. Finally, 19,779 individuals were included in the current study (Supplemental Figure S1).

Data collection

Data on age, sex, educational attainment, marital status, and zygosity status were collected through the SALT survey.[29] Zygosity status was categorized as monozygotic, dizygotic, or undetermined zygosity on the basis of self-reported information about childhood resemblance,

For peer review only - http://bmjopen.bmj.com/site/about/guidelines.xhtml

which was validated against biological markers with 95–99% accuracy.[29] Education was dichotomized into <8 vs. ≥8 years according to the number of years of formal schooling attained. Marital status was classified into married/cohabitating vs. single (including divorced or widows/widowers).

Information on medical conditions including heart disease, stroke, T2DM, and hypertension was derived from the National Patient Registry (NPR), which covers all inpatient diagnoses in Sweden from the 1960s and outpatient (specialist clinic) diagnoses from 2001 until 2014.[30] Each medical record in the NPR included up to eight discharge diagnoses according to the International Classification of Disease (ICD) codes. The seventh revision (ICD-7) was used through 1968, the eighth revision (ICD-8) from 1969 to 1986, the ninth revision (ICD-9) from 1987 till 1996, and the tenth revision (ICD-10) from 1997 through the end of 2014.

Informed consent was acquired from all participants. Data collection procedures were approved by the Regional Ethics Committee at Karolinska Institutet, Stockholm, Sweden and the Institutional Review Board of the University of Southern California, USA.

Assessment of birth weight

Data on birth weight was collected based on self-reports from SALT or STR. Generally, LBW was defined as birth weight <2500g in singletons.[31] However, twins may experience a more unfavorable intrauterine environment, causing them to have a lower birth weight (on average 800g) than singletons.[32] Thus, birth weight in the present study was categorized as <2.0 kg (LBW), 2.0-3.0 kg (moderate birth weight [MBW]), or >3.0kg (high birth weight [HBW])[32] considering its distribution.

Ascertainment of CMD

In the current analysis, CMDs included heart disease (coronary heart disease and heart failure), stroke (ischemic stroke and hemorrhagic stroke), and T2DM, all of which were

For peer review only - http://bmjopen.bmj.com/site/about/guidelines.xhtml

diagnosed based on self-reported medical records, medication use, and NPR data. The detailed ICD codes for each disease were shown in the Supplemental Table S1.

CMD status was categorized as CMD-free and any CMD (i.e., presence any of heart disease, stroke, and/or T2DM). The any CMD group was further classified as only one CMD (heart disease, or stroke, or T2DM), any two CMDs (any two of the following: heart disease, stroke, and T2DM), and three or more CMDs (heart disease, stroke, and T2DM).

Assessment of lifestyle-related factors

Information on lifestyle factors (including smoking status, alcohol consumption, physical exercise, and BMI) was obtained from the SALT survey. In detail, smoking status was dichotomized as non-smoking vs. former/current smoker. Alcohol consumption was categorized as no/mild drinking vs. heavy drinking based on the survey question asking whether participants have ever drunk excessively over a period. Data on physical exercise was collected by a question on average exercise with seven response options: I) "almost never," II) "much less than average," III) "less than average," IV) "average," V) "more than average," VI) "much more than average," and VII) "maximum",[33] and was dichotomized as "inactive" including the first four groups (I-IV) and "active" including the last three groups (V-VII). BMI in adulthood (mean age 55.45±9.05) was calculated as weight (kg) divided by squared height (m^2) and classified as underweight (<18.5), normal weight (18.5-24.9), overweight (25-29.9), and obesity (≥30) according to the WHO classification. Obesity was merged with overweight (hereafter overweight; that is, BMI ≥25), and underweight was merged with normal weight as non-overweight (BMI <25).

In the current study, on the basis of the data availability, the following four factors were considered as healthy lifestyle factors: 1) non-smoking; 2) no/mild alcohol consumption; 3) active physical exercise; 4) non-overweight in adulthood.[34] The four factors were combined into a lifestyle index with a score ranging from 0-4, with 1 point representing each factor.

For peer review only - http://bmjopen.bmj.com/site/about/guidelines.xhtml

Participants were categorized according to their score of lifestyle index: 1) unfavorable (0-1): participants who had no healthy lifestyle factors or only one; 2) intermediate (2-3): those who had two or three healthy lifestyle factors; 3) favorable (4): those who had all the healthy lifestyle factors.

Statistical analyses

The characteristics of participants in different groups were compared using Chi-square tests for categorical variables and one-way analysis of variance/Kruskal-Wallis H test for continuous variables. Missing values on education level (n=92), smoking status (n=77), alcohol consumption (n=117), marital status (n=2), physical exercise (n=1179), and BMI (n=290) were imputed using Rubin's rule for pooling estimates to obtain valid statistical inferences.[24]

In our study, two analytical strategies were applied. First, generalized estimating equation (GEE) models were used for unmatched case-control analysis. GEE models are conceptually equivalent to logistic regression for the analysis of classic case-control design but control for the clustering of twins within a pair. Second, conditional logistic regression models were used for cotwin matched case-control analysis using a pair of twins that was discordant for the outcome. Cotwin matched design (especially in monozygotic twins) appeared more informative since cases and controls were comparable with respect to genetic background and early-life environmental factors such as intrauterine environment, prenatal and postnatal nutritional status, and childhood socioeconomic status.[35,36] In both GEE and conditional logistic regression, the odds ratios (ORs) and 95% confidence intervals (CIs) were estimated for the association between birth weight (reference: MBW) and CMDs. Logistic regression was used to test the difference in ORs from GEE and conditional logistic regression models by examining the difference in the proportions of birth weight between unmatched controls and co-twin matched controls.[36] If an OR for the observed association becomes strengthened

For peer review only - http://bmjopen.bmj.com/site/about/guidelines.xhtml

or attenuated (or even disappears) in co-twin control analyses compared with that in the unmatched case-control analysis, and the difference in ORs from the two models is significant, then genetic and/or early-life environmental factors are likely to play a role in the association.[24,35,37] If the ORs are similar between the two models without a statistically significant difference, then the effect of genetic and/or early-life environmental factors in the association can be neglected.[23,36] We hypothesized that LBW would be a significant risk factor for CMDs in a classical case-control analysis, but that the association between LBW and CMDs would be attenuated in the cotwin-matched analysis after controlling for genetic, maternal, and environmental factors shared by twins. Logistic regression was used to test the difference in ORs from the GEE model and conditional logistic regression.

Considering information on lifestyle factors was obtained from the SALT questionnaire during 1998-2002, we excluded 1748 participants who developed CMDs before the SALT recruitment, and thus 18,031 participants remained for the joint effect analysis. The combined effect of the LBW (no vs. yes) and lifestyle index (unfavorable/intermediate/favorable) on the risk of CMDs was assessed by creating dummy variables based on the joint exposures to both factors. The presence of an additive interaction was examined by estimating relative excess risk due to interaction (RERI), the attributable proportion (AP), and the synergy index (S).

All the models were basic adjusted for age, sex, and education, and further adjusted for smoking, alcohol consumption, marital status, physical exercise, BMI, and hypertension. *P*-values less than 0.05 were considered statistically significant. All statistical analyses were performed using SAS statistical software version 9.4 (SAS institute, Cary, NC) and IBM SPSS Statistics 20.0 (IBM Corp, New York, NY).

Patient and public involvement

Patients and the public were not involved in the design, or conduct, or reporting of this study.

For peer review only - http://bmjopen.bmj.com/site/about/guidelines.xhtml

Results

Characteristics of the study population

Among all participants (n=19,779), 3998 (20.2%) had LBW. The average age at recruitment was 55.45 (±9.05) years. Compared with MBW individuals, those with LBW were more likely to be older, male, monozygotic twins, single, have lower education, have higher BMI, be physically inactive, and have hypertension. Participants who had HBW were more likely to be male, dizygotic twins, smokers, heavy drinkers, and have higher BMI (Table 1).

(Insert Table 1 here)

Association between birth weight and CMDs in unmatched case-control analysis

In the multi-adjusted GEE model, compared to participants with MBW, those with LBW had a significantly higher risk of coronary heart disease, heart failure, ischemic stroke, and T2DM, which were further combined as CMDs (n=5335), as shown in Table 2. LBW was associated with an increased risk of any CMD (OR 1.39, 95% CI 1.27-1.52). However, HBW was not significantly associated with any CMDs (OR 1.05, 95% CI: 0.96-1.16). Therefore, MBW and HBW were combined into non-LBW group as reference in the following analysis.

(Insert Table 2 here)

Compared to non-LBW, the OR (95% CI) for the association between LBW and any CMD was 1.37 (1.25-1.50). The multi-adjusted ORs (95% CIs) of LBW were 1.28 (1.17-1.41) for only one CMD, 1.48 (1.28-1.72) for any two CMDs, and 1.82 (1.37-2.42) for three or more CMDs (reference: CMD-free), indicating the LBW-CMDs risk became higher when multiple CMDs were co-occurring (P for trend <0.001) (Supplemental Table S2). Further, the OR (95% CI) of the birth weight-CMDs association was 0.84 (0.80-0.89) when birth weight was used as a continuous variable, suggesting a dose-dependent relationship between greater birth weight and lower CMDs risk (Supplemental Table S3).

Association between LBW and CMDs in co-twin matched case-control analysis

For peer review only - http://bmjopen.bmj.com/site/about/guidelines.xhtml

In the co-twin matched case-control analysis consisting of 845 dizygotic pairs and 290 monozygotic pairs, the association between LBW and any CMD was attenuated compared to the GEE model and became non-significant (OR: 1.21, 95% CI 0.94-1.56). The ORs (95% CIs) for the associations were 1.34 (0.96-1.89) in dizygotic pairs and 1.07 (0.66-1.73) in monozygotic pairs (Table 3).

The difference in ORs from the GEE model vs. conditional logistic model was statistically significant (OR 1.39, 95% CI 1.21-1.59, $P<0.001$), which suggested that genetic and early-life environment factors might play an important role in LBW-CMDs association.

(Insert Table 3 here)

Association between lifestyle-related factors and CMDs

In basic- and multi-adjusted GEE models, not smoking, no/moderate alcohol drinking, active physical exercise, and being non-overweight were individually related to a decreased risk of any CMD. When combined as a lifestyle index (unfavorable, intermediate, and favorable), compared to an unfavorable lifestyle profile, an intermediate and a favorable lifestyle profile were significantly associated with a lower risk of any CMD, ORs (95% CIs) were 0.62 (0.55-0.69) and 0.40 (0.35-0.47), respectively (Table 4).

(Insert Table 4 here)

Joint effect of LBW and healthy lifestyle factors on CMD risk

In the joint effect analysis, the multi-adjusted ORs (95% CIs) of any CMDs were 1.25 (0.96-1.62) for participants with LBW plus a favorable lifestyle profile, 1.94 (1.64-2.28) for those with LBW plus an intermediate lifestyle profile, and 3.47 (2.72-4.43) for those with LBW plus an unfavorable lifestyle profile (reference: those with non-LBW plus a favorable lifestyle profile) (Figure 1 and Supplemental Table S4).

The additive interaction between the unfavorable lifestyle profile and LBW on CMDs was statistically significant (AP 0.199, 95% CI 0.016-0.381, $P=0.03$; S 1.506, 1.001-2.267,

For peer review only - http://bmjopen.bmj.com/site/about/guidelines.xhtml

P<0.001), indicating that if people with LBW have a favorable or intermediate lifestyle, the risk of LBW on CMDs can be reduced by 20% (Supplemental Table S5).

(Insert Figure 1 here)

Supplementary analysis

The results were not much altered compared to those from the initial analysis when we repeated the following analyses after: 1) stratifying by sex to address possible sex differences in the CMDs[38] (Supplemental Table S6), 2) additionally adjusting for survival status considering the association between LBW and mortality[39] (Supplemental Table S7), 3) excluding participants who developed CMDs before SALT recruitment (n=1748) (Supplemental Table S8), 4) excluding participants with missing values for covariates (n=1430) (Supplemental Table S9), and 5) stratifying by twin birth weight concordance and discordance (Supplemental Table S10).

Discussion

In this large-scale, prospective, population-based nested case-control study of Swedish twins, we found that: 1) LBW was associated with an increased risk of CMDs including coronary heart disease, heart failure, ischemic stroke, and T2DM in adulthood, and the risk became higher when multiple CMDs were co-occurring; 2) Genetic background and early-life environmental factors appear to account for the LBW-CMDs association; 3) A favorable lifestyle profile may modify the risk effect of LBW on CMDs.

Over the past two decades, the relationship between birth weight and T2DM[12,13,40] has been well documented. However, reports have been inconsistent regarding the association between birth weight and coronary heart disease. Three cohort studies have reported a relationship between LBW and the risk of coronary heart disease.[10,11,41] By contrast, Banci et al. found that higher birth weight was associated with a higher risk of coronary heart disease.[14] Another

For peer review only - http://bmjopen.bmj.com/site/about/guidelines.xhtml

study showed there was no relationship between birth weight and coronary heart disease.[15] In addition, evidence on the relationship between LBW and heart failure or ischemic stroke is sparse. To our knowledge, no studies have investigated the association of LBW with the risk of CMDs. In the present study, we found that LBW was associated with about 10-40% increased risk of coronary heart disease, heart failure, ischemic stroke (not hemorrhagic stroke), and T2DM. Further, we examined the relationship between birth weight and the risk of combined CMDs and found that individuals with LBW had an almost 40% higher risk of any CMD compared to those with non-LBW.

The potential contribution of genetic susceptibility and early-life environmental factors to the LBW-CMDs association is still unclear. Previous twin cohort studies have shown that LBW is associated with an increased risk of CVD and T2DM when twins were considered as independent individuals. This association only held in outcome-discordant dizygotic twins but not in monozygotic twin pairs, suggesting that genetic mechanisms played a role in this association.[13,32,42] In the present study, we found that the LBW-CMDs association became non-significant in both dizygotic and monozygotic twin pairs by using co-twin matched analyses. These results illustrated that early-life environmental factors could play an important role in the association between LBW and subsequent CMDs, along with genetic background.

Modifiable lifestyle factors (such as smoking, drinking, physical exercise, and BMI) deserve to be studied in the context of the LBW-CMDs association. To date, only a few studies have investigated the joint effect of LBW with lifestyle factors on T2DM.[27,28,43] One of the studies included 149,794 participants from three large prospective cohorts and showed that LBW and unhealthy adulthood lifestyles encompassing smoking, non-moderate alcohol consumption, lower exercise intensity, and BMI ≥25 were jointly related to an increased risk of T2DM.[28] Another cohort study indicated that the risk of diabetes associated with LBW

For peer review only - http://bmjopen.bmj.com/site/about/guidelines.xhtml

could be eliminated in those with a high physical activity level,[27] and individuals predisposed to T2DM due to LBW could be protected from glucose intolerance by regular exercise.[43] However, no study has illustrated the joint effect of LBW and healthy lifestyle on subsequent CMDs. In the present study, we found that people with LBW and an intermediate or a favorable lifestyle profile (including not smoking, no/mild alcohol consumption, active physical exercise, and being non-overweight) had a significantly lower risk of CMDs than those who had LBW and unfavorable lifestyle profile. To our knowledge, this is the first study to provide evidence that a healthy lifestyle might compensate for the risk effect of LBW on CMDs.

Several mechanisms may explain the relationship between LBW and the risk of CMDs. The "fetal origins hypothesis" proposes that fetal malnutrition in middle to late gestation may generate a compensatory "survival" mechanism to redirect scant energy supplies from muscle to vital tissues, causing permanent alterations in physiology, metabolism, and structure.[44,45] However, the risk of preterm birth in twins is significantly higher than singletons.[46] Furthermore, a preterm fetus with LBW may also have appropriate fetal growth, especially for twins. Thus, among twins, birth weight may not reflect the actual growth restriction of the fetus. This may explain some of the contradictions in the relationship between LBW and adult chronic disease. Additionally, some genes (such as insulin class I allele or variants of mitochondrial DNA) have been associated with both birth weight loss and insulin resistance.[47,48] All of these alterations could result in an increased risk of CVD and T2DM in adulthood. Moreover, a haplotype of the glucocorticoid receptor gene may modify the association between size at birth and glucose tolerance.[49] However, maintaining a healthy lifestyle in adulthood may mitigate the risk of CMDs by improving insulin sensitivity and body composition, as well as controlling glycemic, blood pressure, and lipid profile.[50]

Strengths and Limitations

For peer review only - http://bmjopen.bmj.com/site/about/guidelines.xhtml

Notable strengths of our study involve the large nationwide population-based twin cohort, which provided an extraordinary opportunity to explore the association between LBW and the risk of CMDs in adulthood by controlling for some unmeasured confounders, such as genetic background and early-life environmental factors. Furthermore, our investigation of potential compensatory factors against the LBW-CMDs association is unique. Nevertheless, some limitations should be pointed out. First, hypertension was defined only based on self-reported data from the NPR, and subjects with undiagnosed hypertension might have been misclassified as hypertension-free. Thus, hypertension was not categorized as a CMD in the current study. Second, the assessment of birth weight was based on self-report so potential information bias could not be ruled out. However, such bias is more likely to be non-differential misclassification resulting in underestimation for the given associations. Third, data on some prenatal factors (such as gestational age, maternal smoking during pregnancy, or premature birth) and parental socioeconomic status were not available and could not be fully controlled for. In addition, potential variations in lifestyle factors during follow-up could not be assessed. Fourth, diet could be partially taken into account, as it is closely associated with other lifestyle factors such as smoking, alcohol consumption, physical exercise, and BMI.[51] However, data on diet was not available in the SALT study. Finally, LBW in this study was defined as <2.0 kg in twins. Caution is needed when generalizing our findings to other populations.

Conclusion

This study provides evidence that LBW is associated with increased risk of CMDs including coronary heart disease, heart failure, ischemic stroke, and T2DM. The risk of CMDs related to LBW tends to increase with the number of co-occurring CMDs. Further, genetic and early-life environmental factors play an important role in the LBW-CMDs association. However, a

For peer review only - http://bmjopen.bmj.com/site/about/guidelines.xhtml

favorable lifestyle involving not smoking, no/mild alcohol consumption, active physical exercise, and a BMI<25 may compensate for the risk effect of LBW on CMDs. Our findings highlight the need for monitoring and controlling LBW for the prevention of CMDs, and the importance of maintaining a favorable lifestyle profile in people with LBW in adulthood to reduce the risk of CMDs.

For peer review only

For peer review only - http://bmjopen.bmj.com/site/about/guidelines.xhtml

Acknowledgements

We would like to thank the Swedish Twin Registry for access to the data, and we are grateful to all the twins who took part in the study, as well as the members of the survey teams. We are grateful to Prof. Nancy L. Pedersen for her great contribution to the design of the twin data collection. The Swedish Twin Registry is managed by Karolinska Institutet and receives funding through the Swedish Research Council under the grant No. 2017-00641.

Contributors

WX conceptualized and designed the study. XL conducted the literature search, analyzed the data, and wrote the first draft. XL, RY, WY, HX, RS, XQ, and WX contributed to the discussion and interpretation of the results. WX and XQ were involved in study supervision. All authors contributed to critical revision of the manuscript for important intellectual content and gave their final approval of the version to be published. WX obtained funding for the study. XL and WX had full access to all the data in the study and take responsibility for the integrity of the data and the accuracy of the data analysis.

Funding

This work was supported by grants from the Swedish Research Council (No. 2017-00981), the National Natural Science Foundation of China (No. 81771519), the Konung Gustaf V:s och Drottning Victorias Frimurare Foundation (No. 2016-2020), Demensfonden, Strokefonden, Cornells Stiftelse and Alzheimerfonden (2018-2019). This project is part of CoSTREAM (www.costream.eu) and received funding from the European Union's Horizon 2020 research and innovation program under grant agreement No 667375. The funders had no role in the design and conduct of the study; collection, management, analysis, and interpretation of the data; preparation, review, or approval of the manuscript; and decision to submit the manuscript for publication.

Competing interests

For peer review only

For peer review only - http://bmjopen.bmj.com/site/about/guidelines.xhtml

There are no competing interests for any author.

Patient consent for publication

Not applicable.

Ethics approval

The approval from the Ethics Committee of the Karolinska Institutet was obtained (Dnr: 97: 051)

Data availability statement

Data are available upon reasonable request.

For peer review only

For peer review only - http://bmjopen.bmj.com/site/about/guidelines.xhtml

Reference

1. Kivimäki M, Kuosma E, Ferrie JE*, et al.* Overweight, obesity, and risk of cardiometabolic multimorbidity: pooled analysis of individual-level data for 120 813 adults from 16 cohort studies from the USA and Europe. *Lancet Public Health* 2017;2:e277-85.

2. Benjamin EJ, Muntner P, Alonso A*, et al.* Heart Disease and Stroke Statistics-2019 Update: A Report From the American Heart Association. *Circulation* 2019;139:e56-528.

3. Disease GBD, Injury I, Prevalence C. Global, regional, and national incidence, prevalence, and years lived with disability for 328 diseases and injuries for 195 countries, 1990-2016: a systematic analysis for the Global Burden of Disease Study 2016. *Lancet* 2017;390:1211-59.

4. Chatterjee S, Khunti K, Davies M. Type 2 diabetes. *Lancet* 2017;389:2239-2251.

5. Cho NH, Shaw JE, Karuranga S*, et al.* IDF Diabetes Atlas: Global estimates of diabetes prevalence for 2017 and projections for 2045. *Diabetes Res Clin Pract* 2018;138:271-81.

6. Wang Z, Marseglia A, Shang Y*, et al.* Leisure activity and social integration mitigate the risk of dementia related to cardiometabolic diseases: A population-based longitudinal study. *Alzheimers Dement* 2020;16:316-25.

7. Keenan T, Zhao W, Rasheed A*, et al.* Causal Assessment of Serum Urate Levels in Cardiometabolic Diseases Through a Mendelian Randomization Study. *J Am Coll Cardiol* 2016;67:407-16.

8. Smith CJ, Ryckman KK, Barnabei VM*, et al.* The impact of birth weight on cardiovascular disease risk in the Women's Health Initiative. *Nutr Metab Cardiovasc Dis* 2016;26:239-45.

For peer review only - http://bmjopen.bmj.com/site/about/guidelines.xhtml

9. Lawani SO, Demerath EW, Lopez FL*, et al.* Birth weight and the risk of atrial fibrillation in whites and African Americans: the Atherosclerosis Risk In Communities (ARIC) study. *BMC Cardiovasc Disord* 2014;14:69.

10. Rich-Edwards JW, Kleinman K, Michels KB*, et al.* Longitudinal study of birth weight and adult body mass index in predicting risk of coronary heart disease and stroke in women. *BMJ* 2005;330:1115.

11. Lawlor DA, Ronalds G, Clark H*, et al.* Birth weight is inversely associated with incident coronary heart disease and stroke among individuals born in the 1950s: findings from the Aberdeen Children of the 1950s prospective cohort study. *Circulation* 2005;112:1414-8.

12. Whincup PH, Kaye SJ, Owen CG*, et al.* Birth Weight and Risk of Type 2 Diabetes. *JAMA* 2008;300:2886.

13. Johansson S, Iliadou A, Bergvall N*, et al.* The association between low birth weight and type 2 diabetes: contribution of genetic factors. *Epidemiology* 2008;19:659-65.

14. Banci M, Saccucci P, Dofcaci A*, et al.* Birth weight and coronary artery disease. The effect of gender and diabetes. *Int J Biol Sci* 2009;5:244-8.

15. Morley R, McCalman J, Carlin JB. Birthweight and coronary heart disease in a cohort born 1857-1900 in Melbourne, Australia. *Int J Epidemiol* 2006;35:880-5.

16. Xiao X, Zhang ZX, Li WH*, et al.* Low birth weight is associated with components of the metabolic syndrome. *Metabolism* 2010;59:1282-6.

17. dos Santos Alves Pde J, P T Henriquesa AC, Pinto LR*, et al.* Endothelial and metabolic disorders in adolescence: low birth weight is not an isolated risk factor. *J Pediatr Endocrinol Metab* 2015;28:407-13.

18. Briskiewicz BL, Barreto SM, do Amaral JF*, et al.* Early-life nutritional status and metabolic syndrome: gender-specific associations from a cross-sectional analysis of

For peer review only - http://bmjopen.bmj.com/site/about/guidelines.xhtml

the Brazilian Longitudinal Study of Adult Health (ELSA-Brasil). *Public Health Nutr* 2018;21:1546-53.

19. Marenberg ME, Risch N, Berkman LF, *et al.* Genetic susceptibility to death from coronary heart disease in a study of twins. *N Engl J Med* 1994;330:1041-6.

20. Almgren P, Lehtovirta M, Isomaa B, *et al.* Heritability and familiality of type 2 diabetes and related quantitative traits in the Botnia Study. *Diabetologia* 2011;54:2811-9.

21. Bak S, Gaist D, Sindrup SH, *et al.* Genetic liability in stroke: a long-term follow-up study of Danish twins. *Stroke* 2002;33:769-74.

22. Lunde A, Melve KK, Gjessing HK, *et al.* Genetic and environmental influences on birth weight, birth length, head circumference, and gestational age by use of population-based parent-offspring data. *Am J Epidemiol* 2007;165:734-41.

23. Xu W, Qiu C, Gatz M, *et al.* Mid- and late-life diabetes in relation to the risk of dementia: a population-based twin study. *Diabetes* 2009;58:71-7.

24. Yang R, Pedersen NL, Bao C, *et al.* Type 2 diabetes in midlife and risk of cerebrovascular disease in late life: a prospective nested case-control study in a nationwide Swedish twin cohort. *Diabetologia* 2019;62:1403-11.

25. Lv J, Yu C, Guo Y, *et al.* Adherence to Healthy Lifestyle and Cardiovascular Diseases in the Chinese Population. *J Am Coll Cardiol* 2017;69:1116-25.

26. Lv J, Yu C, Guo Y, *et al.* Adherence to a healthy lifestyle and the risk of type 2 diabetes in Chinese adults. *Int J Epidemiol* 2017;46:1410-20.

27. Hu CY, Mu YM, Wan Q, *et al.* Association between birth weight and diabetes: Role of body mass index and lifestyle in later life. *Journal of Diabetes* 2020;12:10-20.

28. Li Y, Ley SH, Tobias DK, *et al.* Birth weight and later life adherence to unhealthy lifestyles in predicting type 2 diabetes: prospective cohort study. *BMJ*

For peer review only

For peer review only - http://bmjopen.bmj.com/site/about/guidelines.xhtml

2015;351:h3672.

29. Lichtenstein P, De Faire U, Floderus B*, et al.* The Swedish Twin Registry: a unique resource for clinical, epidemiological and genetic studies. *J Intern Med* 2002;252:184-205.

30. Patientregistret - Socialstyrelsen n.d. https://www.socialstyrelsen.se/statistik-och-data/register/alla-register/patientregistret/ Accessed Octorber 20 2020.

31. Xiao X, Zhang ZX, Cohen HJ*, et al.* Evidence of a Relationship Between Infant Birth Weight and Later Diabetes and Impaired Glucose Regulation in a Chinese Population. *Diabetes Care* 2007;31:483-7.

32. Hubinette A, Cnattingius S, Johansson ALV*, et al.* Birth weight and risk of angina pectoris: Analysis in Swedish twins. *Eur J Epidemiol* 2002;18:539-44.

33. Carlsson S, Ahlbom A, Lichtenstein P*, et al.* Shared genetic influence of BMI, physical activity and type 2 diabetes: a twin study. *Diabetologia* 2013;56:1031-5.

34. Nyberg ST, Singh-Manoux A, Pentti J*, et al.* Association of Healthy Lifestyle With Years Lived Without Major Chronic Diseases. *JAMA Intern Med* 2020;180:760-8.

35. Kato K, Sullivan PF, Evengard B*, et al.* Premorbid predictors of chronic fatigue. *Arch Gen Psychiatry* 2006;63:1267-72.

36. Xu WL, Atti AR, Gatz M*, et al.* Midlife overweight and obesity increase late-life dementia risk: a population-based twin study. *Neurology* 2011;76:1568-74.

37. Bao C, Yang R, Pedersen NL*, et al.* Overweight in midlife and risk of cancer in late life: A nationwide Swedish twin study. *Int J Cancer* 2019;144:2128-34.

38. Kuznetsova T. Sex Differences in Epidemiology of Cardiac and Vascular Disease. *Adv Exp Med Biol* 2018;1065:61-70.

39. Hoy WE, Nicol JL. The Barker hypothesis confirmed: association of low birth weight with all-cause natural deaths in young adult life in a remote Australian Aboriginal

For peer review only - http://bmjopen.bmj.com/site/about/guidelines.xhtml

community. *J Dev Orig Health Dis* 2019;10:55-62.

40. Kaijser M, Bonamy AK, Akre O*, et al.* Perinatal risk factors for diabetes in later life. *Diabetes* 2009;58:523-6.

41. Andersen LG, Angquist L, Eriksson JG*, et al.* Birth weight, childhood body mass index and risk of coronary heart disease in adults: combined historical cohort studies. *PLoS One* 2010;5:e14126.

42. Oberg S, Cnattingius S, Sandin S*, et al.* Birth weight predicts risk of cardiovascular disease within dizygotic but not monozygotic twin pairs: a large population-based co-twin-control study. *Circulation* 2011;123:2792-8.

43. Eriksson JG, Yliharsila H, Forsen T*, et al.* Exercise protects against glucose intolerance in individuals with a small body size at birth. *Prev Med* 2004;39:164-7.

44. Barker DJ. Fetal origins of coronary heart disease. *BMJ* 1995;311:171-4.

45. Poulsen P, Vaag A. Glucose and insulin metabolism in twins: influence of zygosity and birth weight. *Twin Res* 2001;4:350-5.

46. Elliott JP, Istwan NB, Collins A*, et al.* Indicated and non-indicated preterm delivery in twin gestations: impact on neonatal outcome and cost. *J Perinatol* 2005;25:4-7.

47. Dunger DB, Ong KK, Huxtable SJ*, et al.* Association of the INS VNTR with size at birth. ALSPAC Study Team. Avon Longitudinal Study of Pregnancy and Childhood. *Nat Genet* 1998;19:98-100.

48. Casteels K, Ong K, Phillips D*, et al.* Mitochondrial 16189 variant, thinness at birth, and type-2 diabetes. *Lancet* 1999;353:1499-500.

49. Rautanen A, Eriksson JG, Kere J*, et al.* Associations of body size at birth with late-life cortisol concentrations and glucose tolerance are modified by haplotypes of the glucocorticoid receptor gene. *J Clin Endocrinol Metab* 2006;91:4544-51.

50. Marwick TH, Hordern MD, Miller T*, et al.* Exercise training for type 2 diabetes

For peer review only - http://bmjopen.bmj.com/site/about/guidelines.xhtml

mellitus: impact on cardiovascular risk: a scientific statement from the American Heart Association. *Circulation* 2009;119:3244-62.

51. Fransen HP, Boer JMA, Beulens JWJ*, et al.* Associations between lifestyle factors and an unhealthy diet. *Eur J Public Health* 2017;27:274-8.

For peer review only

For peer review only - http://bmjopen.bmj.com/site/about/guidelines.xhtml

Table 1. Characteristics of the study population (n=19779) by birth weight

Characteristics	<2.0 kg n = 3998	2.0-3.0 kg n = 11510	>3.0 kg n = 4271	*P*-value
Age (years), mean (SD)	57.37 (9.6)	55.07 (8.8)	54.70 (8.9)	<0.001
Male sex, n (%)	1307 (32.7)	3504 (30.4)	2042 (47.8)	<0.001
Education, n (%)				
<8 years	1251 (31.3)	2850 (24.8)	1009 (23.6)	<0.001
≥8 years	2747 (68.7)	8660 (75.2)	3262 (76.4)	
Marital status, n (%)				
Married/cohabited	2911 (72.8)	8749 (76.0)	3298 (77.2)	<0.001
Single	1087 (27.2)	2761 (24.0)	973 (22.8)	
Zygosity, n (%)				
Monozygosity	1027 (25.7)	2647 (23.0)	685 (16.0)	<0.001
Dizygosity	2384 (59.6)	7436 (64.6)	3021 (70.7)	
Undetermined	587 (14.7)	1427 (12.4)	565 (13.2)	
BMI, mean (SD)	25.02 (3.8)	24.67 (3.5)	25.13 (3.5)	<0.001
BMI, n (%)				
<18.5 (Underweight)	71 (1.8)	167 (1.4)	46 (1.1)	<0.001
18.5-24.9 (Normal weight)	2108 (52.7)	6600 (57.3)	2218 (52.0)	
25.0-29.9 (Overweight)	1439 (36.0)	3874 (33.7)	1623 (38.0)	
≥30 (Obese)	380 (9.5)	869 (7.6)	384 (9.0)	
Smoking status, n (%)				
Never smoked	2049 (51.2)	5825 (50.6)	1932 (45.2)	<0.001
Former/current smoker	1949 (48.8)	5685 (49.4)	2339 (54.8)	
Alcohol consumption, n (%)				
No/mild drinking	3735 (93.4)	10746 (93.4)	3884 (90.9)	<0.001
Heavy drinking	263 (6.6)	764 (6.6)	387 (9.1)	
Active physical exercise, n (%)				
No	2092 (52.3)	5736(49.8)	2101 (49.2)	0.008
Yes	1905 (48.2)	5774 (50.2)	2170 (50.8)	
Hypertension, n (%)	1299 (33.5)	2954 (25.7)	1023 (24.0)	<0.001

Data were presented as means ± standard deviations or number (%).

Abbreviations: BMI, body mass index; SD, standard deviation.

For peer review only

For peer review only - http://bmjopen.bmj.com/site/about/guidelines.xhtml

Table 2. Odds ratios (ORs) and 95% confidence intervals (CIs) of birth weight in relation to different subtypes of heart diseases, stroke, and diabetes in adulthood: results from Generalized Estimating Equation

Single/combined CMDs	No. of Cases	OR (95% CI) *	OR (95% CI) †
Subtypes of Heart disease			
CHD			
<2.0	622	1.33 (1.19-1.49)	1.27 (1.14-1.43)
2.0-3.0	1166	Reference	Reference
>3.0	497	1.07 (0.95-1.20)	1.08 (0.95-1.22)
HF			
<2.0	214	1.36 (1.13-1.63)	1.27 (1.05-1.53)
2.0-3.0	356	Reference	Reference
>3.0	143	1.13 (0.93-1.39)	1.12 (0.91-1.38)
Subtypes of Stroke			
IS			
<2.0	432	1.20 (1.06-1.36)	1.14 (1.01-1.30)
2.0-3.0	874	Reference	Reference
>3.0	352	1.10 (0.96-1.26)	1.12 (0.98-1.29)
HS			
<2.0	74	1.14 (0.86-1.50)	1.09 (0.82-1.44)
2.0-3.0	162	Reference	Reference
>3.0	59	0.97 (0.72-1.32)	0.99 (0.73-1.34)
T2DM			
<2.0	668	1.45 (1.30-1.61)	1.39 (1.24-1.55)
2.0-3.0	1219	Reference	Reference
>3.0	424	0.88 (0.78-0.99)	0.82 (0.72-0.93)
Any CMDs (CHD, HF, IS, T2DM)			
<2.0	1423	1.44 (1.32-1.57)	1.39 (1.27-1.52)
2.0-3.0	2797	Reference	Reference
>3.0	1115	1.06 (0.97-1.16)	1.05 (0.96-1.16)

Abbreviations: CHD, coronary heart disease; CMDs, cardiometabolic diseases; HF, heart failure; HS, hemorrhagic stroke; IS, Ischemic stroke; T2DM, type 2 diabetes mellitus.

* Adjusted for age, sex, and education.

† Adjusted for age, sex, education, body mass index, smoking, alcohol consumption, marital status, physical exercise, and hypertension.

For peer review only

For peer review only - http://bmjopen.bmj.com/site/about/guidelines.xhtml

Table 3. Odds ratios (ORs) and 95% confidence intervals (CIs) for the association between LBW and adult CMDs in co-twin control analysis using CMDs discordant twin pairs: results from conditional logistic regression

**Co-twin control**	**Co-twin with CMDs**					
	**All zygosity twins** * **(n=1293 pairs)**		**Dizygotic only (n=845 pairs)**		**Monozygotic only (n=290 pairs)**	
	Non-LBW	LBW	Non-LBW	LBW	Non-LBW	LBW
Non-LBW	804	177	549	106	162	46
LBW	153	159	90	100	45	37
Basic-adjusted OR (95% CI) †	1.20 (0.96-1.49)		1.25 (0.94-1.67)		1.03 (0.68-1.56)	
Multi-adjusted OR (95% CI) ‡	1.21 (0.94-1.56)		1.34 (0.96-1.89)		1.07 (0.66-1.73)	

Abbreviations: CMDs, cardiometabolic diseases; LBW, low birth weight.

* Contain 158 pairs of undetermined zygosity twins

† Adjusted for sex and education.

‡ Adjusted for sex, education, body mass index, smoking, alcohol consumption, marital status, physical exercise, and hypertension.

For peer review only

For peer review only - http://bmjopen.bmj.com/site/about/guidelines.xhtml

Table 4. Odds ratios (ORs) and 95% confidence intervals (CIs) of smoking, alcohol consumption, physical exercise, and body mass index (BMI) related to cardiometabolic diseases from Generalized Estimating Equation models

Lifestyle factors	No. of Cases *	OR (95% CI) †	OR (95% CI) ‡
**Smoking**			
Yes	1886	Reference	Reference
No	1751	0.81 (0.74-0.87)	0.80 (0.74-0.88)
**Alcohol consumption**			
Heavy drinking	312	Reference	Reference
No/mild drinking	3325	0.72 (0.62-0.83)	0.83 (0.71-0.97)
**Active physical exercise**			
No	1977	Reference	Reference
Yes	1660	0.74 (0.69-0.80)	0.85 (0.78-0.92)
**BMI**			
≥25 (Overweight)	2109	Reference	Reference
<25 (Non-overweight)	1528	0.50 (0.46-0.54)	0.59 (0.54-0.64)
**Lifestyle index (scored 0-4)**			
Unfavorable (0-1)	816	Reference	Reference
Intermediate (2-3)	2405	0.57 (0.51-0.63)	0.62 (0.55-0.69)
Favorable (4)	416	0.34 (0.30-0.40)	0.40 (0.35-0.47)
P for trend		<0.001	<0.001

* 1748 cases before Screening Across the Lifespan Twin study survey were excluded.

† Adjusted for age, sex, and education.

‡ Adjusted for age, sex, education, marital status, hypertension, and birth weight, as well as body mass index, smoking, alcohol consumption, and active physical exercise, if applicable.

For peer review only

For peer review only - http://bmjopen.bmj.com/site/about/guidelines.xhtml

Figure 1. Joint effect of low birth weight (LBW) and lifestyle (smoking status, alcohol consumption, active physical exercise, and body mass index) on cardiometabolic diseases (CMDs).

Multi-adjusted odds ratios (95% confidence interval) of CMDs in relation to joint exposure of LBW and lifestyle from Generalized Estimating Equation models (adjusted for age, sex, education, marital status, and hypertension).

* *P*-value<0.001 refers to the difference in the risk of CMDs between participants with LBW who have a favorable lifestyle vs. those with LBW who have an unfavorable lifestyle.

For peer review only

For peer review only - http://bmjopen.bmj.com/site/about/guidelines.xhtml

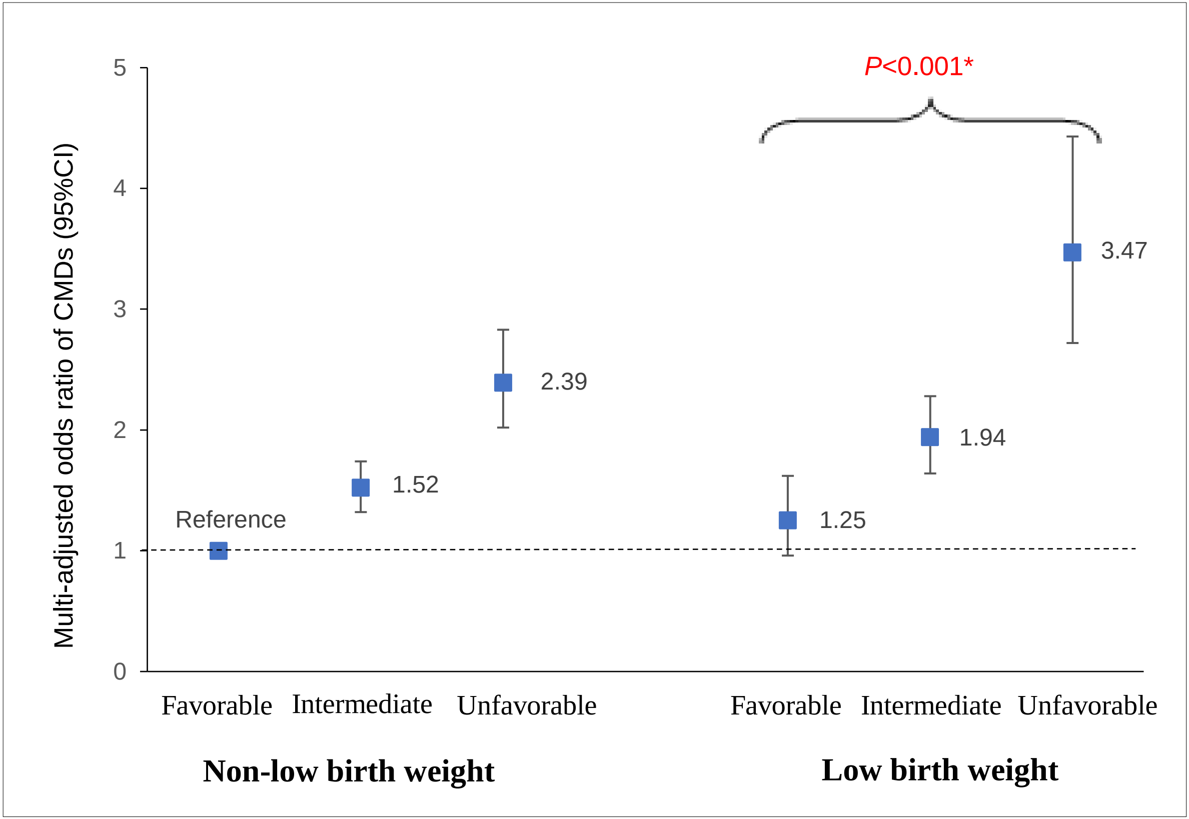
P<0.001*
Multi-adjusted odds ratio of CMDs (95%CI)
5
4
3
2
1
0
Reference
1.52
2.39
1.25
1.94
3.47
Favorable
Intermediate
Unfavorable
Favorable
Intermediate
Unfavorable
Non-low birth weight
Low birth weight

Supplemental Materials

Including: Tables-10; Figure-1

Table S1. International Classification of Disease (ICD) code of cardiometabolic diseases

Table S2. The relationship between low birth weight and numbers of cardiometabolic diseases (CMDs): results from Generalized Estimating Equation

Table S3. The dose-dependent relationship between low birth weight and cardiometabolic disease: results from Generalized Estimating Equation

Table S4. Odds ratios (ORs) and 95% confidence intervals (CIs) of cardiometabolic diseases in relation to the joint exposure of lifestyle (smoking status, alcohol consumption, active physical exercise, and body mass index) and low birth weight (LBW) from Generalized Estimation Equation models

Table S5. Additive interaction between lifestyle (smoking status, alcohol consumption, active physical exercise, and body mass index) and low birth weight (LBW) for the risk of cardiometabolic diseases

Table S6. Odds ratios (ORs) and 95% confidence intervals (CIs) of birth weight in relation to CMDs by sex: results from Generalized Estimating Equation

Table S7. Odds ratios (ORs) and 95% confidence intervals (CIs) of birth weight in relation to CMDs in adulthood further adjusted for survival status: results from Generalized Estimating Equation models

Table S8. Odds ratios (ORs) and 95% confidence intervals (CIs) of birth weight in relation to cardiometabolic diseases in adulthood by excluding cardiometabolic diseases onset before screening: results from Generalized Estimating Equation (n=18301)

Table S9. Odds ratios (ORs) and 95% confidence intervals (CIs) of birth weight in relation to cardiometabolic diseases in adulthood by excluding data with missing values for covariate: results from Generalized Estimating Equation (n=18349)

Table S10. Odds ratios (ORs) and 95% confidence intervals (CIs) of low birth weight (LBW) in relation to cardiometabolic diseases in adulthood stratified by consistency of birth weight: results from Generalized Estimating Equation

Figure S1. Flow chart of the study population

For peer review only

For peer review only - http://bmjopen.bmj.com/site/about/guidelines.xhtml

Table S1. International Classification of Disease (ICD) code of cardiometabolic diseases

Cardiometabolic diseases	ICD-7	ICD-8	ICD-9	ICD-10
Coronary heart disease	420	410-414	410-414	I20-I25
Heart failure	434	427	428	I50
Ischemic stroke	332-334	432-438	433-437	I63-I68, G47
Hemorrhagic stroke	330-331	430-431	430-432	I60-I62
Type 2 diabetes mellitus	260	250	250	E11-E14

For peer review only

For peer review only - http://bmjopen.bmj.com/site/about/guidelines.xhtml

Table S2. The relationship between low birth weight and numbers of cardiometabolic diseases (CMDs): results from Generalized Estimating Equation

CMDs status	No. of participants	Low birth weight		
		No. of cases	Basic-adjusted OR (95% CI) *	Multi-adjusted OR (95% CI) †
No	14444	2575	Reference	Reference
Any one	5335	1423	1.43 (1.31-1.55)	1.37 (1.25-1.50)
Only one	3932	989	1.32 (1.21-1.45)	1.28 (1.17-1.41)
Any two	1174	355	1.56 (1.36-1.80)	1.48 (1.28-1.72)
Any three or more	229	79	1.94 (1.47-2.56)	1.82 (1.37-2.42)
P for trend			<0.001	<0.001

* Adjusted for age, sex, and education.

† Adjusted for age, sex, education, body mass index, smoking, alcohol consumption, marital status, physical exercise, and hypertension.

For peer review only

For peer review only - http://bmjopen.bmj.com/site/about/guidelines.xhtml

Table S3. The dose-dependent relationship between low birth weight and cardiometabolic disease: results from Generalized Estimating Equation

Birth weight	No. of Case	Basic-adjusted OR (95% CI) *	Multi-adjusted OR (95% CI) †
Continuous		0.83 (0.79-0.88)	0.84 (0.80-0.89)
Categorical			
<1.7	622	1.54 (1.36-1.74)	1.45 (1.28-1.66)
1.7-2.0kg	801	1.35 (1.22-1.49)	1.32 (1.18-1.47)
≥2.0kg	3912	Reference	Reference
P for trend		<0.001	<0.001

* Adjusted for age, sex, and education.

† Adjusted for age, sex, education, body mass index, smoking, alcohol consumption, marital status, physical exercise, and hypertension.

For peer review only

For peer review only - http://bmjopen.bmj.com/site/about/guidelines.xhtml

Table S4. Odds ratios (ORs) and 95% confidence intervals (CIs) of cardiometabolic diseases in relation to the joint exposure of lifestyle (smoking status, alcohol consumption, active physical exercise, and body mass index) and low birth weight (LBW) from Generalized Estimation Equation models

Joint exposure		No. of subjects *	Cardiometabolic diseases		
Lifestyle index	LBW		Cases	Basic-adjusted OR (95% CI) †	Multi-adjusted OR (95% CI) ‡
Favorable	No	2533	314	Reference	Reference
Intermediate	No	9751	1795	1.65 (1.44-1.87)	1.52 (1.32-1.74)
Unfavorable	No	2274	620	2.90 (2.47-3.40)	2.39 (2.02-2.83)
Favorable	Yes	570	102	1.32 (1.03-1.70)	1.25 (0.96-1.62)
Intermediate	Yes	2362	610	2.18 (1.86-2.54)	1.94 (1.64-2.28)
Unfavorable	Yes	541	196	3.89 (3.08-4.90)	3.47 (2.72-4.43)

* 1748 cases before Screening Across the Lifespan Twin study survey were exclude.

† Adjusted for age, sex, education.

‡ Adjusted for age, sex, education, marital status, and hypertension.

For peer review only

For peer review only - http://bmjopen.bmj.com/site/about/guidelines.xhtml

Table S5. Additive interaction between lifestyle (smoking status, alcohol consumption, active physical exercise, and body mass index) and low birth weight (LBW) for the risk of cardiometabolic diseases

Joint exposure		No. of subjects *	Cardiometabolic diseases		
Lifestyle index	LBW		Cases	Basic-adjusted OR (95% CI) †	Multi-adjusted OR (95% CI) ‡
Favorable/Intermediate	No	12284	2109	Reference	Reference
Unfavorable	No	2274	620	1.91 (1.70-2.14)	1.68 (1.49-1.90)
Favorable/Intermediate	Yes	2932	712	1.33 (1.20-1.47)	1.28 (1.14-1.42)
Unfavorable	Yes	541	196	2.56 (2.09-3.15)	2.44 (1.97-3.03)

* 1748 cases before Screening Across the Lifespan Twin study survey were exclude.

† Adjusted for age, sex, education.

‡ Adjusted for age, sex, education, marital status, and hypertension.

Measures of additive interaction for cardiometabolic diseases:

Relative excess risk due to interaction: 0.485, 95%CI: -0.044–1.014, P=0.07;

Attributable proportion due to interaction: 0.199, 95%CI: 0.016–0.381, P=0.03;

Synergy index: 1.506, 95% CI: 1.001–2.267, P<0.001.

For peer review only - http://bmjopen.bmj.com/site/about/guidelines.xhtml

Table S6. Odds ratios (ORs) and 95% confidence intervals (CIs) of birth weight in relation to CMDs by sex: results from Generalized Estimating Equation

Birth weight (kg)	No. of Cases	OR (95% CI) *	OR (95% CI) †
Male			
<2.0	564	1.39 (1.20-1.61)	1.44 (1.23-1.69)
2.0-3.0	1050	Reference	Reference
>3.0	642	1.06 (0.93-1.21)	1.07 (0.93-1.23)
Female			
<2.0	859	1.47 (1.32-1.63)	1.36 (1.21-1.52)
2.0-3.0	1747	Reference	Reference
>3.0	473	1.06 (0.94-1.19)	1.04 (0.91-1.19)

* Adjusted for age, sex, and education.

† Adjusted for age, sex, education, body mass index, smoking, alcohol consumption, marital status, physical exercise, and hypertension.

For peer review only

Table S7. Odds ratios (ORs) and 95% confidence intervals (CIs) of birth weight in relation to CMDs in adulthood further adjusted for survival status: results from Generalized Estimating Equation models

Birth weight (kg)	No. of Cases	OR (95% CI) *
<2.0	1423	1.38 (1.26-1.52)
2.0-3.0	2797	Reference
>3.0	1115	1.05 (0.95-1.16)

* Adjusted for age, sex, education, body mass index, smoking, alcohol consumption, marital status, physical exercise, hypertension, and death.

For peer review only

For peer review only - http://bmjopen.bmj.com/site/about/guidelines.xhtml

Table S8. Odds ratios (ORs) and 95% confidence intervals (CIs) of birth weight in relation to cardiometabolic diseases in adulthood by excluding cardiometabolic diseases onset before screening: results from Generalized Estimating Equation (n=18301)

Birth weight (kg)	No. of Cases	OR (95% CI) *	OR (95% CI) †
<2.0	908	1.34 (1.22-1.48)	1.30 (1.17-1.45)
2.0-3.0	1969	Reference	Reference
>3.0	760	1.03 (0.93-1.13)	1.02 (0.92-1.14)

* Adjusted for age, sex, and education.

† Adjusted for age, sex, education, body mass index, smoking, alcohol consumption, marital status, physical exercise, and hypertension.

For peer review only

Table S9. Odds ratios (ORs) and 95% confidence intervals (CIs) of birth weight in relation to cardiometabolic diseases in adulthood by excluding data with missing values for covariate: results from Generalized Estimating Equation (n=18349)

Birth weight (kg)	No. of Cases	OR (95% CI) *	OR (95% CI) †
<2.0	1184	1.49 (1.36-1.63)	1.43 (1.30-1.58)
2.0-3.0	2359	Reference	Reference
>3.0	937	1.05 (0.96-1.15)	1.03 (0.93-1.14)

* Adjusted for age, sex, and education.

† Adjusted for age, sex, education, body mass index, smoking, alcohol consumption, marital status, physical exercise, and hypertension.

For peer review only

For peer review only - http://bmjopen.bmj.com/site/about/guidelines.xhtml

Table S10. Odds ratios (ORs) and 95% confidence intervals (CIs) of low birth weight (LBW) in relation to cardiometabolic diseases in adulthood stratified by consistency of birth weight: results from Generalized Estimating Equation

Birth weight (kg)	No. of Cases	OR (95% CI) *	OR (95% CI) †
Concordance			
LBW	347	1.53 (1.29-1.82)	1.47 (1.23-1.76)
Non-LBW	1370	Reference	Reference
Discordance			
LBW	334	1.16 (0.97-1.39)	1.13 (0.93-1.39)
Non-LBW	310	Reference	Reference

* Adjusted for age, sex, and education.

† Adjusted for age, sex, education, body mass index, smoking, alcohol consumption, marital status, physical exercise, and hypertension.

For peer review only - http://bmjopen.bmj.com/site/about/guidelines.xhtml

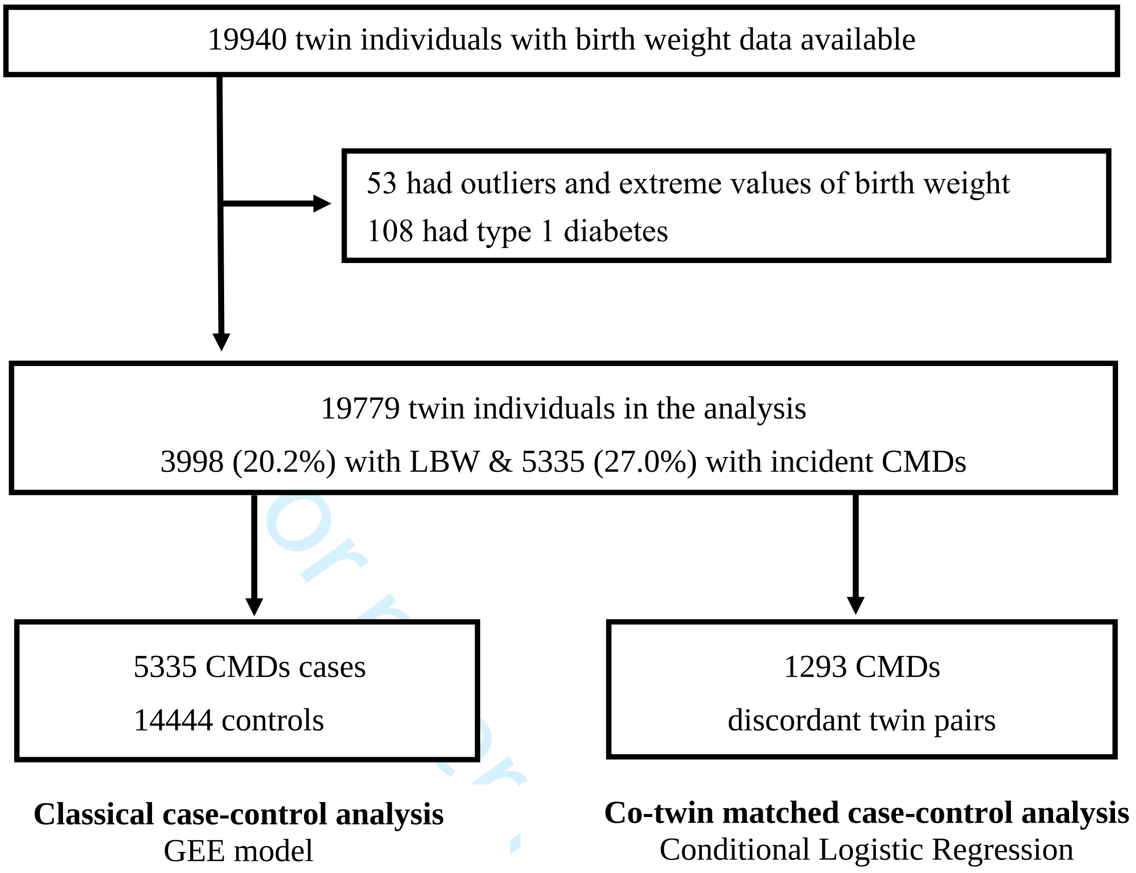

Figure S1. Flow chart of the study population

Abbreviations: LBW, low birth weight; CMDs, cardiometabolic diseases; GEE, generalized estimating equation.

For peer review only - http://bmjopen.bmj.com/site/about/guidelines.xhtml

STROBE Statement—Checklist of items that should be included in reports of ***case-control studies***

	Item No	Recommendation	Page No
**Title and abstract**	1	(*a*) Indicate the study's design with a commonly used term in the title or the abstract	1, 2
		(*b*) Provide in the abstract an informative and balanced summary of what was done and what was found	2-3
**Introduction**			
Background/rationale	2	Explain the scientific background and rationale for the investigation being reported	4-5
Objectives	3	State specific objectives, including any prespecified hypotheses	5
**Methods**			
Study design	4	Present key elements of study design early in the paper	5
Setting	5	Describe the setting, locations, and relevant dates, including periods of recruitment, exposure, follow-up, and data collection	5-8
Participants	6	(*a*) Give the eligibility criteria, and the sources and methods of case ascertainment and control selection. Give the rationale for the choice of cases and controls	6-7
		(*b*) For matched studies, give matching criteria and the number of controls per case	8-9
Variables	7	Clearly define all outcomes, exposures, predictors, potential confounders, and effect modifiers. Give diagnostic criteria, if applicable	5-9
Data sources/ measurement	8*	For each variable of interest, give sources of data and details of methods of assessment (measurement). Describe comparability of assessment methods if there is more than one group	5-8
Bias	9	Describe any efforts to address potential sources of bias	8-9
Study size	10	Explain how the study size was arrived at	5
Quantitative variables	11	Explain how quantitative variables were handled in the analyses. If applicable, describe which groupings were chosen and why	6
Statistical methods	12	(*a*) Describe all statistical methods, including those used to control for confounding	8-9
		(*b*) Describe any methods used to examine subgroups and interactions	8-9
		(*c*) Explain how missing data were addressed	8
		(*d*) If applicable, explain how matching of cases and controls was addressed	8
		(*e*) Describe any sensitivity analyses	12
**Results**			
Participants	13*	(a) Report numbers of individuals at each stage of study—eg numbers potentially eligible, examined for eligibility, confirmed eligible, included in the study, completing follow-up, and analysed	10
		(b) Give reasons for non-participation at each stage	-
		(c) Consider use of a flow diagram	Supplemental Figure S1
Descriptive data	14*	(a) Give characteristics of study participants (eg demographic, clinical, social) and information on exposures and potential confounders	10
		(b) Indicate number of participants with missing data for each variable	-

For peer review only - http://bmjopen.bmj.com/site/about/guidelines.xhtml

		of interest	
Outcome data	15*	Report numbers in each exposure category, or summary measures of exposure	10
Main results	16	(*a*) Give unadjusted estimates and, if applicable, confounder-adjusted estimates and their precision (eg, 95% confidence interval). Make clear which confounders were adjusted for and why they were included	10-12
		(*b*) Report category boundaries when continuous variables were categorized	10
		(*c*) If relevant, consider translating estimates of relative risk into absolute risk for a meaningful time period	-
Other analyses	17	Report other analyses done—eg analyses of subgroups and interactions, and sensitivity analyses	12
**Discussion**			
Key results	18	Summarise key results with reference to study objectives	12
Limitations	19	Discuss limitations of the study, taking into account sources of potential bias or imprecision. Discuss both direction and magnitude of any potential bias	14-15
Interpretation	20	Give a cautious overall interpretation of results considering objectives, limitations, multiplicity of analyses, results from similar studies, and other relevant evidence	12-15
Generalisability	21	Discuss the generalisability (external validity) of the study results	15
**Other information**			
Funding	22	Give the source of funding and the role of the funders for the present study and, if applicable, for the original study on which the present article is based	17

*Give information separately for cases and controls.

Note: An Explanation and Elaboration article discusses each checklist item and gives methodological background and published examples of transparent reporting. The STROBE checklist is best used in conjunction with this article (freely available on the Web sites of PLoS Medicine at http://www.plosmedicine.org/, Annals of Internal Medicine at http://www.annals.org/, and Epidemiology at http://www.epidem.com/). Information on the STROBE Initiative is available at http://www.strobe-statement.org.

For peer review only

Association of low birth weight with cardiometabolic diseases in Swedish twins: A population-based cohort study

Journal:	*BMJ Open*
Manuscript ID	bmjopen-2020-048030.R2
Article Type:	Original research
Date Submitted by the Author:	12-May-2021
Complete List of Authors:	Li, Xuerui; Tianjin Medical University, Department of Epidemiology and Biostatistics, School of Public Health<br>Yang, Rongrong; Tianjin University of Traditional Chinese Medicine, Public Health Science and Engineering College<br>Yang, Wenzhe; Tianjin Medical University, Department of Epidemiology and Biostatistics, School of Public Health<br>Xu, Hui; Beijing Children's Hospital Capital Medical University, Big Data and Engineering Research Center<br>Song, Ruixue; Tianjin Medical University, Department of Epidemiology and Biostatistics, School of Public Health<br>Qi, Xiuying; Tianjin Medical University, Department of Epidemiology and Biostatistics, School of Public Health<br>Xu, Weili; Karolinska Institutet, Aging Research Center, Department of Neurobiology, Health Care Sciences and Society; Tianjin Medical University, Department of Epidemiology and Biostatistics, School of Public Health
**Primary Subject Heading**:	Epidemiology
Secondary Subject Heading:	Cardiovascular medicine, Diabetes and endocrinology, Public health
Keywords:	EPIDEMIOLOGY, PUBLIC HEALTH, Adult cardiology < CARDIOLOGY, General diabetes < DIABETES & ENDOCRINOLOGY

SCHOLARONE™
Manuscripts

I, the Submitting Author has the right to grant and does grant on behalf of all authors of the Work (as defined in the below author licence), an exclusive licence and/or a non-exclusive licence for contributions from authors who are: i) UK Crown employees; ii) where BMJ has agreed a CC-BY licence shall apply, and/or iii) in accordance with the terms applicable for US Federal Government officers or employees acting as part of their official duties; on a worldwide, perpetual, irrevocable, royalty-free basis to BMJ Publishing Group Ltd ("BMJ") its licensees and where the relevant Journal is co-owned by BMJ to the co-owners of the Journal, to publish the Work in this journal and any other BMJ products and to exploit all rights, as set out in our licence.

The Submitting Author accepts and understands that any supply made under these terms is made by BMJ to the Submitting Author unless you are acting as an employee on behalf of your employer or a postgraduate student of an affiliated institution which is paying any applicable article publishing charge ("APC") for Open Access articles. Where the Submitting Author wishes to make the Work available on an Open Access basis (and intends to pay the relevant APC), the terms of reuse of such Open Access shall be governed by a Creative Commons licence – details of these licences and which Creative Commons licence will apply to this Work are set out in our licence referred to above.

Other than as permitted in any relevant BMJ Author's Self Archiving Policies, I confirm this Work has not been accepted for publication elsewhere, is not being considered for publication elsewhere and does not duplicate material already published. I confirm all authors consent to publication of this Work and authorise the granting of this licence.

For peer review only

Association of low birth weight with cardiometabolic diseases in Swedish twins: A population-based cohort study

Xuerui Li[1,2,3], MSc; Rongrong Yang[4], PhD; Wenzhe Yang[1,2,3], MPH; Hui Xu[5], PhD; Ruixue Song[1,2,3], MSc; Xiuying Qi[1,2,3]*, PhD; Weili Xu[1,2,3,6]*, PhD

Affiliations of authors:

[1] Department of Epidemiology and Biostatistics, School of Public Health, Tianjin Medical University, Tianjin, China

[2] Tianjin Key Laboratory of Environment, Nutrition and Public Health, Tianjin, China

[3] Center for International Collaborative Research on Environment, Nutrition and Public Health, Tianjin, China

[4] Public Health Science and Engineering College, Tianjin University of Traditional Chinese Medicine, Tianjin, China

[5] Big Data and Engineering Research Center, Beijing Children's Hospital, Capital Medical University, National Center for Children's Health, Beijing, China

[6] Aging Research Center, Department of Neurobiology, Health Care Sciences and Society, Karolinska Institutet and Stockholm University, Stockholm, Sweden

***Corresponding authors**

Weili Xu & Xiuying Qi, Professors
Dept. of Epidemiology & Biostatistics, School of Public Health
Tianjin Medical University, Tianjin, China
Qixiangtai Road 22, Heping District, 300070, Tianjin, PR, China
Fax number: +86 022-83336727; Phone: +86 022-83336727
Email: xuweili@tmu.edu.cn; qixiuying@tmu.edu.cn

Weili Xu, Associate Professor & Senior Researcher
Aging Research Center, Karolinska Institutet
Tomtebodavägen 18A Floor 10, SE-171 65 Solna, Stockholm, Sweden
Phone: +46 8 524 858 26; Email: weili.xu@ki.se

Word count: Title-97 (no space); Abstract-292; Text only-3583; References-51; Tables-4; Figure-1; Supplementary Tables-10; Supplementary Figure-1.

For peer review only
For peer review only - http://bmjopen.bmj.com/site/about/guidelines.xhtml

Abstract

Objective: To examine the association between low birth weight (LBW) and cardiometabolic diseases (CMDs, including heart disease, stroke, and type 2 diabetes mellitus) in adulthood, and to explore whether genetic, early-life environmental, and healthy lifestyle factors play a role in this association.

Design: A population-based twin study.

Setting: Twins from the Swedish Twin Registry who were born in 1958 or earlier participated in the Screening Across the Lifespan Twin (SALT) study for a full-scale screening during 1998-2002 and were followed until 2014.

Participants: 19,779 twin individuals in Sweden with birth weight data available (mean age: 55.45 years).

Primary and secondary outcome measures: CMDs were assessed based on self-reported medical records, medication use, and records from the National Patient Registry. A lifestyle index encompassing smoking status, alcohol consumption, exercise levels, and body mass index was derived from the SALT survey and categorized as unfavorable, intermediate, or favorable. Data were analyzed using generalized estimating equation (GEE) models and conditional logistic regression models.

Results: Of all participants, 3998 (20.2%) had LBW and 5335 (27.0%) had incident CMDs (mean age at onset: 63.64±13.26 years). In GEE models, the odds ratio (OR, 95% confidence interval [CI]) of any CMD was 1.39 (1.27-1.52) for LBW. In conditional logistic regression models, the LBW-CMDs association became non-significant (OR [95% CI] = 1.21 [0.94-1.56]). The difference in ORs from the two models was statistically significant (P<0.001). In

For peer review only - http://bmjopen.bmj.com/site/about/guidelines.xhtml

the joint effect analysis, the multi-adjusted OR (95% CI) of CMDs was 3.47 (2.72-4.43) for participants with LBW plus an unfavorable lifestyle and 1.25 (0.96-1.62) for those with LBW plus a favorable Lifestyle.

Conclusions: LBW is associated with an increased risk of adult CMDs, and genetic and early-life environmental factors may account for this association. However, a favorable lifestyle profile may modify this risk.

Key words: Population-based twin study; Birth weight; Cardiometabolic disease; Swedish twins; Lifestyle

Strengths and limitations of this study:

- This study provides an extraordinary opportunity to explore the association between low birth weight and cardiometabolic diseases by using a twin study design to control for some unmeasured confounders.
- The investigation into factors that might compensate for the risk effect of low birth weight on cardiometabolic diseases is unique.
- Birth weight was based on self-reports and non-differential misclassification among different birth weight groups could not be ruled out, possibly leading to an underestimation of the observed associations.
- Some prenatal factors (such as gestational age, maternal smoking during pregnancy, or premature birth) could not be controlled for, as information on these factors was not available.
- Potential variations of lifestyle factors during the follow-up also could not be assessed.

For peer review only - http://bmjopen.bmj.com/site/about/guidelines.xhtml
For peer review only

Introduction

With population aging has come an increase in the prevalence of chronic diseases, especially heart diseases (i.e. coronary heart diseases and heart failure), stroke, and type 2 diabetes mellitus (T2DM).[1] According to the World Health Organization (WHO), heart diseases and stroke, so called cardiovascular disease (CVD), is the leading cause of disease burden and death worldwide.[2,3] About 17.6 million deaths were attributed to CVD globally in 2016.[2] Meanwhile, there were 451 million adults living with diabetes worldwide in 2017 (90% of whom had T2DM), and this number is projected to increase to 693 million by 2045.[4,5] All of these co-occurring chronic diseases have been defined as cardiometabolic diseases (CMDs).[6,7]

Recently, beyond the effects of some traditional risk factors including age, smoking, drinking, and body mass index (BMI) on individual CMDs, the role of early-life experiences in the future development of chronic diseases have drawn special attention.[8] Birth weight, an early life indicator,[9] is frequently used to explore the effects of early-life experiences on the risk of individual CMDs in adulthood. Several cohort studies have shown that low birth weight (LBW) is associated with an increased risk of coronary heart disease,[10] stroke,[11] and T2DM,[12,13] but with some inconsistent findings.[14,15] Moreover, many studies have examined the relationship between birth weight and metabolic syndrome with inconsistent results,[16-18] but no studies have investigated the association of LBW with the risk of CMDs.

CMDs are complex genetic and lifestyle-related disorders,[19-21] and birth weight may also be affected by genetic factors and intrauterine environment.[22] However, the role of the genetic and early-life environmental factors (another term for shared environmental factors), such as intrauterine environment and prenatal nutritional status, in the association between birth weight and CMDs remains unclear. Twin studies make it possible to minimize potential confounding effects of unmeasured genetic predisposition and shared early-life environment when comparisons are made between twins.[23,24] Apart from genetic factors, some modifiable

For peer review only - http://bmjopen.bmj.com/site/about/guidelines.xhtml

lifestyle factors such as not smoking, moderate alcohol consumption, engagement in physical activities, and maintaining a healthy weight have been reported to be linked to a lower risk of CVD or T2DM.[25,26] However, previous population-based cohort studies have only shown that healthy lifestyle (such as active physical activity, not smoking, moderate alcohol consumption, and BMI <25) may reduce the risk effect of LBW on the development of diabetes.[27,28] Questions remain regarding whether and to what extent healthy lifestyle may mitigate the risk of LBW on CMDs more widely.

In the present study, we aimed to 1) verify the relationship between LBW and risk of CMDs using population-based Sweden twin data and 2) explore whether genetic, early-life environmental, and healthy lifestyle factors play a role in this association.

Methods

Study population

This prospective, nested case-control study included twins from the nationwide Swedish Twin Registry (STR), which started in the 1960s.[29] From 1998 to 2002, all living twins born in 1958 or earlier were recruited to participate in the Screening Across the Lifespan Twin (SALT) study, a full-scale screening through a computer-assisted telephone interview. Of the 19,940 twin individuals in the SALT study with birth weight data available, we excluded 53 individuals with birth weights that were outliers (extreme values; i.e., birth weight ≤300 g or ≥4520 g) to minimize possible misclassification and 108 individuals with type 1 diabetes. Finally, 19,779 individuals were included in the current study (Supplemental Figure S1).

Data collection

Data on age, sex, educational attainment, marital status, and zygosity status were collected through the SALT survey.[29] Zygosity status was categorized as monozygotic, dizygotic, or undetermined zygosity on the basis of self-reported information about childhood resemblance,

For peer review only - http://bmjopen.bmj.com/site/about/guidelines.xhtml

which was validated against biological markers with 95–99% accuracy.[29] Education was dichotomized into <8 vs. ≥8 years according to the number of years of formal schooling attained. Marital status was classified into married/cohabitating vs. single (including divorced or widows/widowers).

Information on medical conditions including heart disease, stroke, T2DM, and hypertension was derived from the National Patient Registry (NPR), which covers all inpatient diagnoses in Sweden from the 1960s and outpatient (specialist clinic) diagnoses from 2001 until 2014.[30] Each medical record in the NPR included up to eight discharge diagnoses according to the International Classification of Disease (ICD) codes. The seventh revision (ICD-7) was used through 1968, the eighth revision (ICD-8) from 1969 to 1986, the ninth revision (ICD-9) from 1987 till 1996, and the tenth revision (ICD-10) from 1997 through the end of 2014.

Informed consent was acquired from all participants. Data collection procedures were approved by the Regional Ethics Committee at Karolinska Institutet, Stockholm, Sweden and the Institutional Review Board of the University of Southern California, USA.

Assessment of birth weight

Data on birth weight was collected based on self-reports from SALT or STR. Generally, LBW was defined as birth weight <2500g in singletons.[31] However, twins may experience a more unfavorable intrauterine environment, causing them to have a lower birth weight (on average 800g) than singletons.[32] Thus, birth weight in the present study was categorized as <2.0 kg (LBW), 2.0-3.0 kg (moderate birth weight [MBW]), or >3.0kg (high birth weight [HBW])[32] considering its distribution.

Ascertainment of CMD

In the current analysis, CMDs included heart disease (coronary heart disease and heart failure), stroke (ischemic stroke and hemorrhagic stroke), and T2DM, all of which were

For peer review only - http://bmjopen.bmj.com/site/about/guidelines.xhtml

diagnosed based on self-reported medical records, medication use, and NPR data. The detailed ICD codes for each disease were shown in the Supplemental Table S1.

CMD status was categorized as CMD-free and any CMD (i.e., presence any of heart disease, stroke, and/or T2DM). The any CMD group was further classified as only one CMD (heart disease, or stroke, or T2DM), any two CMDs (any two of the following: heart disease, stroke, and T2DM), and three or more CMDs (heart disease, stroke, and T2DM).

Assessment of lifestyle-related factors

Information on lifestyle factors (including smoking status, alcohol consumption, physical exercise, and BMI) was obtained from the SALT survey. In detail, smoking status was dichotomized as non-smoking vs. former/current smoker. Alcohol consumption was categorized as no/mild drinking vs. heavy drinking based on the survey question asking whether participants have ever drunk excessively over a period. Data on physical exercise was collected by a question on average exercise with seven response options: I) “almost never,” II) “much less than average,” III) “less than average,” IV) “average,” V) “more than average,” VI) “much more than average,” and VII) “maximum”,[33] and was dichotomized as “inactive” including the first four groups (I-IV) and “active” including the last three groups (V-VII). BMI in adulthood (mean age 55.45±9.05) was calculated as weight (kg) divided by squared height (m^2) and classified as underweight (<18.5), normal weight (18.5-24.9), overweight (25-29.9), and obesity (≥30) according to the WHO classification. Obesity was merged with overweight (hereafter overweight; that is, BMI ≥25), and underweight was merged with normal weight as non-overweight (BMI <25).

In the current study, on the basis of the data availability, the following four factors were considered as healthy lifestyle factors: 1) non-smoking; 2) no/mild alcohol consumption; 3) active physical exercise; 4) non-overweight in adulthood.[34] The four factors were combined into a lifestyle index with a score ranging from 0-4, with 1 point representing each factor.

For peer review only - http://bmjopen.bmj.com/site/about/guidelines.xhtml

Participants were categorized according to their score of lifestyle index: 1) unfavorable (0-1): participants who had no healthy lifestyle factors or only one; 2) intermediate (2-3): those who had two or three healthy lifestyle factors; 3) favorable (4): those who had all the healthy lifestyle factors.

Statistical analyses

The characteristics of participants in different groups were compared using Chi-square tests for categorical variables and one-way analysis of variance/Kruskal-Wallis H test for continuous variables. Missing values on education level (n=92), smoking status (n=77), alcohol consumption (n=117), marital status (n=2), physical exercise (n=1179), and BMI (n=290) were imputed using Rubin's rule for pooling estimates to obtain valid statistical inferences.[24]

In our study, two analytical strategies were applied. First, generalized estimating equation (GEE) models were used for unmatched case-control analysis. GEE models are conceptually equivalent to logistic regression for the analysis of classic case-control design but control for the clustering of twins within a pair. Second, conditional logistic regression models were used for cotwin matched case-control analysis using a pair of twins that was discordant for the outcome. Cotwin matched design (especially in monozygotic twins) appeared more informative since cases and controls were comparable with respect to genetic background and early-life environmental factors such as intrauterine environment, prenatal and postnatal nutritional status, and childhood socioeconomic status.[35,36] In both GEE and conditional logistic regression, the odds ratios (ORs) and 95% confidence intervals (CIs) were estimated for the association between birth weight (reference: MBW) and CMDs. Logistic regression was used to test the difference in ORs from GEE and conditional logistic regression models by examining the difference in the proportions of birth weight between unmatched controls and co-twin matched controls.[36] If an OR for the observed association becomes strengthened

For peer review only - http://bmjopen.bmj.com/site/about/guidelines.xhtml

or attenuated (or even disappears) in co-twin control analyses compared with that in the unmatched case-control analysis, and the difference in ORs from the two models is significant, then genetic and/or early-life environmental factors are likely to play a role in the association.[24,35,37] If the ORs are similar between the two models without a statistically significant difference, then the effect of genetic and/or early-life environmental factors in the association can be neglected.[23,36] We hypothesized that LBW would be a significant risk factor for CMDs in a classical case-control analysis, but that the association between LBW and CMDs would be attenuated in the cotwin-matched analysis after controlling for genetic, maternal, and environmental factors shared by twins. Logistic regression was used to test the difference in ORs from the GEE model and conditional logistic regression.

Considering information on lifestyle factors was obtained from the SALT questionnaire during 1998-2002, we excluded 1748 participants who developed CMDs before the SALT recruitment, and thus 18,031 participants remained for the joint effect analysis. The combined effect of the LBW (no vs. yes) and lifestyle index (unfavorable/intermediate/favorable) on the risk of CMDs was assessed by creating dummy variables based on the joint exposures to both factors. The presence of an additive interaction was examined by estimating relative excess risk due to interaction (RERI), the attributable proportion (AP), and the synergy index (S).

All the models were basic adjusted for age, sex, and education, and further adjusted for smoking, alcohol consumption, marital status, physical exercise, BMI, and hypertension. *P*-values less than 0.05 were considered statistically significant. All statistical analyses were performed using SAS statistical software version 9.4 (SAS institute, Cary, NC) and IBM SPSS Statistics 20.0 (IBM Corp, New York, NY).

Patient and public involvement

Patients and the public were not involved in the design, or conduct, or reporting of this study.

For peer review only - http://bmjopen.bmj.com/site/about/guidelines.xhtml

Results

Characteristics of the study population

Among all participants (n=19,779), 3998 (20.2%) had LBW. The average age at recruitment was 55.45 (±9.05) years. Compared with MBW individuals, those with LBW were more likely to be older, male, monozygotic twins, single, have lower education, have higher BMI, be physically inactive, and have hypertension. Participants who had HBW were more likely to be male, dizygotic twins, smokers, heavy drinkers, and have higher BMI (Table 1).

(Insert Table 1 here)

Association between birth weight and CMDs in unmatched case-control analysis

In the multi-adjusted GEE model, compared to participants with MBW, those with LBW had a significantly higher risk of coronary heart disease, heart failure, ischemic stroke, and T2DM, which were further combined as CMDs (n=5335), as shown in Table 2. LBW was associated with an increased risk of any CMD (OR 1.39, 95% CI 1.27-1.52). However, HBW was not significantly associated with any CMDs (OR 1.05, 95% CI: 0.96-1.16). Therefore, MBW and HBW were combined into non-LBW group as reference in the following analysis.

(Insert Table 2 here)

Compared to non-LBW, the OR (95% CI) for the association between LBW and any CMD was 1.37 (1.25-1.50). The multi-adjusted ORs (95% CIs) of LBW were 1.28 (1.17-1.41) for only one CMD, 1.48 (1.28-1.72) for any two CMDs, and 1.82 (1.37-2.42) for three or more CMDs (reference: CMD-free), indicating the LBW-CMDs risk became higher when multiple CMDs were co-occurring (*P* for trend <0.001) (Supplemental Table S2). Further, the OR (95% CI) of the birth weight-CMDs association was 0.84 (0.80-0.89) when birth weight was used as a continuous variable, suggesting a dose-dependent relationship between greater birth weight and lower CMDs risk (Supplemental Table S3).

Association between LBW and CMDs in co-twin matched case-control analysis

For peer review only - http://bmjopen.bmj.com/site/about/guidelines.xhtml

In the co-twin matched case-control analysis consisting of 845 dizygotic pairs and 290 monozygotic pairs, the association between LBW and any CMD was attenuated compared to the GEE model and became non-significant (OR: 1.21, 95% CI 0.94-1.56). The ORs (95% CIs) for the associations were 1.34 (0.96-1.89) in dizygotic pairs and 1.07 (0.66-1.73) in monozygotic pairs (Table 3).

The difference in ORs from the GEE model vs. conditional logistic model was statistically significant (OR 1.39, 95% CI 1.21-1.59, P<0.001), which suggested that genetic and early-life environment factors might play an important role in LBW-CMDs association.

(Insert Table 3 here)

Association between lifestyle-related factors and CMDs

In basic- and multi-adjusted GEE models, not smoking, no/moderate alcohol drinking, active physical exercise, and being non-overweight were individually related to a decreased risk of any CMD. When combined as a lifestyle index (unfavorable, intermediate, and favorable), compared to an unfavorable lifestyle profile, an intermediate and a favorable lifestyle profile were significantly associated with a lower risk of any CMD, ORs (95% CIs) were 0.62 (0.55-0.69) and 0.40 (0.35-0.47), respectively (Table 4).

(Insert Table 4 here)

Joint effect of LBW and healthy lifestyle factors on CMD risk

In the joint effect analysis, the multi-adjusted ORs (95% CIs) of any CMDs were 1.25 (0.96-1.62) for participants with LBW plus a favorable lifestyle profile, 1.94 (1.64-2.28) for those with LBW plus an intermediate lifestyle profile, and 3.47 (2.72-4.43) for those with LBW plus an unfavorable lifestyle profile (reference: those with non-LBW plus a favorable lifestyle profile) (Figure 1 and Supplemental Table S4).

The additive interaction between the unfavorable lifestyle profile and LBW on CMDs was statistically significant (AP 0.199, 95% CI 0.016-0.381, P=0.03; S 1.506, 1.001-2.267,

For peer review only

For peer review only - http://bmjopen.bmj.com/site/about/guidelines.xhtml

P<0.001), indicating that if people with LBW have a favorable or intermediate lifestyle, the risk of LBW on CMDs can be reduced by 20% (Supplemental Table S5).

(Insert Figure 1 here)

Supplementary analysis

The results were not much altered compared to those from the initial analysis when we repeated the following analyses after: 1) stratifying by sex to address possible sex differences in the CMDs[38] (Supplemental Table S6), 2) additionally adjusting for survival status considering the association between LBW and mortality[39] (Supplemental Table S7), 3) excluding participants who developed CMDs before SALT recruitment (n=1748) (Supplemental Table S8), 4) excluding participants with missing values for covariates (n=1430) (Supplemental Table S9), and 5) stratifying by twin birth weight concordance and discordance (Supplemental Table S10).

Discussion

In this large-scale, prospective, population-based nested case-control study of Swedish twins, we found that: 1) LBW was associated with an increased risk of CMDs including coronary heart disease, heart failure, ischemic stroke, and T2DM in adulthood, and the risk became higher when multiple CMDs were co-occurring; 2) Genetic background and early-life environmental factors appear to account for the LBW-CMDs association; 3) A favorable lifestyle profile may modify the risk effect of LBW on CMDs.

Over the past two decades, the relationship between birth weight and T2DM[12,13,40] has been well documented. However, reports have been inconsistent regarding the association between birth weight and coronary heart disease. Three cohort studies have reported a relationship between LBW and the risk of coronary heart disease.[10,11,41] By contrast, Banci et al. found that higher birth weight was associated with a higher risk of coronary heart disease.[14] Another

For peer review only - http://bmjopen.bmj.com/site/about/guidelines.xhtml

study showed there was no relationship between birth weight and coronary heart disease.[15] In addition, evidence on the relationship between LBW and heart failure or ischemic stroke is sparse. To our knowledge, no studies have investigated the association of LBW with the risk of CMDs. In the present study, we found that LBW was associated with about 10-40% increased risk of coronary heart disease, heart failure, ischemic stroke (not hemorrhagic stroke), and T2DM. Further, we examined the relationship between birth weight and the risk of combined CMDs and found that individuals with LBW had an almost 40% higher risk of any CMD compared to those with non-LBW.

The potential contribution of genetic susceptibility and early-life environmental factors to the LBW-CMDs association is still unclear. Previous twin cohort studies have shown that LBW is associated with an increased risk of CVD and T2DM when twins were considered as independent individuals. This association only held in outcome-discordant dizygotic twins but not in monozygotic twin pairs, suggesting that genetic mechanisms played a role in this association.[13,32,42] In the present study, we found that the LBW-CMDs association became non-significant in both dizygotic and monozygotic twin pairs by using co-twin matched analyses. These results illustrated that early-life environmental factors could play an important role in the association between LBW and subsequent CMDs, along with genetic background.

Modifiable lifestyle factors (such as smoking, drinking, physical exercise, and BMI) deserve to be studied in the context of the LBW-CMDs association. To date, only a few studies have investigated the joint effect of LBW with lifestyle factors on T2DM.[27,28,43] One of the studies included 149,794 participants from three large prospective cohorts and showed that LBW and unhealthy adulthood lifestyles encompassing smoking, non-moderate alcohol consumption, lower exercise intensity, and BMI ≥25 were jointly related to an increased risk of T2DM.[28] Another cohort study indicated that the risk of diabetes associated with LBW

For peer review only - http://bmjopen.bmj.com/site/about/guidelines.xhtml

could be eliminated in those with a high physical activity level,[27] and individuals predisposed to T2DM due to LBW could be protected from glucose intolerance by regular exercise.[43] However, no study has illustrated the joint effect of LBW and healthy lifestyle on subsequent CMDs. In the present study, we found that people with LBW and an intermediate or a favorable lifestyle profile (including not smoking, no/mild alcohol consumption, active physical exercise, and being non-overweight) had a significantly lower risk of CMDs than those who had LBW and unfavorable lifestyle profile. To our knowledge, this is the first study to provide evidence that a healthy lifestyle might compensate for the risk effect of LBW on CMDs.

Several mechanisms may explain the relationship between LBW and the risk of CMDs. Twins have a unique and highly distinctive pattern of fetal growth. Although there is a higher rate of preterm birth among twins[44] who may have lower birth weight compared to single births, a preterm fetus with LBW may have appropriate fetal growth. Actual growth restriction could occur when twins fail to adapt to an intrauterine environment. Fetal malnutrition or inappropriate growth in gestation may redirect scant energy supplies from muscle to vital tissues, causing permanent alterations in physiology, metabolism, and structure.[45,46] Nevertheless, LBW alone could not fully capture the true growth level of the fetus, and monitoring the entire period of twin pregnancy is necessary to clarify the mechanism between LBW and CMDs in twins. Additionally, some genes (such as insulin class I allele or variants of mitochondrial DNA) have been associated with both birth weight loss and insulin resistance.[47,48] All of these alterations could result in an increased risk of CVD and T2DM in adulthood. Moreover, a haplotype of the glucocorticoid receptor gene may modify the association between size at birth and glucose tolerance.[49] However, maintaining a healthy lifestyle in adulthood may mitigate the risk of CMDs by improving insulin sensitivity and body composition, as well as controlling glycemic, blood pressure, and

For peer review only - http://bmjopen.bmj.com/site/about/guidelines.xhtml

lipid profile.[50]

Strengths and Limitations

Notable strengths of our study involve the large nationwide population-based twin cohort, which provided an extraordinary opportunity to explore the association between LBW and the risk of CMDs in adulthood by controlling for some unmeasured confounders, such as genetic background and early-life environmental factors. Furthermore, our investigation of potential compensatory factors against the LBW-CMDs association is unique. Nevertheless, some limitations should be pointed out. First, hypertension was defined only based on self-reported data from the NPR, and subjects with undiagnosed hypertension might have been misclassified as hypertension-free. Thus, hypertension was not categorized as a CMD in the current study. Second, the assessment of birth weight was based on self-report so potential information bias could not be ruled out. However, such bias is more likely to be non-differential misclassification resulting in underestimation for the given associations. Third, data on gestational age and other prenatal factors (such as maternal smoking during pregnancy, premature birth, or parental socioeconomic status) were not available and could not be fully controlled for. In addition, potential variations in lifestyle factors during follow-up could not be assessed. Fourth, diet could be partially taken into account, as it is closely associated with other lifestyle factors such as smoking, alcohol consumption, physical exercise, and BMI.[51] However, data on diet was not available in the SALT study. Finally, LBW in this study was defined as <2.0 kg in twins. Caution is needed when generalizing our findings to other populations.

Conclusion

This study provides evidence that LBW is associated with increased risk of CMDs including coronary heart disease, heart failure, ischemic stroke, and T2DM. The risk of CMDs related to

For peer review only - http://bmjopen.bmj.com/site/about/guidelines.xhtml

LBW tends to increase with the number of co-occurring CMDs. Further, genetic and early-life environmental factors play an important role in the LBW-CMDs association. However, a favorable lifestyle involving not smoking, no/mild alcohol consumption, active physical exercise, and a BMI<25 may compensate for the risk effect of LBW on CMDs. Our findings highlight the need for monitoring and controlling LBW for the prevention of CMDs, and the importance of maintaining a favorable lifestyle profile in people with LBW in adulthood to reduce the risk of CMDs.

For peer review only

For peer review only - http://bmjopen.bmj.com/site/about/guidelines.xhtml

Acknowledgements

We would like to thank the Swedish Twin Registry for access to the data, and we are grateful to all the twins who took part in the study, as well as the members of the survey teams. We are grateful to Prof. Nancy L. Pedersen for her great contribution to the design of the twin data collection. The Swedish Twin Registry is managed by Karolinska Institutet and receives funding through the Swedish Research Council under the grant No. 2017-00641.

Contributors

WX conceptualized and designed the study. XL conducted the literature search, analyzed the data, and wrote the first draft. XL, RY, WY, HX, RS, XQ, and WX contributed to the discussion and interpretation of the results. WX and XQ were involved in study supervision. All authors contributed to critical revision of the manuscript for important intellectual content and gave their final approval of the version to be published. WX obtained funding for the study. XL and WX had full access to all the data in the study and take responsibility for the integrity of the data and the accuracy of the data analysis.

Funding

This work was supported by grants from the Swedish Research Council (No. 2017-00981), the National Natural Science Foundation of China (No. 81771519), the Konung Gustaf V:s och Drottning Victorias Frimurare Foundation (No. 2016-2020), Demensfonden, Strokefonden, Cornells Stiftelse and Alzheimerfonden (2018-2019). This project is part of CoSTREAM (www.costream.eu) and received funding from the European Union's Horizon 2020 research and innovation program under grant agreement No 667375. The funders had no role in the design and conduct of the study; collection, management, analysis, and interpretation of the data; preparation, review, or approval of the manuscript; and decision to submit the manuscript for publication.

Competing interests

For peer review only - http://bmjopen.bmj.com/site/about/guidelines.xhtml

There are no competing interests for any author.

Patient consent for publication

Not applicable.

Ethics approval

The approval from the Ethics Committee of the Karolinska Institutet was obtained (Dnr: 97:051)

Data availability statement

Data are available upon reasonable request.

For peer review only

For peer review only - http://bmjopen.bmj.com/site/about/guidelines.xhtml

Reference

1. Kivimäki M, Kuosma E, Ferrie JE*, et al.* Overweight, obesity, and risk of cardiometabolic multimorbidity: pooled analysis of individual-level data for 120 813 adults from 16 cohort studies from the USA and Europe. *Lancet Public Health* 2017;2:e277-85.
2. Benjamin EJ, Muntner P, Alonso A*, et al.* Heart Disease and Stroke Statistics-2019 Update: A Report From the American Heart Association. *Circulation* 2019;139:e56-528.
3. Disease GBD, Injury I, Prevalence C. Global, regional, and national incidence, prevalence, and years lived with disability for 328 diseases and injuries for 195 countries, 1990-2016: a systematic analysis for the Global Burden of Disease Study 2016. *Lancet* 2017;390:1211-59.
4. Chatterjee S, Khunti K, Davies M. Type 2 diabetes. *Lancet* 2017;389:2239-51.
5. Cho NH, Shaw JE, Karuranga S*, et al.* IDF Diabetes Atlas: Global estimates of diabetes prevalence for 2017 and projections for 2045. *Diabetes Res Clin Pract* 2018;138:271-81.
6. Wang Z, Marseglia A, Shang Y*, et al.* Leisure activity and social integration mitigate the risk of dementia related to cardiometabolic diseases: A population-based longitudinal study. *Alzheimers Dement* 2020;16:316-25.
7. Keenan T, Zhao W, Rasheed A*, et al.* Causal Assessment of Serum Urate Levels in Cardiometabolic Diseases Through a Mendelian Randomization Study. *J Am Coll Cardiol* 2016;67:407-16.
8. Smith CJ, Ryckman KK, Barnabei VM*, et al.* The impact of birth weight on cardiovascular disease risk in the Women's Health Initiative. *Nutr Metab Cardiovasc Dis* 2016;26:239-45.

For peer review only - http://bmjopen.bmj.com/site/about/guidelines.xhtml

9. Lawani SO, Demerath EW, Lopez FL*, et al.* Birth weight and the risk of atrial fibrillation in whites and African Americans: the Atherosclerosis Risk In Communities (ARIC) study. *BMC Cardiovasc Disord* 2014;14:69.

10. Rich-Edwards JW, Kleinman K, Michels KB*, et al.* Longitudinal study of birth weight and adult body mass index in predicting risk of coronary heart disease and stroke in women. *BMJ* 2005;330:1115.

11. Lawlor DA, Ronalds G, Clark H*, et al.* Birth weight is inversely associated with incident coronary heart disease and stroke among individuals born in the 1950s: findings from the Aberdeen Children of the 1950s prospective cohort study. *Circulation* 2005;112:1414-8.

12. Whincup PH, Kaye SJ, Owen CG*, et al.* Birth Weight and Risk of Type 2 Diabetes. *JAMA* 2008;300:2886.

13. Johansson S, Iliadou A, Bergvall N*, et al.* The association between low birth weight and type 2 diabetes: contribution of genetic factors. *Epidemiology* 2008;19:659-65.

14. Banci M, Saccucci P, Dofcaci A*, et al.* Birth weight and coronary artery disease. The effect of gender and diabetes. *Int J Biol Sci* 2009;5:244-8.

15. Morley R, McCalman J, Carlin JB. Birthweight and coronary heart disease in a cohort born 1857-1900 in Melbourne, Australia. *Int J Epidemiol* 2006;35:880-5.

16. Xiao X, Zhang ZX, Li WH*, et al.* Low birth weight is associated with components of the metabolic syndrome. *Metabolism* 2010;59:1282-6.

17. dos Santos Alves Pde J, AC PTH, Pinto LR*, et al.* Endothelial and metabolic disorders in adolescence: low birth weight is not an isolated risk factor. *J Pediatr Endocrinol Metab* 2015;28:407-13.

18. Briskiewicz BL, Barreto SM, do Amaral JF*, et al.* Early-life nutritional status and metabolic syndrome: gender-specific associations from a cross-sectional analysis of

For peer review only - http://bmjopen.bmj.com/site/about/guidelines.xhtml

the Brazilian Longitudinal Study of Adult Health (ELSA-Brasil). *Public Health Nutr* 2018;21:1546-53.

19. Marenberg ME, Risch N, Berkman LF*, et al.* Genetic susceptibility to death from coronary heart disease in a study of twins. *N Engl J Med* 1994;330:1041-6.

20. Almgren P, Lehtovirta M, Isomaa B*, et al.* Heritability and familiality of type 2 diabetes and related quantitative traits in the Botnia Study. *Diabetologia* 2011;54:2811-9.

21. Bak S, Gaist D, Sindrup SH*, et al.* Genetic liability in stroke: a long-term follow-up study of Danish twins. *Stroke* 2002;33:769-74.

22. Lunde A, Melve KK, Gjessing HK*, et al.* Genetic and environmental influences on birth weight, birth length, head circumference, and gestational age by use of population-based parent-offspring data. *Am J Epidemiol* 2007;165:734-41.

23. Xu W, Qiu C, Gatz M*, et al.* Mid- and late-life diabetes in relation to the risk of dementia: a population-based twin study. *Diabetes* 2009;58:71-7.

24. Yang R, Pedersen NL, Bao C*, et al.* Type 2 diabetes in midlife and risk of cerebrovascular disease in late life: a prospective nested case-control study in a nationwide Swedish twin cohort. *Diabetologia* 2019;62:1403-11.

25. Lv J, Yu C, Guo Y*, et al.* Adherence to Healthy Lifestyle and Cardiovascular Diseases in the Chinese Population. *J Am Coll Cardiol* 2017;69:1116-25.

26. Lv J, Yu C, Guo Y*, et al.* Adherence to a healthy lifestyle and the risk of type 2 diabetes in Chinese adults. *Int J Epidemiol* 2017;46:1410-20.

27. Hu CY, Mu YM, Wan Q*, et al.* Association between birth weight and diabetes: Role of body mass index and lifestyle in later life. *J Diabetes* 2020;12:10-20.

28. Li Y, Ley SH, Tobias DK*, et al.* Birth weight and later life adherence to unhealthy lifestyles in predicting type 2 diabetes: prospective cohort study. *BMJ*

For peer review only - http://bmjopen.bmj.com/site/about/guidelines.xhtml

2015;351:h3672.

29. Lichtenstein P, De Faire U, Floderus B*, et al.* The Swedish Twin Registry: a unique resource for clinical, epidemiological and genetic studies. *J Intern Med* 2002;252:184-205.

30. Patientregistret - Socialstyrelsen n.d. https://www.socialstyrelsen.se/statistik-och-data/register/alla-register/patientregistret/ Accessed Octorber 20 2020.

31. Xiao X, Zhang ZX, Cohen HJ*, et al.* Evidence of a Relationship Between Infant Birth Weight and Later Diabetes and Impaired Glucose Regulation in a Chinese Population. *Diabetes Care* 2007;31:483-7.

32. Hubinette A, Cnattingius S, Johansson ALV*, et al.* Birth weight and risk of angina pectoris: Analysis in Swedish twins. *Eur J Epidemiol* 2002;18:539-44.

33. Carlsson S, Ahlbom A, Lichtenstein P*, et al.* Shared genetic influence of BMI, physical activity and type 2 diabetes: a twin study. *Diabetologia* 2013;56:1031-5.

34. Nyberg ST, Singh-Manoux A, Pentti J*, et al.* Association of Healthy Lifestyle With Years Lived Without Major Chronic Diseases. *JAMA Intern Med* 2020;180:760-8.

35. Kato K, Sullivan PF, Evengard B*, et al.* Premorbid predictors of chronic fatigue. *Arch Gen Psychiatry* 2006;63:1267-72.

36. Xu WL, Atti AR, Gatz M*, et al.* Midlife overweight and obesity increase late-life dementia risk: a population-based twin study. *Neurology* 2011;76:1568-74.

37. Bao C, Yang R, Pedersen NL*, et al.* Overweight in midlife and risk of cancer in late life: A nationwide Swedish twin study. *Int J Cancer* 2019;144:2128-34.

38. Kuznetsova T. Sex Differences in Epidemiology of Cardiac and Vascular Disease. *Adv Exp Med Biol* 2018;1065:61-70.

39. Hoy WE, Nicol JL. The Barker hypothesis confirmed: association of low birth weight with all-cause natural deaths in young adult life in a remote Australian Aboriginal

For peer review only - http://bmjopen.bmj.com/site/about/guidelines.xhtml

community. *J Dev Orig Health Dis* 2019;10:55-62.

40. Kaijser M, Bonamy AK, Akre O*, et al.* Perinatal risk factors for diabetes in later life. *Diabetes* 2009;58:523-6.

41. Andersen LG, Angquist L, Eriksson JG*, et al.* Birth weight, childhood body mass index and risk of coronary heart disease in adults: combined historical cohort studies. *PLoS One* 2010;5:e14126.

42. Oberg S, Cnattingius S, Sandin S*, et al.* Birth weight predicts risk of cardiovascular disease within dizygotic but not monozygotic twin pairs: a large population-based co-twin-control study. *Circulation* 2011;123:2792-8.

43. Eriksson JG, Yliharsila H, Forsen T*, et al.* Exercise protects against glucose intolerance in individuals with a small body size at birth. *Prev Med* 2004;39:164-7.

44. Elliott JP, Istwan NB, Collins A*, et al.* Indicated and non-indicated preterm delivery in twin gestations: impact on neonatal outcome and cost. *J Perinatol* 2005;25:4-7.

45. Barker DJ. Fetal origins of coronary heart disease. *BMJ* 1995;311:171-4.

46. Poulsen P, Vaag A. Glucose and insulin metabolism in twins: influence of zygosity and birth weight. *Twin Res* 2001;4:350-5.

47. Dunger DB, Ong KK, Huxtable SJ*, et al.* Association of the INS VNTR with size at birth. ALSPAC Study Team. Avon Longitudinal Study of Pregnancy and Childhood. *Nat Genet* 1998;19:98-100.

48. Casteels K, Ong K, Phillips D*, et al.* Mitochondrial 16189 variant, thinness at birth, and type-2 diabetes. *Lancet* 1999;353:1499-1500.

49. Rautanen A, Eriksson JG, Kere J*, et al.* Associations of body size at birth with late-life cortisol concentrations and glucose tolerance are modified by haplotypes of the glucocorticoid receptor gene. *J Clin Endocrinol Metab* 2006;91:4544-51.

50. Marwick TH, Hordern MD, Miller T*, et al.* Exercise training for type 2 diabetes

For peer review only - http://bmjopen.bmj.com/site/about/guidelines.xhtml

mellitus: impact on cardiovascular risk: a scientific statement from the American Heart Association. *Circulation* 2009;119:3244-62.

51. Fransen HP, Boer JMA, Beulens JWJ*, et al.* Associations between lifestyle factors and an unhealthy diet. *Eur J Public Health* 2017;27:274-8.

For peer review only

For peer review only - http://bmjopen.bmj.com/site/about/guidelines.xhtml

Table 1. Characteristics of the study population (n=19779) by birth weight

Characteristics	<2.0 kg n = 3998	2.0-3.0 kg n = 11510	>3.0 kg n = 4271	*P*-value
Age (years), mean (SD)	57.37 (9.6)	55.07 (8.8)	54.70 (8.9)	<0.001
Male sex, n (%)	1307 (32.7)	3504 (30.4)	2042 (47.8)	<0.001
Education, n (%)				
<8 years	1251 (31.3)	2850 (24.8)	1009 (23.6)	<0.001
≥8 years	2747 (68.7)	8660 (75.2)	3262 (76.4)	
Marital status, n (%)				
Married/cohabited	2911 (72.8)	8749 (76.0)	3298 (77.2)	<0.001
Single	1087 (27.2)	2761 (24.0)	973 (22.8)	
Zygosity, n (%)				
Monozygosity	1027 (25.7)	2647 (23.0)	685 (16.0)	<0.001
Dizygosity	2384 (59.6)	7436 (64.6)	3021 (70.7)	
Undetermined	587 (14.7)	1427 (12.4)	565 (13.2)	
BMI, mean (SD)	25.02 (3.8)	24.67 (3.5)	25.13 (3.5)	<0.001
BMI, n (%)				
<18.5 (Underweight)	71 (1.8)	167 (1.4)	46 (1.1)	
18.5-24.9 (Normal weight)	2108 (52.7)	6600 (57.3)	2218 (52.0)	<0.001
25.0-29.9 (Overweight)	1439 (36.0)	3874 (33.7)	1623 (38.0)	
≥30 (Obese)	380 (9.5)	869 (7.6)	384 (9.0)	
Smoking status, n (%)				
Never smoked	2049 (51.2)	5825 (50.6)	1932 (45.2)	<0.001
Former/current smoker	1949 (48.8)	5685 (49.4)	2339 (54.8)	
Alcohol consumption, n (%)				
No/mild drinking	3735 (93.4)	10746 (93.4)	3884 (90.9)	<0.001
Heavy drinking	263 (6.6)	764 (6.6)	387 (9.1)	
Active physical exercise, n (%)				
No	2092 (52.3)	5736(49.8)	2101 (49.2)	0.008
Yes	1905 (48.2)	5774 (50.2)	2170 (50.8)	
Hypertension, n (%)	1299 (33.5)	2954 (25.7)	1023 (24.0)	<0.001

Data were presented as means ± standard deviations or number (%).

Abbreviations: BMI, body mass index; SD, standard deviation.

For peer review only - http://bmjopen.bmj.com/site/about/guidelines.xhtml

Table 2. Odds ratios (ORs) and 95% confidence intervals (CIs) of birth weight in relation to different subtypes of heart diseases, stroke, and diabetes in adulthood: results from Generalized Estimating Equation

Single/combined CMDs	No. of Cases	OR (95% CI) *	OR (95% CI) †
Subtypes of Heart disease			
CHD			
<2.0	622	1.33 (1.19-1.49)	1.27 (1.14-1.43)
2.0-3.0	1166	Reference	Reference
>3.0	497	1.07 (0.95-1.20)	1.08 (0.95-1.22)
HF			
<2.0	214	1.36 (1.13-1.63)	1.27 (1.05-1.53)
2.0-3.0	356	Reference	Reference
>3.0	143	1.13 (0.93-1.39)	1.12 (0.91-1.38)
Subtypes of Stroke			
IS			
<2.0	432	1.20 (1.06-1.36)	1.14 (1.01-1.30)
2.0-3.0	874	Reference	Reference
>3.0	352	1.10 (0.96-1.26)	1.12 (0.98-1.29)
HS			
<2.0	74	1.14 (0.86-1.50)	1.09 (0.82-1.44)
2.0-3.0	162	Reference	Reference
>3.0	59	0.97 (0.72-1.32)	0.99 (0.73-1.34)
T2DM			
<2.0	668	1.45 (1.30-1.61)	1.39 (1.24-1.55)
2.0-3.0	1219	Reference	Reference
>3.0	424	0.88 (0.78-0.99)	0.82 (0.72-0.93)
Any CMDs (CHD, HF, IS, T2DM)			
<2.0	1423	1.44 (1.32-1.57)	1.39 (1.27-1.52)
2.0-3.0	2797	Reference	Reference
>3.0	1115	1.06 (0.97-1.16)	1.05 (0.96-1.16)

Abbreviations: CHD, coronary heart disease; CMDs, cardiometabolic diseases; HF, heart failure; HS, hemorrhagic stroke; IS, Ischemic stroke; T2DM, type 2 diabetes mellitus.

* Adjusted for age, sex, and education.

† Adjusted for age, sex, education, body mass index, smoking, alcohol consumption, marital status, physical exercise, and hypertension.

For peer review only - http://bmjopen.bmj.com/site/about/guidelines.xhtml

Table 3. Odds ratios (ORs) and 95% confidence intervals (CIs) for the association between LBW and adult CMDs in co-twin control analysis using CMDs discordant twin pairs: results from conditional logistic regression

Co-twin control	Co-twin with CMDs					
	**All zygosity twins * (n=1293 pairs)**		**Dizygotic only (n=845 pairs)**		**Monozygotic only (n=290 pairs)**	
	Non-LBW	LBW	Non-LBW	LBW	Non-LBW	LBW
Non-LBW	804	177	549	106	162	46
LBW	153	159	90	100	45	37
Basic-adjusted OR (95% CI) †	1.20 (0.96-1.49)		1.25 (0.94-1.67)		1.03 (0.68-1.56)	
Multi-adjusted OR (95% CI) ‡	1.21 (0.94-1.56)		1.34 (0.96-1.89)		1.07 (0.66-1.73)	

Abbreviations: CMDs, cardiometabolic diseases; LBW, low birth weight.

* Contain 158 pairs of undetermined zygosity twins

† Adjusted for sex and education.

‡ Adjusted for sex, education, body mass index, smoking, alcohol consumption, marital status, physical exercise, and hypertension.

For peer review only

For peer review only - http://bmjopen.bmj.com/site/about/guidelines.xhtml

Table 4. Odds ratios (ORs) and 95% confidence intervals (CIs) of smoking, alcohol consumption, physical exercise, and body mass index (BMI) related to cardiometabolic diseases from Generalized Estimating Equation models

Lifestyle factors	No. of Cases *	OR (95% CI) †	OR (95% CI) ‡
**Smoking**			
Yes	1886	Reference	Reference
No	1751	0.81 (0.74-0.87)	0.80 (0.74-0.88)
**Alcohol consumption**			
Heavy drinking	312	Reference	Reference
No/mild drinking	3325	0.72 (0.62-0.83)	0.83 (0.71-0.97)
**Active physical exercise**			
No	1977	Reference	Reference
Yes	1660	0.74 (0.69-0.80)	0.85 (0.78-0.92)
**BMI**			
≥25 (Overweight)	2109	Reference	Reference
<25 (Non-overweight)	1528	0.50 (0.46-0.54)	0.59 (0.54-0.64)
**Lifestyle index (scored 0-4)**			
Unfavorable (0-1)	816	Reference	Reference
Intermediate (2-3)	2405	0.57 (0.51-0.63)	0.62 (0.55-0.69)
Favorable (4)	416	0.34 (0.30-0.40)	0.40 (0.35-0.47)
P for trend		<0.001	<0.001

* 1748 cases before Screening Across the Lifespan Twin study survey were excluded.

† Adjusted for age, sex, and education.

‡ Adjusted for age, sex, education, marital status, hypertension, and birth weight, as well as body mass index, smoking, alcohol consumption, and active physical exercise, if applicable.

For peer review only

Figure 1. Joint effect of low birth weight (LBW) and lifestyle (smoking status, alcohol consumption, active physical exercise, and body mass index) on cardiometabolic diseases (CMDs).

Multi-adjusted odds ratios (95% confidence interval) of CMDs in relation to joint exposure of LBW and lifestyle from Generalized Estimating Equation models (adjusted for age, sex, education, marital status, and hypertension).

[*] *P*-value<0.001 refers to the difference in the risk of CMDs between participants with LBW who have a favorable lifestyle vs. those with LBW who have an unfavorable lifestyle.

For peer review only

For peer review only - http://bmjopen.bmj.com/site/about/guidelines.xhtml

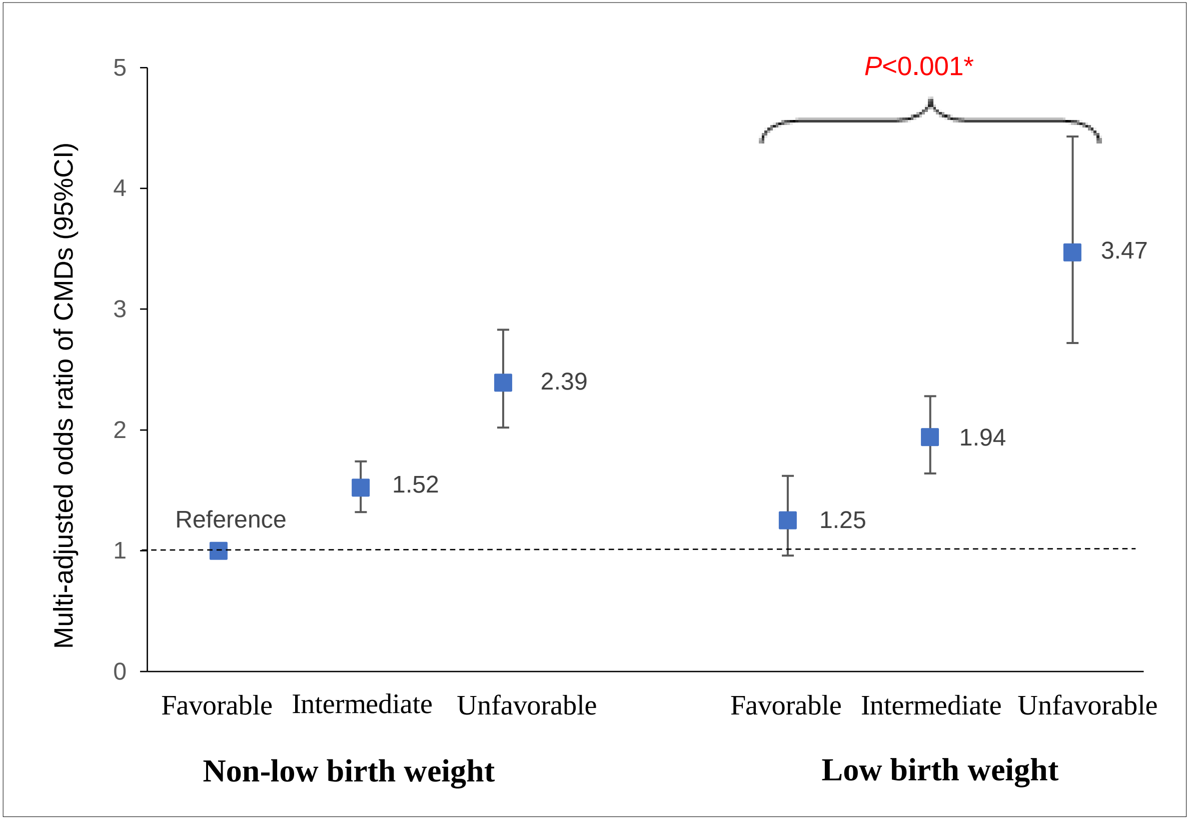
P<0.001*
Multi-adjusted odds ratio of CMDs (95%CI)
5
4
3
2
1
0
Reference
1.52
2.39
1.25
1.94
3.47
Favorable
Intermediate
Unfavorable
Favorable
Intermediate
Unfavorable
Non-low birth weight
Low birth weight

Supplemental Materials

Including: Tables-10; Figure-1

Table S1. International Classification of Disease (ICD) code of cardiometabolic diseases

Table S2. The relationship between low birth weight and numbers of cardiometabolic diseases (CMDs): results from Generalized Estimating Equation

Table S3. The dose-dependent relationship between low birth weight and cardiometabolic disease: results from Generalized Estimating Equation

Table S4. Odds ratios (ORs) and 95% confidence intervals (CIs) of cardiometabolic diseases in relation to the joint exposure of lifestyle (smoking status, alcohol consumption, active physical exercise, and body mass index) and low birth weight (LBW) from Generalized Estimation Equation models

Table S5. Additive interaction between lifestyle (smoking status, alcohol consumption, active physical exercise, and body mass index) and low birth weight (LBW) for the risk of cardiometabolic diseases

Table S6. Odds ratios (ORs) and 95% confidence intervals (CIs) of birth weight in relation to CMDs by sex: results from Generalized Estimating Equation

Table S7. Odds ratios (ORs) and 95% confidence intervals (CIs) of birth weight in relation to CMDs in adulthood further adjusted for survival status: results from Generalized Estimating Equation models

Table S8. Odds ratios (ORs) and 95% confidence intervals (CIs) of birth weight in relation to cardiometabolic diseases in adulthood by excluding cardiometabolic diseases onset before screening: results from Generalized Estimating Equation (n=18301)

Table S9. Odds ratios (ORs) and 95% confidence intervals (CIs) of birth weight in relation to cardiometabolic diseases in adulthood by excluding data with missing values for covariate: results from Generalized Estimating Equation (n=18349)

Table S10. Odds ratios (ORs) and 95% confidence intervals (CIs) of low birth weight (LBW) in relation to cardiometabolic diseases in adulthood stratified by consistency of birth weight: results from Generalized Estimating Equation

Figure S1. Flow chart of the study population

For peer review only - http://bmjopen.bmj.com/site/about/guidelines.xhtml

Table S1. International Classification of Disease (ICD) code of cardiometabolic diseases

Cardiometabolic diseases	ICD-7	ICD-8	ICD-9	ICD-10
Coronary heart disease	420	410-414	410-414	I20-I25
Heart failure	434	427	428	I50
Ischemic stroke	332-334	432-438	433-437	I63-I68, G47
Hemorrhagic stroke	330-331	430-431	430-432	I60-I62
Type 2 diabetes mellitus	260	250	250	E11-E14

For peer review only

For peer review only - http://bmjopen.bmj.com/site/about/guidelines.xhtml

Table S2. The relationship between low birth weight and numbers of cardiometabolic diseases (CMDs): results from Generalized Estimating Equation

CMDs status	No. of participants	Low birth weight		
		No. of cases	Basic-adjusted OR (95% CI) *	Multi-adjusted OR (95% CI) †
No	14444	2575	Reference	Reference
Any one	5335	1423	1.43 (1.31-1.55)	1.37 (1.25-1.50)
Only one	3932	989	1.32 (1.21-1.45)	1.28 (1.17-1.41)
Any two	1174	355	1.56 (1.36-1.80)	1.48 (1.28-1.72)
Any three or more	229	79	1.94 (1.47-2.56)	1.82 (1.37-2.42)
P for trend			<0.001	<0.001

* Adjusted for age, sex, and education.

† Adjusted for age, sex, education, body mass index, smoking, alcohol consumption, marital status, physical exercise, and hypertension.

For peer review only

For peer review only - http://bmjopen.bmj.com/site/about/guidelines.xhtml

Table S3. The dose-dependent relationship between low birth weight and cardiometabolic disease: results from Generalized Estimating Equation

Birth weight	No. of Case	Basic-adjusted OR (95% CI) *	Multi-adjusted OR (95% CI) †
Continuous		0.83 (0.79-0.88)	0.84 (0.80-0.89)
Categorical			
<1.7	622	1.54 (1.36-1.74)	1.45 (1.28-1.66)
1.7-2.0kg	801	1.35 (1.22-1.49)	1.32 (1.18-1.47)
≥2.0kg	3912	Reference	Reference
P for trend		<0.001	<0.001

* Adjusted for age, sex, and education.

† Adjusted for age, sex, education, body mass index, smoking, alcohol consumption, marital status, physical exercise, and hypertension.

For peer review only

For peer review only - http://bmjopen.bmj.com/site/about/guidelines.xhtml

Table S4. Odds ratios (ORs) and 95% confidence intervals (CIs) of cardiometabolic diseases in relation to the joint exposure of lifestyle (smoking status, alcohol consumption, active physical exercise, and body mass index) and low birth weight (LBW) from Generalized Estimation Equation models

Joint exposure		No. of subjects *	Cardiometabolic diseases		
Lifestyle index	LBW		Cases	Basic-adjusted OR (95% CI) †	Multi-adjusted OR (95% CI) ‡
Favorable	No	2533	314	Reference	Reference
Intermediate	No	9751	1795	1.65 (1.44-1.87)	1.52 (1.32-1.74)
Unfavorable	No	2274	620	2.90 (2.47-3.40)	2.39 (2.02-2.83)
Favorable	Yes	570	102	1.32 (1.03-1.70)	1.25 (0.96-1.62)
Intermediate	Yes	2362	610	2.18 (1.86-2.54)	1.94 (1.64-2.28)
Unfavorable	Yes	541	196	3.89 (3.08-4.90)	3.47 (2.72-4.43)

* 1748 cases before Screening Across the Lifespan Twin study survey were exclude.

† Adjusted for age, sex, education.

‡ Adjusted for age, sex, education, marital status, and hypertension.

For peer review only

Table S5. Additive interaction between lifestyle (smoking status, alcohol consumption, active physical exercise, and body mass index) and low birth weight (LBW) for the risk of cardiometabolic diseases

Joint exposure		No. of subjects *	Cardiometabolic diseases		
Lifestyle index	LBW		Cases	Basic-adjusted OR (95% CI) †	Multi-adjusted OR (95% CI) ‡
Favorable/Intermediate	No	12284	2109	Reference	Reference
Unfavorable	No	2274	620	1.91 (1.70-2.14)	1.68 (1.49-1.90)
Favorable/Intermediate	Yes	2932	712	1.33 (1.20-1.47)	1.28 (1.14-1.42)
Unfavorable	Yes	541	196	2.56 (2.09-3.15)	2.44 (1.97-3.03)

* 1748 cases before Screening Across the Lifespan Twin study survey were exclude.

† Adjusted for age, sex, education.

‡ Adjusted for age, sex, education, marital status, and hypertension.

Measures of additive interaction for cardiometabolic diseases:

Relative excess risk due to interaction: 0.485, 95%CI: -0.044–1.014, P=0.07;

Attributable proportion due to interaction: 0.199, 95%CI: 0.016–0.381, P=0.03;

Synergy index: 1.506, 95% CI: 1.001–2.267, P<0.001.

For peer review only

Table S6. Odds ratios (ORs) and 95% confidence intervals (CIs) of birth weight in relation to CMDs by sex: results from Generalized Estimating Equation

Birth weight (kg)	No. of Cases	OR (95% CI) *	OR (95% CI) †
Male			
<2.0	564	1.39 (1.20-1.61)	1.44 (1.23-1.69)
2.0-3.0	1050	Reference	Reference
>3.0	642	1.06 (0.93-1.21)	1.07 (0.93-1.23)
Female			
<2.0	859	1.47 (1.32-1.63)	1.36 (1.21-1.52)
2.0-3.0	1747	Reference	Reference
>3.0	473	1.06 (0.94-1.19)	1.04 (0.91-1.19)

* Adjusted for age, sex, and education.

† Adjusted for age, sex, education, body mass index, smoking, alcohol consumption, marital status, physical exercise, and hypertension.

For peer review only

Table S7. Odds ratios (ORs) and 95% confidence intervals (CIs) of birth weight in relation to CMDs in adulthood further adjusted for survival status: results from Generalized Estimating Equation models

Birth weight (kg)	No. of Cases	OR (95% CI) *
<2.0	1423	1.38 (1.26-1.52)
2.0-3.0	2797	Reference
>3.0	1115	1.05 (0.95-1.16)

* Adjusted for age, sex, education, body mass index, smoking, alcohol consumption, marital status, physical exercise, hypertension, and death.

For peer review only

For peer review only - http://bmjopen.bmj.com/site/about/guidelines.xhtml

Table S8. Odds ratios (ORs) and 95% confidence intervals (CIs) of birth weight in relation to cardiometabolic diseases in adulthood by excluding cardiometabolic diseases onset before screening: results from Generalized Estimating Equation (n=18301)

Birth weight (kg)	No. of Cases	OR (95% CI) *	OR (95% CI) †
<2.0	908	1.34 (1.22-1.48)	1.30 (1.17-1.45)
2.0-3.0	1969	Reference	Reference
>3.0	760	1.03 (0.93-1.13)	1.02 (0.92-1.14)

* Adjusted for age, sex, and education.

† Adjusted for age, sex, education, body mass index, smoking, alcohol consumption, marital status, physical exercise, and hypertension.

For peer review only

For peer review only - http://bmjopen.bmj.com/site/about/guidelines.xhtml

Table S9. Odds ratios (ORs) and 95% confidence intervals (CIs) of birth weight in relation to cardiometabolic diseases in adulthood by excluding data with missing values for covariate: results from Generalized Estimating Equation (n=18349)

Birth weight (kg)	No. of Cases	OR (95% CI) *	OR (95% CI) †
<2.0	1184	1.49 (1.36-1.63)	1.43 (1.30-1.58)
2.0-3.0	2359	Reference	Reference
>3.0	937	1.05 (0.96-1.15)	1.03 (0.93-1.14)

* Adjusted for age, sex, and education.

† Adjusted for age, sex, education, body mass index, smoking, alcohol consumption, marital status, physical exercise, and hypertension.

For peer review only

For peer review only - http://bmjopen.bmj.com/site/about/guidelines.xhtml

Table S10. Odds ratios (ORs) and 95% confidence intervals (CIs) of low birth weight (LBW) in relation to cardiometabolic diseases in adulthood stratified by consistency of birth weight: results from Generalized Estimating Equation

Birth weight (kg)	No. of Cases	OR (95% CI) *	OR (95% CI) †
Concordance			
LBW	347	1.53 (1.29-1.82)	1.47 (1.23-1.76)
Non-LBW	1370	Reference	Reference
Discordance			
LBW	334	1.16 (0.97-1.39)	1.13 (0.93-1.39)
Non-LBW	310	Reference	Reference

* Adjusted for age, sex, and education.

† Adjusted for age, sex, education, body mass index, smoking, alcohol consumption, marital status, physical exercise, and hypertension.

For peer review only

For peer review only - http://bmjopen.bmj.com/site/about/guidelines.xhtml

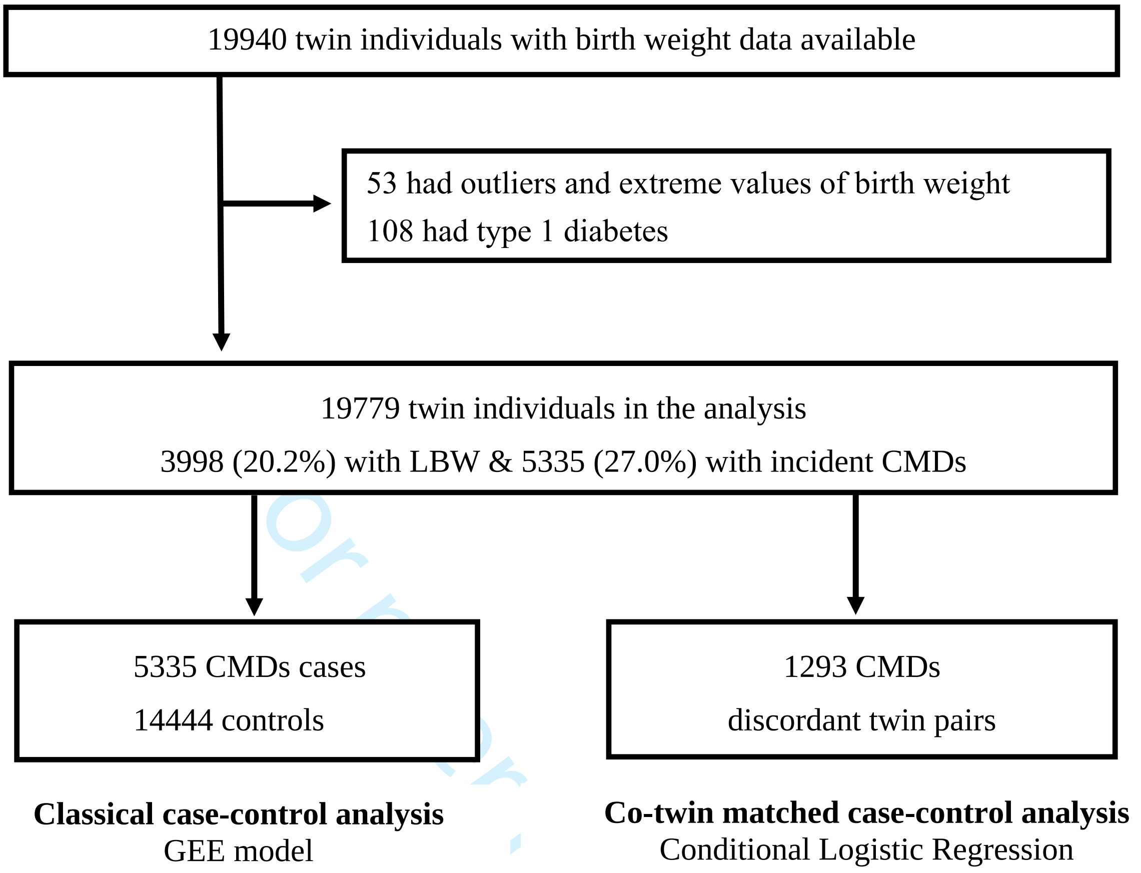

Figure S1. Flow chart of the study population

Abbreviations: LBW, low birth weight; CMDs, cardiometabolic diseases; GEE, generalized estimating equation.

For peer review only - http://bmjopen.bmj.com/site/about/guidelines.xhtml

STROBE Statement—Checklist of items that should be included in reports of ***case-control studies***

	Item No	Recommendation	Page No
**Title and abstract**	1	(*a*) Indicate the study's design with a commonly used term in the title or the abstract	1, 2
		(*b*) Provide in the abstract an informative and balanced summary of what was done and what was found	2-3
**Introduction**			
Background/rationale	2	Explain the scientific background and rationale for the investigation being reported	4-5
Objectives	3	State specific objectives, including any prespecified hypotheses	5
**Methods**			
Study design	4	Present key elements of study design early in the paper	5
Setting	5	Describe the setting, locations, and relevant dates, including periods of recruitment, exposure, follow-up, and data collection	5-8
Participants	6	(*a*) Give the eligibility criteria, and the sources and methods of case ascertainment and control selection. Give the rationale for the choice of cases and controls	6-7
		(*b*) For matched studies, give matching criteria and the number of controls per case	8-9
Variables	7	Clearly define all outcomes, exposures, predictors, potential confounders, and effect modifiers. Give diagnostic criteria, if applicable	5-9
Data sources/ measurement	8*	For each variable of interest, give sources of data and details of methods of assessment (measurement). Describe comparability of assessment methods if there is more than one group	5-8
Bias	9	Describe any efforts to address potential sources of bias	8-9
Study size	10	Explain how the study size was arrived at	5
Quantitative variables	11	Explain how quantitative variables were handled in the analyses. If applicable, describe which groupings were chosen and why	6
Statistical methods	12	(*a*) Describe all statistical methods, including those used to control for confounding	8-9
		(*b*) Describe any methods used to examine subgroups and interactions	8-9
		(*c*) Explain how missing data were addressed	8
		(*d*) If applicable, explain how matching of cases and controls was addressed	8
		(*e*) Describe any sensitivity analyses	12
**Results**			
Participants	13*	(a) Report numbers of individuals at each stage of study—eg numbers potentially eligible, examined for eligibility, confirmed eligible, included in the study, completing follow-up, and analysed	10
		(b) Give reasons for non-participation at each stage	-
		(c) Consider use of a flow diagram	Supplemental Figure S1
Descriptive data	14*	(a) Give characteristics of study participants (eg demographic, clinical, social) and information on exposures and potential confounders	10
		(b) Indicate number of participants with missing data for each variable	-

For peer review only - http://bmjopen.bmj.com/site/about/guidelines.xhtml

		of interest	
Outcome data	15*	Report numbers in each exposure category, or summary measures of exposure	10
Main results	16	(*a*) Give unadjusted estimates and, if applicable, confounder-adjusted estimates and their precision (eg, 95% confidence interval). Make clear which confounders were adjusted for and why they were included	10-12
		(*b*) Report category boundaries when continuous variables were categorized	10
		(*c*) If relevant, consider translating estimates of relative risk into absolute risk for a meaningful time period	-
Other analyses	17	Report other analyses done—eg analyses of subgroups and interactions, and sensitivity analyses	12
**Discussion**			
Key results	18	Summarise key results with reference to study objectives	12
Limitations	19	Discuss limitations of the study, taking into account sources of potential bias or imprecision. Discuss both direction and magnitude of any potential bias	14-15
Interpretation	20	Give a cautious overall interpretation of results considering objectives, limitations, multiplicity of analyses, results from similar studies, and other relevant evidence	12-15
Generalisability	21	Discuss the generalisability (external validity) of the study results	15
**Other information**			
Funding	22	Give the source of funding and the role of the funders for the present study and, if applicable, for the original study on which the present article is based	17

*Give information separately for cases and controls.

Note: An Explanation and Elaboration article discusses each checklist item and gives methodological background and published examples of transparent reporting. The STROBE checklist is best used in conjunction with this article (freely available on the Web sites of PLoS Medicine at http://www.plosmedicine.org/, Annals of Internal Medicine at http://www.annals.org/, and Epidemiology at http://www.epidem.com/). Information on the STROBE Initiative is available at http://www.strobe-statement.org.

For peer review only

For peer review only - http://bmjopen.bmj.com/site/about/guidelines.xhtml
