# Supplementary material for: Association of low birth weight with cardiometabolic diseases in Swedish twins: a population-based cohort study
Source: BMJ Open. 2021 Jun 28;11(6):e048030. doi: 10.1136/bmjopen-2020-048030 (PMC8240562; doi:10.1136/bmjopen-2020-048030)
Supplement: Reviewer comments [file bmjopen-2020-048030.reviewer_comments.pdf]

PEER REVIEW HISTORY

BMJ Open publishes all reviews undertaken for accepted manuscripts. Reviewers are asked to complete a checklist review form (**http://bmjopen.bmj.com/site/about/resources/checklist.pdf**) and are provided with free text boxes to elaborate on their assessment. These free text comments are reproduced below.

ARTICLE DETAILS

**TITLE (PROVISIONAL)**	Association of low birth weight with cardiometabolic diseases in Swedish twins: A population-based cohort study
**AUTHORS**	Li, Xuerui; Yang, Rongrong; Yang, Wenzhe; Xu, Hui; Song, Ruixue; Qi, Xiuying; Xu, Weili

VERSION 1 – REVIEW

**REVIEWER**	Martijn Finken<br>Amsterdam UMC
**REVIEW RETURNED**	29-Jan-2021

**GENERAL COMMENTS**	This study investigates associations between birth weight and cardiometabolic diseases in a population of twins.<br>I have several comments:<br>1. Abstract. In my opinion, "a prospective population-based nested case-control study" does not exist.<br>2. Abstract. Redundant information should be removed. It is not very informative that 161 individuals were excluded. Please, state only the numbers being actually analyzed.<br>3. Introduction. "So far, no studies have investigated the association of LBW with the risk of combined CMDs." In fact, previous studies have investigated associations between brith weight and metabolic syndrome in later life, and their findings were contradictory. In the paper there is no reference to these studies.<br>4. Objective. The primary objective as stated is to "examine associations between LBW and risk of CMDs in adulthood". It is not. In fact, numerous previous studies have investigated associations between birth weight and adult diseases. The second objective, i.e., "whether genetic, early-life environmental and healthy lifestyle factors play a role in this association", holds relevance.<br>5. Methods. How was the zygosity status ascertained?<br>6. Methods. I realize that there are no neonatal anthropometric reference curves for twins. However, in the absence of information on the gestational age birth weight classifications do not reflect fetal growth. Have the authors considered to perform analyses with birth weight con-/discordance (as a proxy for fetal growth)? What was the rationale behind the birth weight limits for being considered as an outlier?<br>7. Methods. Not every reader is familair with twin statistics. Please, describe in the statistical paragraph the rationale behind each step. From a statistical point of view, are "early-life environmental factors" the same as shared environmental factors? The latter term is more common in twin research.<br>8. Results. The birth weight groups differed not only in birth weight but also in a host of other characteristics. It is unclear to me how the authors controlled for these differences.<br>9. General. The standard of written English is too low. I advise the authors to seek help from a language editing service.

**REVIEWER**	Josep Figueras-Aloy<br>Universitat de Barcelona
**REVIEW RETURNED**	03-Mar-2021

**GENERAL COMMENTS**	The reviewer completed the checklist but made no further comments.

**REVIEWER**	Helen Bailey<br>Universite Paris Descartes, UMRS-1153 Equipe 7 (EPICEA), INSERM,
**REVIEW RETURNED**	11-Mar-2021

**GENERAL COMMENTS**	BMJ Open: LBW and cardiometabolic diseases Sweden<br>Thank you for the opportunity to review this interesting paper. The authors investigated the association between low birth weight (LBW) (defined as <2kg) and cardiometabolic diseases (CMD) in Sweden and the role of a healthy lifestyle. They used data from nearly 20,000 twins in the Swedish Twin Registry born on 1958 or earlier who participated in a follow-up study 1988-2002 and were followed up until 2014. They found that LBW was associated with an increased risk of adult CMD, but the risk could be modified by a healthy lifestyle.<br>Overall, the paper is well written and clear and the methods appropriate.<br>My major comment is about using birthweight as a marker for fetal growth as it is not, although birth weight in conjunction with gestation and sex is. A preterm fetus can have appropriate fetal growth but still be low birth weight. This is even more of an issue with twins who are more likely to be preterm. For example, for a female twin born at 34 weeks, 2 kgs (the indicator used for LBW) is around the 50 percentile in birthweight by gestation and sex charts, well above the 10% mark, commonly used to indicate fetal growth restriction. I used a chart based on 1990s births which may not reflect births in Sweden prior to 1960, but it might be worth finding historical percentile charts to investigate further. This issue may explain some of the inconsistency in the literature about LBW and adult chronic diseases. I suggest that the authors address this issue in the Discussion, possibly on page 13, line 56 where the Barker hypothesis about fetal undernutrition at later gestations is mentioned. I also suggest investigating whether the previous studies have included data from singletons (or not stated) where preterm rates are low or twin populations, where preterm rates are higher. Since the authors have not mentioned gestation or even preterm, I suspect that these data were not available from the STR. However, if they are, I suggest including in the analyses. If, please mention as a limitation.<br>Minor points<br>Abstract: Please write in full LBW, CMDs, T2DM the first time used. Likewise, please do not use abbreviations in the Strengths and limitation bullet points.<br>Introduction<br>Page 5; Line 10: please correct 'stroked' to stroke<br>Line 17: The citation for diabetes relates to all types of diabetes, so I suggest either using a T2DM citation or adding context about the proportion of all diabetes that is T2DM.<br>Data collection<br>Assessment of birthweight: Please clarify if the STR had birthweight data collected around the time of birth (which is more likely to be

	accurate) or not and state the proportion of the sample that these were available for. If some participants had both STR data and self-reported data, this could be used to assess the reliability of self-reported data<br>Ascertainment of CMD.<br>Rather than listing all the ICD codes for each version and for each condition, it may be easier to include these in a supplementary table.

**REVIEWER**	Rachana Singh<br>Tufts Medical Center, Newborn Medicine, Perinatal Medicine, Pediatrics
**REVIEW RETURNED**	16-Mar-2021

**GENERAL COMMENTS**	The authors have focused on an important issue of cardiometabolic outcome in adults as affected by birth weight. For this purpose they have utilized a well defined prospective dataset from the Swedish twin study. Overall the study design, methodologies and analyses are well done and do provide some new evidence on this topic of global importance. However, there are certain concerns that should be addressed b either conducting additional analyses and if these are not feasible then acknowledging them as limitations.<br>Specific Comments:<br>1. General comment to run grammar checks for typos as well as defining the abbreviations before first time use in the manuscript such as in the abstract first line LBW, CMDs, T2DM should be spelled out.<br>2. Abstract:<br>- Line 28 consider replacing "...who had outliers" with "...who were outliers"<br>- Results, please be consistent in the presentation of numerical data, the OR and 95% CI are written in different forms<br>3. Main Body of MS:<br>- Introduction: Page 5 Line 6 replace "ageing" with "aging"; Line 10 replace "stroked" with "stroke"; Line 42 replace "...CMDs is a complex genetic and lifestyle-related disorder.." with "CMDs are complex genetic and lifestyle-related disorders...".<br>- Methods:<br>Page 7- Data collection - lack of data on socioeconomic status for mother as well as the study participant is a limitation as may impact both birth weight as well as lifestyle choices. So need to mention why it was not collected/considered for analyses.<br>Page 8- Assessment of birth weight. This for me is the major limitation. Since the data on gestational age is not being included in the study for analyses the arbitrary classification of LBW < 2.0 kg as opposed to the norm <2.5kg poses an issue. Twin gestations also tend to go shorter than term gestations, so it is highly possible that the adult born < 2.0Kg may be AGA for a late preterm gestation. Similarly for an adult born at term <2.0Kg would actually be severe growth restriction and putting them in the same bracket adds bias to the analyses. The authors may consider redefining their definitions for weight assessments to universally acceptable definitions and re-run the analyses for generalizability or else notice this as a limitation. In the latter case their study would only be applicable to twin births and not the larger population in general.<br>- Statistical analyses - Done well and with rigor for the variables studied<br>- Strength and limitations: Needs to be edited based on comments above.

VERSION 1 – AUTHOR RESPONSE

Reviewer: 1
Dr. Martijn Finken, Amsterdam UMC
Comments to the Author:
This study investigates associations between birth weight and cardiometabolic diseases in a population of twins.
I have several comments:
1. Abstract. In my opinion, "a prospective population-based nested case-control study" does not exist.

We thank the reviewer for the comment. We have amended the sentence (page 2, line 6) as follows.

"**Design:** A population-based twin study."

2. Abstract. Redundant information should be removed. It is not very informative that 161 individuals were excluded. Please, state only the numbers being actually analyzed.

Following the reviewer's suggestion, we have briefly reported the participants in the abstract (page 2, lines 10-11) as follows.

"**Participants:** 19,779 twin individuals in Sweden with birth weight data available (mean age: 55.45 years)."

3. Introduction. "So far, no studies have investigated the association of LBW with the risk of combined CMDs." In fact, previous studies have investigated associations between brith weight and metabolic syndrome in later life, and their findings were contradictory. In the paper there is no reference to these studies.

We appreciate the reviewer's comment. Metabolic syndrome is usually defined as a cluster of any three or more of the following features: elevated waist circumference, elevated triglyceride level, reduced high-density lipoprotein cholesterol level, elevated blood pressure, elevated fasting glucose level, and insulin resistance (*Liao et al. Front Pediatr 2020*). Cardiometabolic diseases (CMDs) are defined as cardiovascular diseases (including heart disease and stroke) and diabetes (*Wang et al. Alzheimers Dement 2020; Wu et al. Nat Rev Cardiol 2019*). Previous studies showed that a cluster of symptoms of metabolic syndrome might be associated with various chronic diseases, including cardiovascular disease and type 2 diabetes mellitus. Thus, metabolic syndrome and CMDs are assessed differently. To our knowledge, some studies have investigated the relationship between birth weight and metabolic syndrome and shown inconsistent results (*Xiao et al. Metabolism 2010; dos Santos Alves Pde J et al. J Pediatr Endocrinol Metab 2015; Liao et al. Front Pediatr 2020*). Only a few studies have focused on the association of low birth weight with the risk of combined CMDs. In our study, we investigated the relationship between birth weight and CMDs including heart disease, stroke, and type 2 diabetes. We agree with the reviewer's comment, and we have amended the introduction and cited some of the studies on birth weight and metabolic syndrome as below (page 4, lines 16-18).

"Moreover, many studies have examined the relationship between birth weight and metabolic syndrome with inconsistent results,[16-18] but no studies have investigated the association of LBW with the risk of CMDs."

4. Objective. The primary objective as stated is to "examine associations between LBW and risk of CMDs in adulthood". It is not. In fact, numerous previous studies have investigated associations between birth weight and adult diseases. The second objective, i.e., "whether

genetic, early-life environmental and healthy lifestyle factors play a role in this association", holds relevance.

We completely understand the reviewer's comment. So far, numerous studies have addressed the associations between low birth weight and individual adult diseases, such as type 2 diabetes mellites (*Whincup et al. Jama 2008*), heart disease (*Rich-Edwards et al. BMJ 2005*), hypertension (*Law et al. Circulation 2002*), liver diseases (*Donnma et al. Medical Hypotheses 2003*), some types of cancer (*Michos et al. Int. J. Cancer 2007*), mental and neurological disorders, and other chronic diseases (*Nakano. J Atheroscler Thromb 2020*). People with abnormal birth weight may be more predisposed to metabolic abnormalities. However, there have been few studies addressing the relationship between birth weight and cardiometabolic diseases (CMDs) together (i.e., co-occurring heart disease, stroke, and type 2 diabetes mellitus), especially in the twin population. Due to the particularity of the twin population, their birth weight and the occurrence of adult diseases might be different from that of the general population. In addition, using the twin population could further explore the role of genes and early-life environmental factors between birth weight and CMDs. Thus, in the present study, our first aim was to verify the relationship mentioned using population-based Sweden twin data, and further to explore whether genetic, early-life environmental and healthy lifestyle factors play a role in this association. Based on the reviewer's comment, we have revised the words (page 5, lines 9-11) as follows.

"In the present study, we aimed to 1) verify the relationship between LBW and risk of CMDs using population-based Sweden twin data and 2) explore whether genetic, early-life environmental, and healthy lifestyle factors play a role in this association."

5. Methods. How was the zygosity status ascertained?

Zygosity information was ascertained on the basis of standard self-reported information about childhood resemblance in the Screening Across the Lifespan Twin (SALT) study questionnaire. The relevant survey question was *'During childhood, were you and your twin partner as like as "two peas in a pod" or not more alike than siblings in general?'* If both individuals of a twin pair responded *'alike as two peas in a pod',* their zygosity status was classified as monozygotic (MZ); if both responded *'not alike',* the zygosity was classified as dizygotic (DZ). If the twins did not agree, or if only one member of the pair responded to the question, the zygosity was considered 'not determined' (XZ). If the zygosity was XZ after the above question, another question concerning twin similarity would be asked further: *"How often did strangers have difficulty in distinguishing between you and your twin partner when you were children?"* If both individuals of a pair responded *'almost always or always'* or *'often'* being confused as children, their zygosity status was classified as MZ. If both responded *'seldom'* or *'almost never or never,'* zygosity status was classified as DZ. Furthermore, DNA-analysis, using 13 polymorphic DNA markers, was carried out to validate the zygosity diagnoses, and 95% to 99% of twin pairs were classified correctly (*Lichtenstein et al. J Intern Med 2002*). We have clarified the description of zygosity status (page 5, lines 24-25, and page 6, line 1) as follows.

"Zygosity status was categorized as monozygotic, dizygotic, or undetermined zygosity on the basis of self-reported information about childhood resemblance, which was validated against biological markers with 95–99% accuracy.[29]"

6. Methods. I realize that there are no neonatal anthropometric reference curves for twins. However, in the absence of information on the gestational age birth weight classifications do not reflect fetal growth. Have the authors considered to perform analyses with birth weight con-/discordance (as a proxy for fetal growth)? What was the rationale behind the birth weight limits for being considered as an outlier?

Indeed, there is no reference that defines the cutoff of birth weight for twins. Following the reviewer's suggestion, we performed a stratified analysis with birth weight con-/discordance. The results also showed the similar risk of cardiometabolic diseases related to low birth weight in the weight concordance group (OR 1.47, 95% CI: 1.23-1.76) and in the weight discordance group (OR 1.13, 95% CI: 0.93-1.39). As the reviewer commented, we have reported these results in the sensitivity analysis (page 12, lines 17-18) as follows.

"and 5) stratifying by birth weight concordance and discordance (Supplemental Table S10)."

We used a Stem-and-Leaf display to examine the extreme values (i.e., the difference between the value and the mean is more than 3 times the standard deviation) in the birthweight data of 19,940 participants. The result of Stem-and-Leaf display showed that the extremes of birth weight were ≤300 g or ≥4520 g, and these outliers were thus removed to control the information bias caused by misclassification. Furthermore, as sensitivity analysis we re-included these 53 individuals with outlier birth weights in the analysis, finding results that were similar to those from the initial analysis (OR 1.39, 95% CI: 1.26-1.52). To minimize confusion, we added an explanation in the methods (page 5, lines 18-20) as follows.

"…we excluded 53 individuals with birth weights that were outliers (extreme values; i.e., birth weight ≤300 g or ≥4520 g) to minimize possible misclassification…"

7. Methods. Not every reader is familair with twin statistics. Please, describe in the statistical paragraph the rationale behind each step. From a statistical point of view, are "early-life environmental factors" the same as shared environmental factors? The latter term is more common in twin research.

We completely understand the reviewer's concern. Twin studies provide naturally matched pairs where the confounding effects that twin pairs share in common (such as maternal status, intrauterine environment, and prenatal nutritional status) may be minimized by comparisons within twin pairs.

In our study, we used two strategies to analyze the association of low birth weight (LBW) with cardiometabolic diseases (CMDs). First, the generalized estimating equation (GEE) model was used for unmatched case-control analysis. Due to the shared genes and early-life environmental factors between a pair of twins, the twins cannot be regarded as two completely independent individuals when analyzing the twin data. The GEE model is conceptually equivalent to the logistic regression model for the analysis of classic case-control design but control for the clustering between twin pairs. Second, conditional logistic regression was used for performing the co-twin matched case-control design. In other words, we only selected pairs of twins with discordant outcomes (i.e., one twin had CMDs and the other did not), where therefore genetic background and early-life environmental factors (such as intrauterine environment and prenatal nutritional status) could be controlled for. In particular, monozygotic twins are ideal case-control pairs due to their shared genes. Finally, logistic regression was used to test the difference in odds ratios (ORs) from the GEE model and conditional logistic regression model by examining the difference in the proportion of exposure between unmatched and co-twin controls (*Kato et al. Arch Gen Psychiatry* 2006). If the observed association in the unmatched case-control analyses became strengthened or attenuated (or even disappeared) in co-twin control analyses, and the difference in ORs from the GEE and conditional logistic regression was significant, genetic and/or shared familial environmental factors were likely to play a role in the association (*Xu et al. Diabetes 2009; Xu et al. Neurology 2011; Bao et al. Int J Cancer 2018; Bao et al. Int J Cancer 2018; Yang et al. Diabetologia 2019*). In contrast, if the difference in ORs was not significant, the confounding by genetic or shared familial environmental factors in the observed association was small or null (*Bao et al. Int J Cancer 2018; Yang et al. Diabetologia 2020*).

Based on the reviewer's suggestion, we have added further description on the co-twin design in the Methods section (page 8, lines 18-25 and page 9, line 1, lines 10-12, and lines 12-16) as follows. Thanks to the reviewer for the valuable comment.

“In our study, two analytical strategies were applied. First, generalized estimating equation (GEE) models were used for unmatched case-control analysis. GEE models are conceptually equivalent to logistic regression for the analysis of classic case-control design but control for the clustering of twins within a pair. Second, conditional logistic regression models were used for cotwin matched case-control analysis using a pair of twins that was discordant for the outcome. Cotwin matched design (especially in monozygotic twins) appeared more informative since cases and controls were comparable with respect to genetic background and early-life environmental factors such as intrauterine environment, prenatal and postnatal nutritional status, and childhood socioeconomic status.”

“If the ORs are similar between the two models without a statistically significant difference, then the effect of genetic and/or early-life environmental factors in the association can be neglected.”

“We hypothesized that LBW would be a significant risk factor for CMDs in a classical case-control analysis, but that the association between LBW and CMDs would be attenuated in the cotwin-matched analysis after controlling for genetic, maternal, and environmental factors shared by twins. Logistic regression was used to test the difference in ORs from the GEE model and conditional logistic regression.”

Furthermore, in the current study, “early-life environmental factors” are the same as “shared environmental factors.’’ To avoid confusion, we have clarified this at the first mention of “early-life environmental factors” in the manuscript (page 4, line 21).

8. Results. The birth weight groups differed not only in birth weight but also in a host of other characteristics. It is unclear to me how the authors controlled for these differences.

Indeed, the distributions of age, sex, education, body mass index, smoking, alcohol consumption, marital status, physical exercise, and hypertension were different among birth weight groups as shown in Table 1. Therefore, these factors were adjusted for in multivariate logistic or GEE analysis.

9. General. The standard of written English is too low. I advise the authors to seek help from a language editing service.

Following the reviewer’s suggestion, a native English speaker has carefully corrected the grammar and language throughout the manuscript.

Reviewer: 2
Dr. Josep Figueras-Aloy, Universitat de Barcelona
Comments to the Author:
Sorry but I can't rate this job. It is difficult to evaluate such a long-term follow-up without having initial variables that are essential in establishing a prognosis: smoking during pregnancy and degree of prematurity. Ignoring and not including this data in the analysis can cause serious bias in the results. Non-existent values have also been “rounded” using Rubin’n rule, when it would have been best to suppress these cases.

We completely agree with the reviewer that smoking during pregnancy and degree of prematurity were essential factors when exploring the association between birth weight and subsequent cardiometabolic diseases (CMDs). Although these data were not available in our study, we were able to use this unique twin population to control such factors in the earliest stages of life. Twins shared the same intrauterine environment and prenatal nutritional and maternal status. Thus, twins have similar genetic and early-life environmental factors. In our study, we used two strategies to analyze the association of low birth weight (LBW) with CMDs: 1) Unmatched case-control analysis using generalized estimating equation (GEE) models, which are conceptually equivalent to logistic

regression model for the analysis of classic case-control design but control for the clustering of twins within a pair; and 2) CMDs-discordant cotwin matched case-control analysis using conditional logistic regression models. This method allows matching for unmeasured familial factors which might be related to genetic background or early-life environment. Cases and controls are comparable with respect to genetic background and early-life environmental factors including smoking during pregnancy and degree of prematurity, intrauterine environment, and prenatal nutritional and childhood socioeconomic status (*Xu et al. Diabetes 2009; Xu et al. Neurology 2011; Yang et al. Diabetologia 2019; Hubinette et al. Eur J Epidemiol 2002*). If the association found in GEE analyses becomes attenuated in cotwin matched case-control analyses, and the difference in odds ratios (ORs) from the GEE and conditional logistic regression is significant, genetic and/or shared familial environmental factors are likely to play a role in the association. In contrast, if the OR in conditional logistic regression remains similar to that from the GEE, and the difference in ORs is not significant, then the confounding by genetic or shared familial environmental factors in the observed association is small or null.

In our study, we found that LBW was associated with a higher risk of CMDs (OR: 1.39, 95% CI 1.27-1.52) in the unmatched analysis, but the association between LBW and CMDs was attenuated and became non-significant (OR: 1.21, 95% CI 0.94-1.56) in the cotwin matched analysis. The difference in ORs from the two models was statistically significant (P<0.001). Thus, our findings suggested that genetic and early-life environment factors might play an important role in the LBW-CMDs association.

We also understand the reviewer's concern about Rubin's rule for imputing the missing data. In the present study, multiple imputation by chained equation was used for missing values on education (n=92), smoking (n=77), alcohol consumption (n=117), marital status (n=2), physical exercise (n=1179), and body mass index (n=290). Additionally, in the supplementary analyses, we repeated the analysis by excluding data with missing values for these covariates, and the results were similar to those from the initial analysis.

Reviewer: 3
Dr. Helen Bailey, Universite Paris Descartes, Telethon Kids Institute
Comments to the Author:
Thank you for the opportunity to review this interesting paper. The authors investigated the association between low birth weight (LBW) (defined as <2kg) and cardiometabolic diseases (CMD) in Sweden and the role of a healthy lifestyle. They used data from nearly 20,000 twins in the Swedish Twin Registry born on 1958 or earlier who participated in a follow-up study 1988-2002 and were followed up until 2014. They found that LBW was associated with an increased risk of adult CMD, but the risk could be modified by a healthy lifestyle.

Overall, the paper is well written and clear and the methods appropriate.

We greatly appreciate the reviewer's encouraging comment.

My major comment is about using birthweight as a marker for fetal growth as it is not, although birth weight in conjunction with gestation and sex is. A preterm fetus can have appropriate fetal growth but still be low birth weight. This is even more of an issue with twins who are more likely to be preterm. For example, for a female twin born at 34 weeks, 2 kgs (the indicator used for LBW) is around the 50 percentile in birthweight by gestation and sex charts, well above the 10% mark, commonly used to indicate fetal growth restriction. I used a chart based on 1990s births which may not reflect births in Sweden prior to 1960, but it might be worth finding historical percentile charts to investigate further. This issue may explain some of the inconsistency in the literature about LBW and adult chronic diseases. I suggest that the authors address this issue in the Discussion, possibly on page 13, line 56 where the Barker

hypothesis about fetal undernutrition at later gestations is mentioned. I also suggest investigating whether the previous studies have included data from singletons (or not stated) where preterm rates are low or twin populations, where preterm rates are higher.

We understand the reviewer's comment. Although low birth weight (LBW) is mainly affected by intrauterine growth restrictions and preterm birth, only using birth weight as a marker for fetal growth may not be appropriate. Unfortunately, we were unable to find the birth weight percentile chart of Swedish twins born prior to 1960. This may be due to the lack of comprehensive statistics on birth weight at that time.

Indeed, the risk of premature birth is much higher in twins than in singletons (*Elliott et al. J Perinatol 2005; Rydhstroem et al. Twin Res 2001*). Twins may restrain the capacity of the uterus to distend and permit adequate fetal growth, thus creating risk for preterm labor. However, not all twins born prematurely with LBW had growth restrictions. Therefore, in the current study, the use of birth weight alone could not identify those twins with actual growth restrictions of the fetus. Twins with LBW but appropriate fetal growth and those with actual growth restriction will be misclassified into one group, possibly leading to underestimations of the observed associations. In this study, we intended to provide evidence for possible mechanisms underlying the association of low birth weight (defined as <2.0 kg) with increased risk of cardiometabolic diseases for twins. Following the reviewer's suggestion, we have further discussed this point in the discussion (page 14, lines 20-24) as below.

"However, the risk of preterm birth in twins is significantly higher than singletons[46]. Furthermore, a preterm fetus with LBW may also have appropriate fetal growth, especially for twins. Thus, among twins, birth weight may not reflect the actual growth restriction of the fetus. This may explain some of the contradictions in the relationship between LBW and adult chronic disease."

Since the authors have not mentioned gestation or even preterm, I suspect that these data were not available from the STR. However, if they are, I suggest including in the analyses. If, please mention as a limitation.

As the reviewer pointed out, indeed data on gestation and preterm were not available in the current study. However, in the second part of the current study, using co-twin–control design encompassed matching on parental, pregnancy, and early life factors because twins generally shared similar genetic and early-life environmental factors. Cases and controls were comparable with respect to genetic background and early-life environmental factors, such as intrauterine environment, prenatal and postnatal nutritional status, and childhood socioeconomic status (*Xu et al. Diabetes 2009; Xu et al. Neurology 2011; Yang et al. Diabetologia 2019; Hubinette et al. Eur J Epidemiol 2002*). We have also added a limitation in the discussion (page 15, lines 17-20) as follows.

"Third, data on some prenatal factors (such as gestational age, maternal smoking during pregnancy, or premature birth) and parental socioeconomic status were not available and could not be fully controlled for."

Minor points

Abstract: Please write in full LBW, CMDs, T2DM the first time used.

Likewise, please do not use abbreviations in the Strengths and limitation bullet points.

We thank the reviewer for the corrections. We have corrected all mistakes (page 2, lines 2-3 and page 3, lines 11-12 and 14-15) and have carefully checked the language throughout the manuscript.

Introduction

Page 5; Line 10: please correct 'stroked' to stroke

We thank the reviewer for the correction. We are sorry for the mistake and have corrected it (page 4, line 5).

Line 17: The citation for diabetes relates to all types of diabetes, so I suggest either using a T2DM citation or adding context about the proportion of all diabetes that is T2DM.

Following the reviewer's suggestion, we have added a sentence and reference about the proportion of T2DM in diabetes in the introduction (page 4, lines 7-8) as follows.

"Meanwhile, there were 451 million adults living with diabetes worldwide in 2017 (90% of whom had T2DM), and this number is projected to increase to 693 million by 2045.[4,5]"

Data collection

Assessment of birthweight: Please clarify if the STR had birthweight data collected around the time of birth (which is more likely to be accurate) or not and state the proportion of the sample that these were available for. If some participants had both STR data and self-reported data, this could be used to assess the reliability of self-reported data

Data on birth weight was registered in the STR based on self-reported weight at birth. Information on birth weight from birth certificates was not available in the current study. A previous study using the same twin data reported a favorable validity for self-reported birth weight by comparing self-reported birth weight data with information from participants' birth certificates (*Hubinette et al. Eur J Epidemiol 2002*).

We agree with the reviewer that self-reported birth weight could introduce information bias, which is more likely to be non-differential misclassification resulting in underestimation for the given associations. We have discussed it as a limitation in the discussion section as below (page 15, lines 15-17).

"…the assessment of birth weight was based on self-report so potential information bias could not be ruled out. However, such bias is more likely to be non-differential misclassification resulting in underestimation for the given associations."

Ascertainment of CMD.

Rather than listing all the ICD codes for each version and for each condition, it may be easier to include these in a supplementary table.

We are very grateful for the reviewer's comments. As the reviewer pointed out, we have summarized each ICD code corresponding to each specific disease in a supplementary table (Supplementary Table S1), and have described clearly in the methods section (page 6, line 23-25 and page 7, line 1).

"CMDs included heart disease (coronary heart disease and heart failure), stroke (ischemic stroke and hemorrhagic stroke), and T2DM, …… The detailed ICD codes for each disease were shown in the Supplemental Table S1."

Reviewer: 4
Dr. Rachana Singh, Tufts Medical Center
Comments to the Author:
The authors have focused on an important issue of cardiometabolic outcome in adults as affected by birth weight. For this purpose they have utilized a well defined prospective dataset from the Swedish twin study. Overall the study design, methodologies and analyses are well done and do provide some new evidence on this topic of global importance. However, there

are certain concerns that should be addressed b either conducting additional analyses and if these are not feasible then acknowledging them as limitations.

Specific Comments:
1. General comment to run grammar checks for typos as well as defining the abbreviations before first time use in the manuscript such as in the abstract first line LBW, CMDs, T2DM should be spelled out.

We appreciate the reviewer's careful review, and we have corrected the mistakes (page 2, lines 2-3). We have carefully checked the language throughout the manuscript.

2. Abstract:
- Line 28 consider replacing "...who had outliers" with "...who were outliers"

We thank the reviewer for pointing out this mistake. This phrase has been removed from the abstract based on *Reviewer 1*'s comment.

- Results, please be consistent in the presentation of numerical data, the OR and 95% CI are written in different forms

Following the reviewer's suggestion, we have presented OR and 95% CI consistently throughout the manuscript.

3. Main Body of MS:
- Introduction: Page 5 Line 6 replace "ageing" with "aging"; Line 10 replace "stroked" with "stroke"; Line 42 replace "...CMDs is a complex genetic and lifestyle-related disorder.." with "CMDs are complex genetic and lifestyle-related disorders...".

We thank the reviewer again for the corrections. We have corrected these sentences (page 4, line 2, line 5, and line 19), and carefully checked the language and grammar throughout the manuscript.

- Methods:
Page 7- Data collection - lack of data on socioeconomic status for mother as well as the study participant is a limitation as may impact both birth weight as well as lifestyle choices. So need to mention why it was not collected/considered for analyses.

Indeed, the data on socioeconomic status (such as income and occupation) for mother and participants were not available in the current study, which is based on the nationwide Swedish twin registry started in the 1960s. However, in our study, we have information on educational levels, which might be a proxy of socioeconomic status. This was adjusted as a confounding factor in the data analysis. Further, using the twin study design, early-life socioeconomic status can be controlled for in co-twin matched analysis. Twins have the same intrauterine environment and prenatal nutritional and maternal status and were generally reared together in childhood, and thus, twins share similar genetic and early-life environmental factors, including socioeconomic status for mother and participants. Our use of co-twin-control design encompassed matching on parental, pregnancy, and early life factors (*Xu et al. Diabetes 2009; Xu et al. Neurology 2011; Yang et al. Diabetologia 2019; Hubinette et al. Eur J Epidemiol 2002*), and these confounding effects might be removed by comparisons within co-twin matched pairs. As the reviewer pointed out, we have added this as a limitation in the discussion (page 15, lines 17-20) as follows.

“Third, data on some prenatal factors (such as gestational age, maternal smoking during pregnancy, or premature birth) and parental socioeconomic status were not available and could not be fully controlled for.”

Page 8- Assessment of birth weight. This for me is the major limitation. Since the data on gestational age is not being included in the study for analyses the arbitrary classification of LBW < 2.0 kg as opposed to the norm <2.5kg poses an issue. Twin gestations also tend to go shorter than term gestations, so it is highly possible that the adult born < 2.0Kg may be AGA for a late preterm gestation. Similarly for an adult born at term <2.0Kg would actually be severe growth restriction and putting them in the same bracket adds bias to the analyses. The authors may consider redefining their definitions for weight assessments to universally acceptable definitions and re-run the analyses for generalizability or else notice this as a limitation. In the latter case their study would only be applicable to twin births and not the larger population in general.

We understand the reviewer’s concern about the assessment of birth weight. Regrettably, information on gestational age was not available in this study. We used 2.0 kg as the threshold for low birth weight (LBW) for the following reasons. First, the standard definition of LBW as a birth weight <2.5 kg refers to single births, and no established definition for twins has been developed. It has been reported that twins have lower birth weight than singletons on average. Their development is thought to be similar to that of singletons until the third trimester and is on average 900 g lighter than singletons at birth (*Vågerö et al. Lancet 1994*). If twin-birth weights are referred to singleton standards, it seems that most twin individuals are identified to LBW group. 2) According to data available on birth weight in twins, the interquartile range of birth weight was 2.0 kg to 3.0 kg, and thus, we used 2.0 kg-3.0 kg as normal birth weight range, <2.0 kg as LBW, >3.0 kg as high birth weight for this study. Similarly, another article from the Swedish Twin Registry also used 2.0 kg and 3.0 kg to classify birth weight (*Hubinette et al. Eur J Epidemiol 2002*).

On the other hand, although data on gestational age were unavailable, we could control for this factor when performing the co-twin matched analysis (twins share the same intrauterine environments and gestational age). Thus, the confounding effects of gestational age could be reduced by comparisons within cotwin matched pairs. However, we acknowledge that the generalizability of our results may be limited. Our intention was to provide insight into the mechanisms behind the relationship between low birth weight and cardiometabolic diseases. Following the reviewer’s suggestion, we added this limitation to the discussion section (page 15, lines 23-25) as follows.

“Finally, LBW in this study was defined as <2.0 kg in twins. Caution is needed when generalizing our findings to other populations.”

- Statistical analyses - Done well and with rigor for the variables studied

We are grateful to the reviewer for the encouraging comment.

- Strength and limitations: Needs to be edited based on comments above.

We thank the reviewer for the careful reading. We have re-edited the strengths and limitations section of our study based on the reviewer’s comments above.

VERSION 2 – REVIEW

**REVIEWER**	Martijn Finken<br>Amsterdam UMC
**REVIEW RETURNED**	26-Apr-2021

GENERAL COMMENTS	The authors appropriately addressed my concerns. The current version of the paper is acceptable for publication.

REVIEWER	Helen Bailey<br>Universite Paris Descartes, UMRS-1153 Equipe 7 (EPICEA), INSERM,
REVIEW RETURNED	28-Apr-2021

GENERAL COMMENTS	Thank you for addressing most of my comments. However, I still have concerns about missing gestational age. As I pointed out in the last version, low birth weight (even using 2kg) tells us little about fetal growth in twins. There is a strong case not to investigate fetal origins of disease using birth weight alone so I suggest that the authors discuss the difference between inappropriate fetal growth and low gestation in twins as both have very different outcomes in the perinatal period and childhood which one would assume would continue in adulthood.

REVIEWER	Rachana Singh<br>Tufts Medical Center, Newborn Medicine, Perinatal Medicine, Pediatrics
REVIEW RETURNED	03-May-2021

GENERAL COMMENTS	I applaud the authors for detailed rebuttal to the reviewer's feedback and addressing them in the revised manuscript.

VERSION 2 – AUTHOR RESPONSE

Reviewer: 1
Dr. Martijn Finken, Amsterdam UMC
Comments to the Author:
The authors appropriately addressed my concerns. The current version of the paper is acceptable for publication.

We thank the reviewer again for the helpful comments.

Reviewer: 3
Dr. Helen Bailey, Universite Paris Descartes, Telethon Kids Institute
Comments to the Author:
Thank you for addressing most of my comments. However, I still have concerns about missing gestational age. As I pointed out in the last version, low birth weight (even using 2kg) tells us little about fetal growth in twins. There is a strong case not to investigate fetal origins of disease using birth weight alone so I suggest that the authors discuss the difference between inappropriate fetal growth and low gestation in twins as both have very different outcomes in the perinatal period and childhood which one would assume would continue in adulthood.

We understand the reviewer's concern. Regrettably, information on gestational age was not available in the current study. We are very sorry that we could not comprehensively consider the growth restriction assessed by birth weight and gestational age. However, in the second part of this twin study, we could control for gestational age when performing the co-twin matched analysis (twins share the same intrauterine environments and gestational age). Thus, the confounding effects of gestational age could be reduced by comparisons within cotwin matched pairs.

As the reviewer pointed out, only using birth weight does not fully capture the true growth level of the fetus. Indeed, the uterus has a remarkable ability to expand and adapt during a multiple pregnancy. Although there is a higher rate of preterm birth among twins who may have lower birth weight compared to single births, it does not mean that all low birth weight (LBW) twin fetuses have experienced poor growth. Lower birth weight may mean the growth adaptation of fetuses in multiple pregnancy (*Blickstein. Semin Neonatol 2002*). These physiologically adapted LBW fetuses may not have a noticeable effect on adult disease. However, the potential to increase the volume and nutritional capacity of the uterus is limited, especially for twin pregnancy. Failure in the fetal adaptation process in utero results in significant growth discordance and restriction (*Blickstein. Best Pract Res Clin Obstet Gynaecol 2004*), which may lead to LBW fetuses. Inappropriate fetal growth may have implications for the long-term effects. Thus, twins with LBW may have a proper fetal growth or actual growth restriction. As the reviewer commented, we have further discussed this point (page 14, lines 10-18) and acknowledged the limitation on gestation age (page 15, lines 11-14) as follows.

"Twins have a unique and highly distinctive pattern of fetal growth. Although there is a higher rate of preterm birth among twins[44] who may have lower birth weight compared to single births, a preterm fetus with LBW may have appropriate fetal growth. Actual growth restriction could occur when twins fail to adapt to an intrauterine environment. Fetal malnutrition or inappropriate growth in gestation may redirect scant energy supplies from muscle to vital tissues, causing permanent alterations in physiology, metabolism, and structure.[45,46] Nevertheless, LBW alone could not fully capture the true growth level of the fetus, and monitoring the entire period of twin pregnancy is necessary to clarify the mechanism between LBW and CMDs in twins."

"Third, data on gestational age and other prenatal factors (such as maternal smoking during pregnancy, premature birth, or parental socioeconomic status) were not available and could not be fully controlled for."

Reviewer: 4
Dr. Rachana Singh, Tufts Medical Center
Comments to the Author:
I applaud the authors for detailed rebuttal to the reviewer's feedback and addressing them in the revised manuscript.

We thank the reviewer again for the helpful comments.

VERSION 3 – REVIEW

**REVIEWER**	Helen Bailey<br>Universite Paris Descartes, UMRS-1153 Equipe 7 (EPICEA), INSERM,
**REVIEW RETURNED**	31-May-2021

**GENERAL COMMENTS**	I thank the authors for their response and accept their modifications to the paper. However, the implication that co-twin matched analyses overcome the issue of the difference between poor fetal growth and low birth weight as the twins share the same gestation and environment is incorrect. Only one preterm twin can have SGA, although both are low birth weight (1). To avoid having to do multiple revisions of future papers, I suggest that the authors collaborate with perinatal epidemiologists/clinicians<br><br>1. D'Antonio F, Odibo AO, Prefumo F, Khalil A, Buca D, Flacco ME, et al. Weight discordance and perinatal mortality in twin pregnancy: systematic review and meta-analysis. Ultrasound Obstet Gynecol. 2018;52(1):11-23.
